# Assessment of surveillance core and support functions regarding neglected tropical diseases in Kenya

**DOI:** 10.1186/s12889-021-10185-1

**Published:** 2021-01-15

**Authors:** Arthur K. S. Ng’etich, Kuku Voyi, Clifford M. Mutero

**Affiliations:** 1 School of Health Systems and Public Health (SHSPH), University of Pretoria, Pretoria, South Africa; 2 University of Pretoria Institute for Sustainable Malaria Control (UP ISMC), University of Pretoria, Pretoria, South Africa; 3 International Centre of Insect Physiology and Ecology, Nairobi, Kenya

**Keywords:** Surveillance and response systems, Core functions, Support functions, Neglected tropical diseases

## Abstract

**Background:**

Effective surveillance and response systems are vital to achievement of disease control and elimination goals. Kenya adopted the revised guidelines of the integrated disease surveillance and response system in 2012. Previous assessments of surveillance system core and support functions in Africa are limited to notifiable diseases with minimal attention given to neglected tropical diseases amenable to preventive chemotherapy (PC-NTDs). The study aimed to assess surveillance system core and support functions relating to PC-NTDs in Kenya.

**Methods:**

A mixed method cross-sectional survey was adapted involving 192 health facility workers, 50 community-level health workers and 44 sub-national level health personnel. Data was collected using modified World Health Organization generic questionnaires, observation checklists and interview schedules. Descriptive summaries, tests of associations using Pearson’s Chi-square or Fisher’s exact tests and mixed effects regression models were used to analyse quantitative data. Qualitative data derived from interviews with study participants were coded and analysed thematically.

**Results:**

Surveillance core and support functions in relation to PC-NTDs were assessed in comparison to an indicator performance target of 80%. Optimal performance reported on specimen handling (84%; 100%), reports submission (100%; 100%) and data analysis (84%; 80%) at the sub-county and county levels respectively. Facilities achieved the threshold on reports submission (84%), reporting deadlines (88%) and feedback (80%). However, low performance reported on case definitions availability (60%), case registers (19%), functional laboratories (52%) and data analysis (58%). Having well-equipped laboratories (3.07, 95% CI: 1.36, 6.94), PC-NTDs provision in reporting forms (3.20, 95% CI: 1.44, 7.10) and surveillance training (4.15, 95% CI: 2.30, 7.48) were associated with higher odds of functional surveillance systems. Challenges facing surveillance activities implementation revealed through qualitative data were in relation to surveillance guidelines and reporting tools, data analysis, feedback, supervisory activities, training and resource provision.

**Conclusion:**

There was evidence of low-performing surveillance functions regarding PC-NTDs especially at the peripheral surveillance levels. Case detection, registration and confirmation, reporting, data analysis and feedback performed sub-optimally at the facility and community levels. Additionally, support functions including standards and guidelines, supervision, training and resources were particularly weak at the sub-national level. Improved PC-NTDs surveillance performance sub-nationally requires strengthened capacities.

**Supplementary Information:**

The online version contains supplementary material available at 10.1186/s12889-021-10185-1.

## Background

Health systems building blocks derived from the World Health Organization (WHO), work in concert with one another and are key to achieving health system strengthening [1]. Health Information Systems (HIS) are a particularly critical building block providing evidence-based and technological structures to collect, analyse and interpret data in order to generate useful information on patterns and determinants of disease occurrence for action by policy makers, health managers and personnel [2]. Lack of adequate disease-related information hinders health ministries from developing and strengthening health systems capacities for future needs [3]. Public health surveillance is a key component of HIS involving the continuous collection, analysis and interpretation of health data resulting in the timely dissemination of information to enable effective public health action [4]. Effectiveness of surveillance systems is dependent on continued improvement of their performance in detecting and responding to diseases, which can be achieved through ensuring accurate and timely reporting for effective response to identified cases and outbreaks [5]. In September 1998, an Integrated Disease Surveillance (IDS) strategy, which was later renamed Integrated Disease Surveillance and Response (IDSR) system, was adopted by member states of the World Health Organization Regional Office for Africa (WHO-AFRO). This strategy aimed to ensure that action oriented, integrated and district-focused public health surveillance systems are in place [6–8]. An effective disease surveillance and response system requires optimal functioning of core activities including case detection, registration and confirmation, reporting, data analysis, feedback and outbreak response. On the other hand, sustained core function performance necessitates support from adequate standards and guidelines, supervision, training and resources [9].

Strengthening national disease surveillance and response systems requires periodic evaluation of the structure, functions and attributes of the system to review strengths, weaknesses, and opportunities for improvement [10]. In Kenya, the IDSR system was adopted in the year 2000 and core capacity assessment undertaken in the same year [11]. In 2009, an external review was conducted with the aim of assessing the performance of Vaccine Preventable Diseases (VPD) surveillance within the IDSR framework [11]. Findings from the assessment revealed strong surveillance structures were in place and the availability of IDSR technical guidelines and case definitions at all levels. Recommendations resulting from the assessment led to increased training on IDSR and budget line creation for surveillance and response activities [11]. However, recommendations based on assessment of surveillance and response systems are focused on tackling notifiable diseases thereby overlooking neglected tropical diseases (NTDs), which cause serious debilitating effects among the affected communities. NTDs in recent years have gained public health importance and their elimination by 2030 is a key target within the Sustainable Development Goals (SDGs) and attaining Universal Health Coverage [12, 13]. Mass drug administration being the most pragmatic approach to combating NTDs faces consistent implementation challenges relating to hampered drug delivery strategies, inadequate number of community drug distributors, vast areas of drug coverage and unclear geographical boundaries [14]. Hence, to address these challenges given scarce resources requires targeted interventions. In addition, NTD endemic regions face variations in local health systems performance among other factors. This poses a foreseeable challenge to maintaining NTDs elimination goals beyond 2020 especially in ascertaining local elimination of disease transmission [15]. Therefore, there is need to strengthen surveillance and response systems focused on NTDs control and elimination in order to inform targeted treatment and meet post-elimination goals. Key to addressing NTDs endgame challenges is to strengthen post-control surveillance efforts by developing new and improving existing monitoring tools and ensuring reporting systems function effectively [16]. A shift from donor dependence to more sustainable approaches through strengthened national health systems is vital to achieving NTDs elimination goals [17]. NTDs technical and programmatic challenges coupled to the changing donor and partner landscape rationalise the need for increased efforts to strengthen existing health systems [17].

NTDs mainly burden health systems at the sub-national level [17]. Hence, requiring surveillance technical and operational strategies to be well adapted to the sub-national context and resource capacity [17]. Additionally, operational research to inform surveillance and response strategies grounded on local settings are crucial to identifying research priorities regarding disease elimination [18]. In line with efforts of ‘leaving no one behind’ it would require strengthened surveillance capacity to identify NTDs transmission foci that will ensure interventions and health services reach the most afflicted communities [13, 17]. The IDSR system categorised targeted diseases in to three main groups including those of a notifiable nature, diseases targeted for eradication or elimination and other disease conditions or events of public health importance [8]. However, there is paucity of knowledge on surveillance system core and support functions assessment with a focus on diseases targeted for elimination and eradication in accordance with the revised IDSR guidelines. Focus on notifiable disease conditions was quite evident from previous systematic literature reviews, with the reviewed studies assessing surveillance core, support and attribute functions of the IDSR system mostly based on notifiable diseases [19, 20]. Specifically in Kenya, a number of NTDs are earmarked for eradication or elimination or considered major diseases, events or conditions of public health importance [11, 21]. The NTDs include Guinea Worm Disease (GWD), Leishmaniasis, Schistosomiasis and Trachoma. Most of these NTDs are prioritised for monthly reporting except for GWD, which is included in the IDSR epidemic monitoring form routinely utilised for weekly reporting at the health facility level in Kenya. To date, there is no systematic assessment of the existing IDSR system functions with regard to NTDs especially at the surveillance operational level, i.e. sub-national level in Kenya.

The IDSR strategy is comprehensive and comprising of diverse activities to enable effective disease detection to inform appropriate public health action. Four principal elements namely structural, core activities, support activities and attribute functions constitute the IDSR framework. However, this study delineated and focused on the surveillance core and support functions, which form the main driving force to implement the strategy effectively. In addition, bearing in mind the consolidated principle of the IDSR strategy that aims to accommodate all diseases under surveillance, this study assessed the performance of surveillance core and support activities specific to neglected tropical diseases. Assessment of the surveillance functions relating to PC-NTDs was based on components of the system as documented in the WHO guideline [5]. Definitions for surveillance core and support functions were derived from the proposed framework for communicable disease surveillance by World Health Organization and Centers for Disease Control and Prevention (CDC) updated guideline for evaluating public health surveillance systems [5, 22]. The core functions are based upon indicators that measure the system processes and outputs [23]. These include case detection, registration and confirmation of health related events, reporting, analysis and interpretation of surveillance data, outbreak response and feedback to surveillance system users and decision makers. In view of reporting as a core surveillance function, “zero” reporting was defined as the process whereby health workers correctly provide a null report by clearly indicating a ‘zero’ whenever there were no NTD cases to report at a given time instead of submitting incomplete reporting forms resulting to missed data. On the other hand, support functions guide and facilitate implementation of the core functions including; standards and guidelines, training, supervisory activities, communication facilities, resources, coordination, monitoring and evaluation [23]. Therefore, this study aimed to assess the core and support functions of the existing IDSR system in relation to four preventive chemotherapy-targeted NTDs (PC-NTDs) of known endemicity and prioritised for control or elimination in Kenya including Soil Transmitted Helminths, Schistosomiasis, Trachoma and Lymphatic Filariasis [24–26]. Improved surveillance and response to PC-NTDs will ensure timely disease detection and lead to appropriate evidence-based public health action towards achieving disease elimination.

## Methods

### Study setting and population

**Fig. 1:**
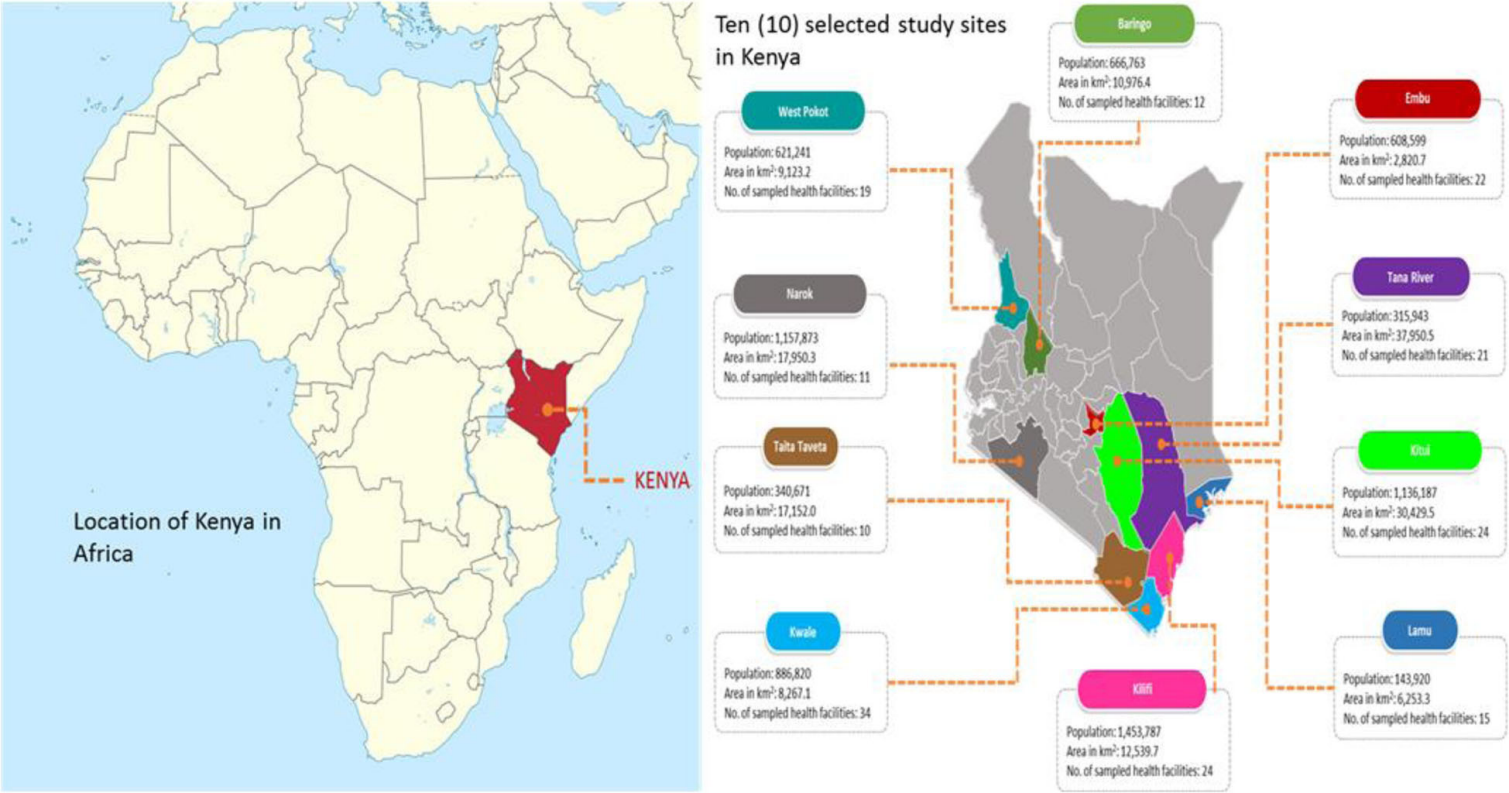
Map showing the location of Kenya in Africa [27]; Map showing the sampled study sites in Kenya (This figure was downloaded from SlideModel.com [28], modified using Microsoft Powerpoint 2016 and the source data retrieved from the 2019 Kenya Population and Housing Census Volume I: Population by County and Sub County [29]). No permission was required to use or modify the map images

Kenya is situated in East Africa and bordered by Uganda to the West, Somalia to the East, South Sudan and Ethiopia to the North and Tanzania to the South (Fig. 1).

The country comprises of 47 administrative counties and up to 290 sub-counties. The study areas included endemic counties with at least one sub-county having three co-endemic PC-NTDs of known prevalence (Table 1) [24]. According to data provided in the second national strategic plan for control of neglected tropical diseases in Kenya and the Demographic Health Information System (DHIS2), the PC-NTDs endemic counties meeting this criteria and representing regions highly endemic of the diseases in Kenya included; Baringo, Narok, West Pokot, Kwale, Kilifi, Lamu, Tana River, Taita Taveta, Kitui and Embu counties [24]. The study sites are in three distinct geographical regions namely; Rift Valley (Baringo, Narok and West Pokot), Coast (Kwale, Kilifi, Lamu, Tana River and Taita Taveta) and Eastern (Kitui and Embu) based on previous regional boundaries in Kenya (Fig. 1). A common ecological characteristic of the study sites was that of arid and semi-arid conditions with the populous mostly settled in remote parts of the study areas.

**Table 1:** Known distribution of PC-NTDs in study sites

Region	Counties	Sub-counties	n (%)	Co-endemic ntds				Prevalence rates			
				LF	SCH	STH	TRC	LF	SCH	STH	TRC
Rift valley	Baringo	Marigat	12 (100%)	–	X	X	X	–	2.4%^b^	17.7%	12.8%^C^
	Narok	Trans Mara West	11 (100%)	–	X	X	X	–	1.2%^b^	39%	14.9%^C^
	West Pokot	Kacheliba	8 (42%)	–	X	X	X	–	0.3%^b^	6%	26.6%^C^
		Pokot West	11 (58%)								
Coastal	Kwale	Kinango	9 (27%)	X	X	X	–	1%	17.8%^a^	4%	–
		Lungalunga	9 (27%)								
		Matuga	13 (38%)								
		Msambweni	3 (9%)								
	Kilifi	Kaloleni	7 (29%)	X	X	X	–	3%	11.3%^a^	3.8%	–
		Kilifi North	6 (25%)								
		Kilifi South	6 (25%)								
		Malindi	5 (21%)								
	Lamu	Lamu West	15 (100%)	X	X	X	–	6%	10%^a^	3%	–
	Tana River	Galole	7 (33%)	X	X	X	–	2%	55%^a^	4%	–
		Garsen	14 (67%)								
	Taita Taveta	Taveta	10 (100%)	X	X	X	–	3%	10%^a^	4%	–
Eastern	Kitui	Kitui Central	18 (75%)	–	X	X	X	–	3.8%^b^	8.4%	4.8%^C^
		Mwingi	6 (25%)								
	Embu	Runyenjes	22 (100%)	–	X	X	X	–	4%^b^	10%	0.2%^C^

n - Number of sampled healthcare facility units in a sub-county; X – Indicates presence of an endemic NTD; ^a^ – Prevalence of Schistosomiasis (*Schistosoma haematobium*); ^b^ – Prevalence of Schistosomiasis (*Schistosoma mansoni*); ^c^ – prevalence of Trachomatous Follicular (TF) Inflammation; *LF* Lymphatic Filariasis, *SCH* Schistosomiasis, *STH* Soil Transmitted Helminths, *TRC* Trachoma

Source Data: The 2nd National Strategic Plan for Control of Neglected Tropical Diseases 2016–2020 [24] & Kenya Landscape Analysis for Neglected Tropical Diseases, WASH and Behaviour Change [30]

The study targeted healthcare personnel responsible for collection and transmission of surveillance data at the following three levels;

*Community level:* Community health extension workers (CHEWs) responsible for compiling and reporting disease data and assigned to fully functional community health units (CHUs) linked to selected health facilities.

*Health facility level:* Healthcare facility workers (HFWs) in both public and private facilities responsible for collection and transmission of surveillance data. HFWs were drawn from various facility levels including dispensaries (level 2), basic primary health care facilities (level 3), primary care hospitals (level 4) and county referral hospitals (level 5) that reported a high-threshold of PC-NTD cases in the one-year surveillance period.

*Sub-national health management teams:* Healthcare personnel at the sub-county and county levels responsible for collation and transmission of surveillance data reported from the healthcare facilities in the given surveillance period. Also included in this category were key healthcare stakeholders responsible for decision-making and overseeing surveillance and response related activities including county directors of health, county epidemiologists, county NTDs coordinators and county health information managers.

### Surveillance system functioning

**Fig. 2:**
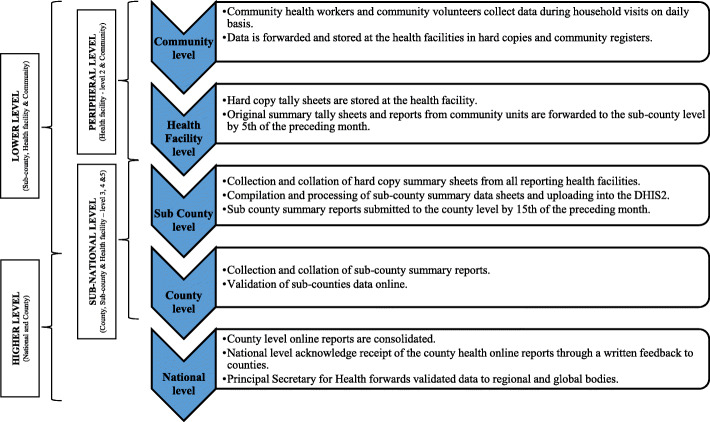
Surveillance Data Flow in Kenya

At the community level, community health volunteers complete relevant reporting forms either monthly or biannually to capture the monthly services offered, health and demographic information of the households. These reports are submitted to CHEWs to undertake data verification, consolidation and data summaries for onward submission to the health facility chalkboard and further to the upper levels for entry into the DHIS2 [31]. The requirement for reporting weekly IDSR data at the health facility level is submission of original copy of reports by Monday of the following week [11, 32]. The health worker concerned can either submit weekly IDSR data through hardcopy report delivery or verbally through a phone call. However, other common alternative methods of submitting weekly reports include Short Message Services (SMSs), emails or through WhatsApp messaging application. A copy of the report is retained at the health facility level while the original copy is submitted to the sub-county level. The sub-county disease surveillance and response coordinator (SCDSRC) then collates reports from all reporting health facilities and uploads online through the DHIS2 by Wednesday of the following week. On the other hand, monthly summary IDSR data from health facilities require submission to the sub-county level by the 5th day of the following month following which the SCDSRC enter the data into the DHIS2 before 15th of the following month [11]. The county disease surveillance and response coordinators (CDSRCs) and county health information and records officers (CHIROs) verify and validate data entered into the DHIS2 before the information is available to health system end-users (Fig. 2).

### Study design and sampling procedure

A mixed method cross-sectional survey approach was used to assess the IDSR system surveillance functions comprising; core and support functions. The study involved the concurrent collection of both quantitative and qualitative data. The convergence method employed was aimed at ensuring detailed data was captured from the study participants while giving equal importance in terms of analysis and comparison to both types of data [33]. Surveillance system assessment was guided by the WHO protocol for assessing national surveillance system and the CDC updated guidelines for evaluating public health surveillance systems [22, 23].

**Fig. 3:**
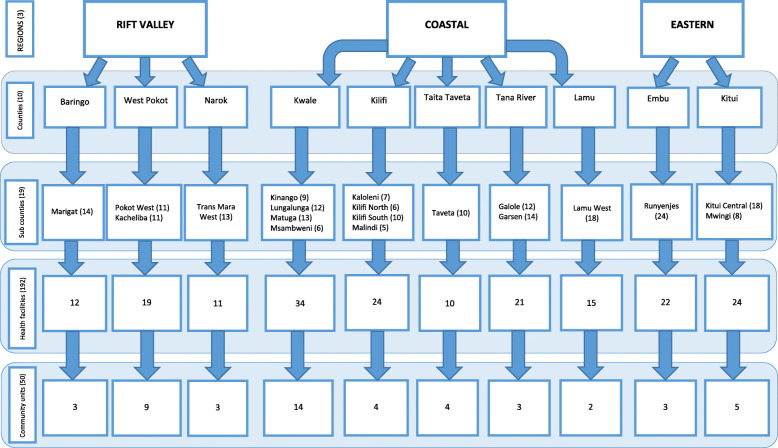
Sampling procedure

Study areas were purposively sampled from the Rift Valley, Eastern and Coastal parts of Kenya, which are regions endemic of fully mapped PC-NTDs. These regions were selected to enable detailed assessment of NTDs surveillance and response activities within the IDSR system framework. Within the three regions, ten (10) administrative counties prevalent of at least three or more PC-NTDs were purposively sampled. Furthermore, within the counties, nineteen (19) sub-counties reporting a high number of PC-NTD cases in the one-year surveillance period (2017) were selected (Table 1). This was followed up by purposive sampling of healthcare facilities and linked fully functional community health units that reported high-threshold levels of PC-NTD cases in the 2017 surveillance period in the selected endemic sub-counties (Fig. 3).

There were 341 operational healthcare facilities providing PC-NTDs control and preventive services in the selected endemic counties according to data obtained from the Kenya Master Health Facility List (KMHFL) and Kenya Service Availability and Readiness Mapping (SARAM) Report [34, 35]. Two hundred and twenty-one of these operational facilities were located in the selected sub-counties prevalent of at least three PC-NTDs of known endemicity. Of the 221 healthcare facilities, those that had reported high-threshold levels of PC-NTD cases in the 2017 surveillance period according to data retrieved from the DHIS2 system were included in the study. The cut off for identifying health facilities reporting high-threshold levels of PC-NTD cases was determined by calculating the average number of cases reported for a specific disease within a one-year period. The cumulative total of reported cases by each facility in the one-year surveillance period was divided by the total number of health facilities in the selected study site. Therefore, 192 out of 221 facilities reported above the mean number of reported cases for all the co-endemic PC-NTDs in the 2017 surveillance period and were identified as high-threshold reporting facilities. An equivalent of 192 healthcare workers responsible for surveillance data collection and transmission in the selected health facilities were included in the study. Moreover, there were 50 fully functional community health units linked to the selected healthcare facilities according to data obtained from the KMHFL [34]. Therefore, healthcare personnel in-charge of the fully functional CHUs linked to 50 out of the 192 healthcare facilities were enrolled in the study. Similarly, healthcare personnel in-charge of disease surveillance activities at the sub-counties and county levels were purposively sampled. This comprised of sub-county disease surveillance and response coordinators (*n* = 19) and county disease surveillance and response coordinators (*n* = 10). Furthermore, fifteen (15) key stakeholders overseeing disease surveillance activities at the sub-national level were also enrolled. Purposive sampling technique was largely preferred since it enabled careful selection of specific “information rich” participants across the surveillance levels to gain extensive knowledge on their experiences [36, 37]. Selection of experienced respondents was vital to obtain convincing non-random outcomes [38]. Therefore, in the current study, respondents were purposively sampled based on their direct involvement in surveillance activities at the sub-national level.

### Data collection and analysis

Semi-structured questionnaires were used to obtain information from healthcare personnel responsible for surveillance data collection and transmission at the community (CHEWs), healthcare facility (HFWs), sub-county (SCDSRC) and county (CDSRC) levels based on their availability during the study (see Supplementary file 1). The semi-structured questionnaires gathered information on IDSR core and support functions’ performance relating to PC-NTDs across the surveillance levels. The questionnaires comprised of participants’ demographic information with questions made relevant to the specific surveillance level and administered in the most appropriate language to the participants, which was either English or Swahili. Additionally, interview schedules were used to conduct key informant interviews with healthcare personnel overseeing disease surveillance related activities in the PC-NTD endemic counties including county directors of health, county epidemiologists and county health information and records officers. Data was collected between November 2017 and June 2018.

Data entry was conducted using Epi Info Version 7 and later the data was imported into Stata/IC 14.0 (College Station, 77,845 Texas USA) for further statistical analysis. Frequency computation and summary tables of healthcare workers’ socio-demographic characteristics at the sub-national level were undertaken. Categorical variables relating to questionnaire items on IDSR system core and support functions regarding PC-NTDs were summarised using frequencies. Bivariate analysis between categorical variables and HFWs socio-demographic characteristics and other facility surveillance activities were assessed using Pearson’s Chi Square test. Additionally, where assumptions for use of Pearson’s Chi Square test were violated we resorted to using the Fisher’s Exact test. Independent variables associated with the outcome in the bivariate analysis (i.e. with *p*-value < 0.05) were incorporated in a mixed effects logistic regression models. Bivariate logistic regression models were implemented to determine the crude estimates of the effects of independent variables before multivariable (adjusted) mixed effects models were fitted. The adjusted models excluded independent variables with *p*-value > 0.05 at the bivariate level of analysis. The logistic regression models were interpreted by the adjusted odds ratio (AOR) with a 95% confidence interval (CI), and the corresponding p-value. Qualitative data was managed using ATLAS.ti version 8 qualitative data analysis software [39]. Research participants’ verbal responses to the questionnaire open-ended questions were transcribed and coded into various themes of the surveillance system core and support functions. Qualitative data analysis identified the main themes based on surveillance functions that required strengthening while the emerging codes identified key recommendations to improving the surveillance functions relating to neglected tropical diseases rated according to the codes groundedness [40].

## Results

### Study participants’ socio demographic information

Two hundred and ninety-five health personnel were enrolled in the study with a response rate of 96%. Excluding those who did not give their consent (*n* = 12), comprising community health workers (*n* = 5) and health facility workers (*n* = 7), the final sample size was 283. This comprised of community (50, 18%), health facility (192, 68%), sub-county (19, 7%) and county (10, 3%) level health personnel and other key informants (12, 4%). Overall, majority (89%, 251/283) of the study participants were aged over 30 years with a preponderance (58%, 165/283) of male participants. Forty-one percent (116/283) of study participants had more than 5 years working experience in their current designation with 93% (264/283) having at least attained a diploma or a higher level of education (Tables 2, 3, 4, 5 & 6).

**Table 2:** Socio-demographic information for health facility respondents

Characteristic	n (%)
County region	
Rift Valley	42 (22%)
Coastal	104 (54%)
Eastern	46 (24%)
Health facility level	
Level 2 - Dispensary	136 (71%)
Level 3 - Health Centre	42 (22%)
Level 4 - Sub County Hospital	12 (6%)
Level 5 - County Referral Hospital	2 (1%)
Health facility type	
Private	23 (12%)
Public	169 (88%)
Age of the respondent	
18–30 years	26 (14%)
31–40 years	91 (47%)
41–50 years	60 (31%)
> 50 years	15 (8%)
Gender of the respondent	
Female	98 (51%)
Male	94 (49%)
Health cadre	
Clinical Officer	43 (22%)
Health Records Management Staff	3 (2%)
Laboratory Staff	1 (1%)
Nursing Staff	125 (65%)
Public Health Staff	20 (10%)
Years of experience in the health cadre	
1 - < 2 years	13 (7%)
2 - < 3 years	53 (28%)
3 - ≤5 years	56 (29%)
More than 5 years	70 (36%)
Highest level of education	
Certificate	13 (7%)
Diploma	159 (83%)
Degree	20 (10%)

**Table 3:** Socio-demographic information for community health units and community health extension workers

Characteristic	n (%)
County location for CHUs	
Rift Valley	15 (30%)
Coastal	27 (54%)
Eastern	8 (16%)
Health facility level linked to CHUs	
Level 2 – Dispensary	30 (60%)
Level 3 - Health Centre	15 (30%)
Level 4 - Sub County Hospital	5 (10%)
Health facility type linked to CHUs	
Private	3 (6%)
Public	47 (94%)
Age of CHEWs	
18–30 years	6 (12%)
31–40 years	19 (38%)
41–50 years	22 (44%)
> 50 years	3 (6%)
Gender of CHEWs	
Female	13 (26%)
Male	37 (74%)
Health cadre of CHEWs	
Laboratory Staff	2 (4%)
Nurse	7 (14%)
Public Health Staff	41 (82%)
Years of work experience as CHEWs	
1 - < 2 years	2 (4%)
2 - < 3 years	7 (14%)
3 - ≤5 years	20 40%)
More than 5 years	21 (42%)
Highest level of education of CHEWs	
Certificate	6 (12%)
Diploma	39 (78%)
Degree	5 (10%)

*CHU* Community Health Units

*CHEWs* Community Health Extension Workers

**Table 4:** Socio-demographic information for sub county disease surveillance coordinators

Characteristic	n (%)
County region	
Rift Valley	4 (21%)
Coastal	12 (63%)
Eastern	3 (16%)
Age of the respondent	
31–40 years	7 (37%)
41–50 years	9 (47%)
> 50 years	3 (16%)
Gender of the respondent	
Female	4 (21%)
Male	15 (79%)
Highest level of education	
Degree	7 (37%)
Diploma	12 (63%)
Years of work experience in current designation	
3 - ≤5 years	6 (32%)
More than 5 years	13 (68%)

**Table 5:** Socio-demographic information for county disease surveillance coordinators

Characteristic	n (%)
County region	
Rift Valley	3 (30%)
Coastal	5 (50%)
Eastern	2 (20%)
Age of the respondent	
41–50 years	6 (60%)
> 50 years	4 (40%)
Gender of the respondent	
Female	1 (10%)
Male	9 (90%)
Highest level of education	
Degree	6 (60%)
Diploma	4 (40%)
Years of work experience in current designation	
3 - ≤5 years	3 (30%)
More than 5 years	7 (70%)

**Table 6:** Socio-demographic information for key informants

Characteristic	n (%)
County region	
Rift Valley	7 (58%)
Coastal	3 (25%)
Eastern	2 (17%)
Age of the respondent	
31–40 years	2 (17%)
41–50 years	7 (58%)
> 50 years	3 (25%)
Gender of the respondent	
Female	2 (17%)
Male	10 (83%)
Highest level of education	
Masters	2 (16%)
Degree	10 (84%)
Designation of key informant	
County Director of Health	3 (25%)
County Epidemiologist	2 (17%)
County Health Records and Information Officer	5 (42%)
County NTDs Coordinator	2 (17%)
Years of work experience in current designation	
3 - ≤5 years	7 (58%)
More than 5 years	5 (42%)

### Study participants’ involvement in surveillance activities

**Fig. 4:**
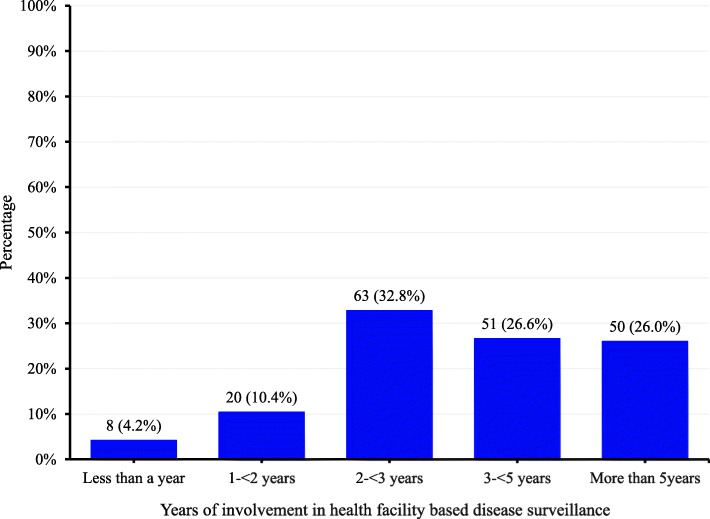
Distribution of respondents by the years of involvement in health facility based disease surveillance

The data showed that in their current cadre, up to 53% (101/192) of health facility respondents had been involved in health facility based disease surveillance for at least 3 years (Fig. 4). On the other hand, 86% (43/50) of respondents at the community level reported to be involved in disease surveillance activities.

Eighty-four percent (161/192) of facility respondents reported having a functional health facility-based surveillance system with the capacity to detect, confirm, report, analyse and interpret surveillance data to inform response actions. Availability of a laboratory equipped to confirm PC-NTDs cases, provision for PC-NTDs in the reporting forms and training of personnel on disease surveillance were associated with higher odds of having a functional health facility-based surveillance system [AOR = 3.07, 95% CI: 1.36, 6.94, *p* = 0.007], [AOR = 3.20, 95% CI: 1.44, 7.10, *p* = 0.004] and [AOR = 4.15, 95% CI: 2.30, 7.48, *p* < 0.001] respectively. Of the facilities with a functional health facility-based surveillance system, 64% (104/161) confirmed to report PC-NTDs through this system and up to 97% of respondents reported that it was important to have a PC-NTD surveillance system at the facility level. Majority (95%, 182/192) of facility respondents were well aware of PC-NTDs prevalent in the region. Twenty-three percent (45/192) of facility respondents identified Schistosomiasis and Soil Transmitted Helminths as the most common co-endemic conditions while 22% (42/192) reported Lymphatic Filariasis, Schistosomiasis and Soil Transmitted Helminths to commonly co-occur at any given time. At the community level, 88% (44/50) of respondents were aware of the PC-NTDs prevalent in the study regions and 80% of them had identified and reported at least one PC-NTD case at the community level in the previous year.

### Case detection, registration and guidelines

All sub-national levels (sub-counties and counties) were provided with IDSR standard case definition guidelines. Fifty-three percent and 70% of respondents reported to use the available standard case definitions to detect at least one PC-NTD in the previous year in the sub-county and county levels respectively (Table 7). Fifty percent (25/50) of community level respondents indicated that the surveillance guidelines were useful for PC-NTDs case detection. At the health facility level, 83% (159/192) of the respondents reported that standard case definitions for all diseases were available for use. Up to 60% of health facility workers admitted that the available PC-NTDs case definitions were clear and easy to use. However, a health facility respondent remarked;

**Table 7:** Core surveillance activities performance relating to PC-NTDs

Core surveillance Activities	Indicators	IDSR Target	Community Level (***N*** = 50)	Health Facility Level (***N*** = 192)	Sub-County Level (***N*** = 19)	County Level (***N*** = 10)
		%	% [n/N]	% [n/N]	% [n/N]	% [n/N]
Case detection	Proportion provided with IDSR standard case definitions	80	66 [33/50]	83 [159/192]	100 [19/19]	100 [10/10]
	Proportion using standard case definitions to detect at least one PC-NTD	80	58 [29/50]	60 [115/192]	53 [10/19]	70 [7/10]
Case registration	Proportion using specific case registers for PC-NTDs registration	80	68 [34/50]	19 [36/192]	NA	NA
Case confirmation	Proportion with a functional laboratory	80	N/A	52 [100/192]	100 [19/19]	100 [10/10]
	Proportion with the capacity to collect and store PC-NTD specimens	80	N/A	41 [79/192]	84 [16/19]	100 [10/10]
	Proportion that sent samples to a higher-level laboratory of at least one PC-NTD	80	32 [16/50]	18 [35/192]	NA	NA
	Proportion that received reports on referred PC-NTD samples	80	32 [16/50]	80 [28/35^a^]	NA	NA
Reporting	Proportion having IDSR reporting forms always available	80	74 [37/50]	89 [170/192]	79 [15/19]	80 [8/10]
	Proportion that reported at least one PC-NTD case	80	78 [39/50]	84 [162/192]	100 [19/19]	100 [10/10]
	Proportion that undertook zero reporting of at least one PC-NTD	80	N/A	81 [156/192]	100 [19/19]	100 [10/10]
	Proportion that met deadlines for submitting PC-NTDs surveillance reports	80	N/A	88 [143/162^b^]	84 [16/19]	80 [8/10]
Data analysis	Proportion that analysed data of at least one PC-NTD	80	42 [21/50]	58 [111/192]	84 [16/19]	80 [8/10]
	Proportion that undertook trend analysis of at least one PC-NTD	80	N/A	44 [49/111^c^]	26 [5/19]	60 [6/10]
	Proportion with action thresholds of at least one PC-NTD	80	N/A	29 [56/192]	74 [14/19]	80 [8/10]
Feedback	Proportion that received feedback from higher-level of at least one reported PC-NTD	80	54 [27/50]	36 [70/192]	53 [10/19]	60 [6/10]
	Proportion that received at least one written feedback report from the higher level of a reported PC-NTD case	80	N/A	80 [56/70^d^]	N/A	N/A
Epidemic preparedness and response	Proportion with a rapid response team	80	N/A	NA	63 [12/19]	100 [10/10]
	Proportion with adequate outbreak response supplies	80	N/A	NA	63 [12/19]	100 [10/10]

NA – indicator was either not available or unmeasurable at the specific surveillance level

^a,b,c,d^ - denominators are derived from totals of a preceding affirmative outcome

*“There was a time we came across a suspected trachoma case at the onset and it was difficult to apply the provided case definition guidelines … symptoms of redness and teary eyes are at times as a result of other allergic reactions … we needed more clear case definitions to accurately identify a trachoma case especially at the acute stages before follicles are visible” –* HFW#118 (Kitui County).

Sixty-eight percent (34/50) of community level respondents reported to register identified PC-NTD cases. At facility level, 19% of respondents reported that specific case registers for PC-NTDs were available. However, these facilities were mainly designated NTDs treatment centres. Further, facility respondents reported that most disease conditions were routinely recorded in a common outpatient register;

*“We record all disease cases in a common register … so there is no separate register for certain specific diseases … may be such registers are present in the health facilities that mostly see and treat patients with diseases such as Trachoma” –* HFW#009 (Baringo County).

Less than half (48%, 93/192) of the respondents reported that manuals for disease surveillance were available at the facility. Of this fraction, 75% reported that the manuals were up-to-date, 89% reported that the manuals were useful in guiding disease surveillance activities, and 61% reported that the available manuals specifically guided PC-NTDs surveillance activities at the health facility level. Health workers recommended the need for case registers specific for registering PC-NTDs cases to ensure there is a clear log of reported cases starting from the peripheral to county levels;

*“By having specific case registers for NTDs that are being utilised right from the peripheral level...will help track disease occurrences … suspected cases can always be followed up if they are well registered and a proper record is kept” –* CHEW#031 (Kwale County).

### Case confirmation

At the sub-national level, 84% of sub-counties had the capacity to collect and store PC-NTD specimens (Table 7). Thirty-two percent (16/50) of community level respondents reported to have referred collected samples of at least one PC-NTD to the health facility level in the previous year. An equivalent proportion (32%) of respondents reported to receive case confirmation reports on the referred specimens. Of this number, 88% of them indicated that they had referred urine samples for suspected cases of urinary schistosomiasis. On the other hand, 52% (100/192) of health facility respondents reported presence of a functional laboratory with 51% of them indicating that the laboratories were adequately equipped to confirm PC-NTD cases. However, direct observations revealed that only about 22% (43/192) of health facility laboratories were adequately equipped to confirm PC-NTDs. Up to 41% (79/192) of respondents reported that the health facilities were able to handle PC-NTD specimens, and 46% reported that the facilities had capacity to transport the specimens to higher-level laboratories. Further, 18% (35/192) of respondents reported that the facilities sent PC-NTD specimens to higher-level laboratories in the past year with 80% of this fraction reporting that the facilities received specimen feedback reports. Respondents recommended the need to strengthen laboratory capacity to confirm suspected PC-NTD cases. Further recommendations alluded to provision of adequate laboratory personnel, reagents, and equipment;

*“We require fully equipped laboratories with trained laboratory personnel at the community level which mostly lack capacity to confirm suspected NTDs cases … we lack adequate resources to transport specimens … patients also lack money to cover for transport costs when referred to the facility” –* HFW#161 (Tana River County).

### Reporting

Eighty-four percent and 80% of sub-national levels met deadlines for submission of PC-NTDs surveillance reports at the sub-county and county levels respectively (Table 7). Forty percent (20/50) of community level respondents reported lack of reporting forms at some point in the past six months with 78% having reported at least one PC-NTD case in the past year and 76% having referred the identified PC-NTD case to the health facility level. Slightly more than half (55%) of health facility respondents reported that the forms had provision for reporting PC-NTDs. Of this fraction, 61% indicated that the provision for reporting PC-NTDs was sufficient. Facility respondents (39%) who felt that the reporting form provision was insufficient, attributed their reasons to lack of PC-NTDs inclusion in the forms;

*“NTDs are in extension still neglected even in the available IDSR reporting forms … seeing that NTDs are not included in summary forms clearly indicates lack of priority … these diseases need to be listed in the forms similar to other common conditions to ease reporting” –* HFW#094 (Kilifi County).

Results showed that 84% (162/192) of respondents were aware of deadlines for submission of PC-NTDs surveillance reports at the facility level with 88% of them confirming compliance with the reporting deadlines. Health workers of the nursing cadre (48%) were solely responsible for preparing PC-NTDs surveillance reports at the facility level. Further, 81% of facility respondents confirmed, “zero” reporting was undertaken when there were no PC-NTD cases to report at any given month. Findings from a logistic regression model assessing the predictors of “zero” reporting of PC-NTDs showed that respondents with longer years of work experience had higher odds of undertaking “zero” reporting compared to those who had served fewer years (Table 8). In addition, availability of PC-NTDs case definitions and availability of reporting forms were associated with increased odds of undertaking “zero” reporting; [AOR = 2.52, 95% CI: 1.01, 6.28; *p* = 0.048] and [AOR = 3.18, 95% CI: 1.10, 9.23; *p* = 0.033] respectively.

**Table 8:** Factors associated with “Zero” reporting for PC-NTDs

		Zero-reporting for PC-NTDS		Unadjusted Estimates		Adjusted Estimates	
Characteristic	N	No, n (%)	Yes, n (%)	OR (95% CI)	***p***-value	OR (95% CI)	***p***-value
Health cadre							
Nurse		24 (19.5%)	99 (80.5%)	1.00		1.00	
Clinical Officer	183	2 (5.3%)	36 (94.7%)	4.36 (1.13, 16.8)	0.032	6.11 (1.71, 21.8)	0.005
HRMS/LS/PHS		1 (4.5%)	21 (95.5%)	5.09 (1.31, 19.8)	0.019	5.66 (1.97, 16.3)	0.001
Years of work experience							
1- < 2 years		3 (23.1%)	10 (76.9%)	1.00		1.00	
2- < 3 years	183	13 (26%)	37 (74%)	0.85 (0.28, 2.64)	0.784	1.05 (0.25, 4.45)	0.946
3- ≤ 5 years		7 (13.0%)	47 (87.0%)	2.01 (0.38, 10.6)	0.409	2.26 (0.38, 13.6)	0.373
> 5 years		4 (6.1%)	62 (93.9%)	4.65 (1.80, 12.0)	0.001	6.11 (2.26, 16.5)	< 0.001
Availability of PC-NTDs case definitions							
No	175	15 (24.2%)	47 (75.8%)	1.00		1.00	
Yes		12 (10.6%)	101 (89.4%)	2.69 (1.16, 6.23)	0.021	2.52 (1.01, 6.28)	0.048
Availability of reporting forms							
No	183	7 (33.3%)	14 (66.7%)	1.00		1.00	
Yes		20 (12.3%)	142 (87.7%)	3.55 (1.22, 10.3)	0.020	3.18 (1.10, 9.23)	0.033

*N* Number of observations with valid data analysed, *OR* Odds Ratio, *95% CI* 95% Confidence Interval

The common channels for surveillance reports submission utilised by respondents at the facility level were through mobile phone short message services (SMSs) (81%) for weekly reporting and in person report submission (73%) for monthly summary reports. At the community level, 82% of respondents reported that the channel mostly used to transmit PC-NTDs monthly surveillance reports was in person submission of hardcopy forms to the health facilities. Respondents at the community level also reported using phone calls (64%) and mobile phone SMS (48%) to transmit surveillance data during weekly reporting. Respondents across the surveillance levels reported challenges using the available reporting channels;

*“Submitting reports via the DHIS2 portal is at times challenging given the inconsistent internet connectivity in the area … most times we have to incur the expenses resulting from purchase of internet bundles so as to access the portal during report submission” –* KII#003 (West Pokot County).

*“Long distances between the health facility and the next reporting level … and poor terrain in the region pose a challenge to delivering hardcopy monthly summary reports within the required time” –* SCDSRC#001 (Baringo County).

Health personnel mainly recommended improving PC-NTDs reporting within the IDSR system through provision of adequate resources to facilitate surveillance data reporting. Adopting electronic reporting tools through use of computers and mobile phone devices, and provision of financial incentives to cover for airtime, internet and transport costs;

*“If we had an electronic reporting system right from the facility level it would ease reporting and eliminate the burden of having to physically submit monthly summary reports” –* KII#001 (Baringo County).

Respondents further recommended provision of adequate reporting forms at any given time to ensure timely compilation of surveillance reports. In addition, providing improved monthly summary reporting forms with the inclusion of PC-NTDs to enhance their priority in surveillance reports;

*“The current reporting forms hardly include most of the NTDs … this makes it difficult to report the cases especially in the monthly summary reports … it would be better if most of the NTDs common in the region would be included in the reporting forms” –* HFW#112 (Kitui County).

Further recommendations alluded to enhanced training amongst health facility workers on the use of reporting tools and the need for health workers sensitisation on the benefits of effective surveillance reporting;

*“We need periodical training and capacity building on NTDs surveillance activities...health workers sensitisation on NTDs will improve reporting of the cases through the surveillance system” –* HFW#089 (Kilifi County).

### Data analysis

Surveillance data analysis for PC-NTDs at the health facility level was mainly either based on age and locality of the individual (27%), age and gender of the individual (23%) or solely based on the individuals’ age (24%). Among health facilities that conducted data analysis, 44% (49/111) performed trend analysis based on PC-NTDs surveillance data collected in the previous year. Availability of PC-NTDs case definitions, presence of disease-specific case registers and receipt of feedback on surveillance reports were associated with higher odds of conducting analysis of surveillance data at the facility; [AOR = 2.76, 95% CI: 1.44, 5.31; *p* = 0.002], [AOR = 2.28, 95% CI: 1.08, 4.83; *p* = 0.030] and [AOR = 5.11, 95% CI: 2.13, 12.3; *p* < 0.001] respectively (Table 9). Availability of computers as well as the availability of posters were also associated with higher odds of conducting data analysis at the facility; [AOR: 2.47, 95% CI: 1.18, 5.18; *p* = 0.017] and [AOR = 3.37, 95% CI: 1.52, 7.48; *p* = 0.003) respectively. On the contrary, supervision of surveillance activities was associated with 70% reduction in the odds of conducting data analysis at the facility level [AOR = 0.30, 95% CI: 0.11, 0.81; p = 0.017].

**Table 9:** Factors associated with PC-NTDs surveillance data analysis

		PC-NTDs surveillance data analysis		Unadjusted Estimates		Adjusted Estimates	
Characteristic	N	No, n (%)	Yes, n (%)	OR (95% CI)	***p***-value	OR (95% CI)	***p***-value
Availability of PC-NTDs case definitions							
No	180	41 (61%)	26 (39%)	1.00		1.00	
Yes		32 (28%)	81 (72%)	4.76 (2.69, 8.43)	< 0.001	2.76 (1.44, 5.31)	0.002
*Availability of PC-NTDs case registers*							
No	189	72 (47%)	81 (53%)	1.00		1.00	
Yes		6 (17%)	30 (83%)	4.50 (1.75, 11.60)	0.002	2.28 (1.08, 4.83)	0.030
*Feedback on PC-NTDs surveillance reports*							
No	172	52 (51%)	50 (49%)	1.00		1.00	
Yes		12 (17%)	58 (83%)	5.03 (2.52, 10.0)	< 0.001	5.11 (2.13, 12.3)	< 0.001
Supervision of surveillance activities							
No	189	8 (24%)	26 (76%)	1.00		1.00	
Yes		70 (45%)	85 (55%)	0.37 (0.15, 0.90)	0.029	0.30 (0.11, 0.81)	0.017
Availability of computers							
No	188	57 (51%)	55 (49%)	1.00		1.00	
Yes		21 (28%)	55 (72%)	2.81 (1.40, 5.67)	0.004	2.47 (1.18, 5.18)	0.017
*Availability of surveillance posters*							
No	188	44 (61%)	28 (39%)	1.00		1.00	
Yes		34 (29%)	82 (71%)	3.92 (1.78, 8.66)	0.001	3.37 (1.52, 7.48)	0.003

*N* Number of observations with valid data analysed, *OR* Odds Ratio, *95% CI* 95% Confidence Interval

At the facility level, slightly less than one-third (29%) of respondents reported that their facilities had action thresholds for PC-NTDs. The action thresholds were based mostly (89%) on number of cases reported and to a lesser extent based on percentage increase in number of cases (4%) or rates based on specific variables (4%). Respondents reported initiating mass drug administration and deworming exercises, conducting health education at the community level and putting in place epidemic preparedness measures as the common actions that followed when the number of PC-NTD cases met the set thresholds.

Respondents attributed improved analysis of PC-NTDs surveillance data to provision of proper analytical tools and equipment such as computers with pre-loaded analysis software for effective surveillance data analysis and refining existing reporting tools to accommodate all PC-NTDs. Further recommendations suggested the need for enhanced training and capacity building on analytical skills by involving all health workers across the surveillance levels and conducting sensitisation on the importance of data analysis to inform follow up actions;

*“More training and awareness among health workers on conducting analysis of NTDs surveillance data is needed … through frequent analysis of data we will be able to monitor trends of NTD cases in the region and plan well to control the diseases” –* HFW#027 (West Pokot County).

Additionally, health personnel recommended prioritisation of PC-NTDs in the analysis process and adapting simplified analysis methods to ensure minimal time is spent to complete data analysis;

*“More needs to be done in the region to capture enough NTDs data to warrant analysis … most of the NTDs are not well captured in the data analysis and this needs to be done” –* CDSRC#002 (West Pokot County).

### Feedback

At the community level, 54% (27/50) of respondents reported to receive feedback relating to PC-NTDs from the facility level and 32% indicated that it took more than a week to receive the feedback reports. At the facility level, 37% (70/192) of facilities received feedback on PC-NTDs reports submitted to the next surveillance level in the previous one-year surveillance period. Of this fraction, 39% of the facilities received 1–2 feedback reports while 41% received at least three reports from the higher levels. The ability to meet reporting deadlines and to conduct data analysis at the facility level were associated with increased odds of receiving feedback on surveillance reports; [AOR = 1.80, 95% CI: 1.29, 2.52; *p* = 0.001] and [AOR = 4.55, 95% CI: 2.08, 9.97; *p* < 0.001] respectively (Table 10). Sixty percent (115/192) of facilities did not hold feedback meetings with CHUs and some of the respondents (6%) were not aware of the number of meetings held with CHUs in the previous year;

**Table 10:** Factors associated with feedback reports received from higher levels

		Feedback reports received from higher levels		Unadjusted Estimates		Adjusted Estimates	
Characteristic	N	No, n (%)	Yes, n (%)	OR (95% CI)	***p***-value	OR (95% CI)	***p***-value
Health cadre							
Nurse		72 (65%)	39 (35%)	1.00		1.00	
Clinical Officer	172	22 (56%)	17 (44%)	1.41 (0.64, 3.13)	0.397	1.46 (0.63, 3.38)	0.378
HRMS/LS/PHS		8 (36%)	14 (64%)	3.18 (1.52, 6.65)	0.002	2.41 (1.05, 5.53)	0.037
*Surveillance report submission deadlines met*							
No	172	27 (73%)	10 (27%)	1.00		1.00	
Yes		75 (56%)	60 (44%)	2.18 (1.48, 3.23)	< 0.001	1.80 (1.29, 2.52)	0.001
*Conduct data analysis*							
No	172	52 (81%)	12 (19%)	1.00		1.00	
Yes		50 (46%)	58 (54%)	5.02 (2.49, 10.1)	< 0.001	4.55 (2.08, 9.97)	< 0.001

*N* Number of observations with valid data analysed, *OR* Odds Ratio, *95% CI* 95% Confidence Interval

*“Feedback meetings with members of the community units were mostly based on the common health conditions such as malaria … those diseases affecting the community regularly … but the agenda was not specific to NTDs … I can hardly recall the number of meetings held with the community units in the past year” –* HFW#071 (Kwale County).

Recommendations by respondents for improved feedback regarding PC-NTDs surveillance data suggested the need for regular and timely feedback on reports sent from one level to the other to inform actions at the point of surveillance report generation. In addition, ensuring feedback reports are relevant and applicable to surveillance activities undertaken by the concerned surveillance level;

*“Feedback should be provided promptly and all health staff at the facility should have access to the feedback reports … feedback on submitted reports from our in-charges will enable us gauge our reporting performance and know what actions to take at a facility level” –* HFW#037 (Narok County).

Additional recommendations required adoption of electronic mechanisms or hardcopy written feedback reports to ensure timely feedback is provided to the relevant surveillance levels as opposed to verbal feedback for effective PC-NTDs surveillance and response;

*“We need to improve the feedback mechanisms by adapting electronic methods to ensure timely feedback is provided … using electronic media such as mobile SMSs and emails” –* HFW#142 (Embu County).

### Epidemic preparedness and response

Respondents at the sub county (63%) and county (100%) levels reported the presence of a rapid response team and having adequate outbreak response supplies. Respondents further reported challenges facing effective response to NTDs outbreaks at the sub-national level;

*“At this sub county level we lack a team that has been put together to rapidly respond to outbreaks arising from NTDs … we mostly notify the county level in case of an increased number of reported cases and response is coordinated at that level”* – HFW#127 (Embu County).

*“We mostly rely on the county health management team to coordinate activities for outbreak response because we lack adequate personnel and supplies at the sub county level to efficiently respond to disease outbreaks”* – HFW#065 (Kwale County).

### Supervision

Eighty-one percent (156/192) of facilities received regular supervisory visits from the sub-national levels (Table 11). Of this fraction, slightly more than half (53%) received supervisory visits more than twice in the previous one-year surveillance period. Eighty percent of facilities that received regular supervisory visits had disease surveillance activities reviewed. However, previous supervisory visits focused largely on other common conditions and hardly on PC-NTDs surveillance activities as reported by 53% (83/156) of facility respondents. Higher-level facilities (level 3, 4 and 5) were more likely to receive regular supervisory visits compared to lower level 2 facilities (93% vs. 77%, *p* = 0.008). Respondents at the sub-national level claimed that poor accessibility to remotely located health facilities, unavailability of reliable transport means and inadequate human resource hindered effective supervision of surveillance activities. Respondents (31%, 49/156) further reported that recommendations concerning PC-NTDs surveillance activities were provided during the last supervisory visits with 55% of this fraction reporting that follow-up on previous recommendations were undertaken in the last supervisory visit;

**Table 11:** Support surveillance activities performance relating to PC-NTDs

Support surveillance Activities	Indicators	IDSR Target	Community level (***N*** = 50)	Health facility Level (***N*** = 192)	Sub-county level (***N*** = 19)	County level (***N*** = 10)
		%	% [n/N]	% [n/N]	% [n/N]	% [n/N]
Standards and guidelines	Proportion with IDSR guidelines	80	66 [33/50]	48 [93/192]	100 [19/19]	100 [10/10]
	Proportion with surveillance manuals guiding PC-NTDs surveillance activities	80	50 [25/50]	61 [57/93^a^]	100 [19/19]	100 [10/10]
	Proportion with laboratory standard operating procedures for specimen collection, handling, storage or transportation	80	N/A	46 [88/192]	74 [14/19]	70 [7/10]
Supervision	Proportion regularly supervised	80	60 [30/50]	81 [156/192]	89 [17/19]	90 [9/10]
	Proportion supervised more than twice in the one-year surveillance period	80	73 [22/30]	53 [83/156^b^]	100 [19/19]	100 [10/10]
	Proportion supervised on PC-NTDs surveillance activities	80	33 [10/30^c^]	42 [66/156^c^]	68 [13/19]	60 [6/10]
	Proportion conducting supervision of surveillance activities at the lower levels	80	N/A	41 [78/192]	100 [19/19]	100 [10/10]
Training	Proportion with staff trained on disease surveillance in basic training	80	80 [40/50]	83 [159/192]	100 [19/19]	100 [10/10]
	Proportion with staff who received post-basic training on surveillance activities	80	N/A	20 [39/192]	84 [16/19]	80 [8/10]
	Proportion with staff trained specifically on PC-NTD surveillance activities in post basic training	80	N/A	44 [17/39^d^]	53 [10/19]	50 [5/10]
Resources	Proportion with electricity available	80	N/A	85 [164/192]	100 [19/19]	100 [10/10]
	Proportion with computers available	80	N/A	40 [76/192]	95 [18/19]	100 [10/10]
	Proportion with access to telephone services	80	N/A	85 [164/192]	100 [19/19]	100 [10/10]
	Proportion with access to internet services	80	N/A	24 [47/192]	79 [15/19]	100 [10/10]
	Proportion with PC-NTDs posters available	80	N/A	61 [117/192]	NA	NA

NA – indicator was either not available or unmeasurable at the specific surveillance level

^a,b,c,d^ - denominators are derived from totals of a preceding affirmative outcome

*“During the previous supervisory visit the need for timely reporting of both weekly and monthly surveillance data was overly emphasised … the team followed up recently by reviewing timeliness of previous reports sent over the last couple of months” –* HFW#047 (Kwale County).

Of the facilities (41%, 78/192) that had conducted supervision of surveillance activities at the community level, 41% reported to have conducted supervisory visits more than twice in the previous year. Up to 62% (48/78) of facility respondents reported that PC-NTDs surveillance activities at the community level were reviewed during the last supervisory visit. Of this fraction, 79% reported that written feedback reports were issued to the community levels. Respondents recommended that improved supervision required adequate resource provision in terms of financial and logistical support to facilitate supervisory activities at the community levels. Furthermore, ensuring supervisory teams are well constituted by including an NTD focal person as part of the team;

*“Ensuring there is a designated surveillance focal person always accompanying the supervisory teams to review NTDs surveillance data among other diseases … we require specific staff to be assigned duties for supervision of disease surveillance activities at the lower levels” –* KII#004 (Baringo County).

Further recommendations by respondents alluded to regular supervision of surveillance activities relating to PC-NTDs at the community levels and involvement of community health workers through strengthened and functional CHUs for effective supervision of active case search activities for PC-NTDs. In addition, reinstatement of inactive CHUs to functional status for effective community-based surveillance. Furthermore, respondents recommended the need to train supervisory teams on the conduct of supervisory activities and to put focus on monitoring PC-NTDs surveillance activities during supervision;

*“Important to have on-job trainings and sensitisation of health workers on supervisory activities at the lower levels … this will encourage ownership and should motivate their involvement in surveillance activities” –* HFW#016 (West Pokot County).

### Training

Most facility respondents (83%, 159/192) were trained on disease surveillance during their basic training (Table 11). Of this number, up to 40% admitted that their basic training was sufficient to adequately undertake disease surveillance activities with 67% confirming that the training was applicable to undertaking PC-NTDs surveillance activities at the facility level. Twenty percent (39/192) of facility respondents had received post-basic training on disease surveillance with 64% of this fraction reporting that all elements of disease surveillance and response were covered during the last post-basic training. Up to 44% (17/39) of the respondents reported that the post basic training covered aspects relating to PC-NTDs surveillance. Respondents also reported that surveillance updates were mostly provided through on-the job trainings;

*“Training of health workers on issues regarding disease surveillance in most health facilities was mainly on the basis of on-job training … especially during supervisory visits … formally organised trainings are rarely done” –* SCDSRC#012 (Kilifi County).

Fifty-one percent (20/39) of facility respondents reported that disease surveillance aspects specific to PC-NTDs were not covered in previous post-basic trainings and would be interested in a training focusing on PC-NTDs. Respondents recommended regular training and dissemination of up-to-date information on PC-NTDs for effective surveillance and response to common cases prevalent in the region. In addition, well-formulated training plans and schedules would ensure training covers important aspects relating to PC-NTDs surveillance activities;

*“Need for the sub-county level to ensure on-job trainings and updates especially regarding surveillance of NTDs are frequently provided” –* SCDSRC#007 (Kwale County).

Moreover, respondents identified the need for resource provision through financial incentives for organising training sessions and providing training tools and materials to facilitate training on surveillance activities. Health workers also suggested the need to prioritise training for PC-NTDs surveillance activities. Furthermore, respondents pinpointed the need to involve all the health cadres in training activities to ensure knowledge and awareness on PC-NTDs is cascaded to other health workers. Additionally, need to retain trained staff across surveillance levels for sustained performance of surveillance activities and conduct training needs assessment to determine specific areas of focus regarding PC-NTDs surveillance.

### Resources

Assessment of surveillance resources regarding transport support showed that 13% (24/192) of facilities had motor vehicles available and were fully functional. Up to 67% (16/24) of respondents in these facilities reported that the motor vehicles facilitated PC-NTDs surveillance activities. On the other hand, motor cycles were available in 38% (72/192) of facilities with 92% (66/72) of these facilities having motor cycles that were functional and 92% (61/66) of respondents in these facilities reported that the motor cycles facilitated surveillance activities. Other respondents reported that bicycles were available in 6% (12/192) of the facilities, functional and supported surveillance activities in all the twelve facilities. Electricity supply was available in 85% (164/192) of health facilities. Of these fraction, 98% (161/164) of respondents in these facilities indicated that the electricity was functional with 91% (146/161) reporting that electricity facilitated PC-NTDs surveillance activities. Calculators, computers, printers and photocopiers were available in 69% (133/192), 40% (76/192), 25% (47/192) and 23% (45/192) of the facilities respectively. These equipment were functional in 99% (132/133), 83% (63/76), 87% (41/47) and 91% (41/45) of the facilities and facilitated surveillance activities in 96% (126/132), 83% (52/63), 85% (35/41) and 90% (37/41) of the health facilities respectively. Data analysis software were available in 12% (22/192) of the facilities, functional in 86% (19/22) and facilitated surveillance activities in 63% (12/19) of the facilities. Assessment of communication equipment, internet access and information, education and communication (IEC) materials showed that telephone or mobile phone services were available in 85% (164/192) of the facilities, functional in 98% (160/164) and facilitated surveillance activities in 96% (154/160) of the facilities. Posters, pamphlets and flipcharts for PC-NTDs were available in 61% (117/192), 27% (51/192) and 8% (15/192) of the facilities respectively.

Respondents recommended provision of electronic equipment such as calculators, mobile phones and computers to ease reporting and data analysis at the lower surveillance levels. Additionally, provision of financial incentives to cover for communication and transport costs during surveillance reports submission. Moreover, providing an adequate number of surveillance staff responsible for collection, collation and transmission of reports across the surveillance levels;

*“I wish we had a health staff designated to this facility whose role will be handling of surveillance activities and reports submission … this will allow the rest of the health workers to concentrate on other tasks knowing there is someone responsible for compilation of all reports” –* HFW#122 (Kitui County).

### Satisfaction with PC-NTDs surveillance activities

Logistic regression analysis assessing the predictors of satisfaction with PC-NTDs surveillance and response activities indicated that the facility level, adequacy of forms for reporting PC-NTDs and feedback on submitted PC-NTDs surveillance reports were associated with increased odds of satisfaction with PC-NTDs surveillance and response activities in the endemic region; [AOR = 3.04, 95% CI: 1.77, 5.23; *p* < 0.001], [AOR = 4.25, 95% CI: 2.23, 8.08; p < 0.001] and [AOR = 4.55, 95% CI: 2.30, 9.02; p < 0.001] respectively. Respondents reported that having in place alternative PC-NTDs surveillance strategies within the existing IDSR system would influence their satisfaction with the system;

*“We require a behavioral surveillance system to better understand community dynamics for effective active case finding of neglected diseases … these are diseases* (NTDs) *associated with a lot of stigma, therefore, making it difficult to capture certain cases with a majority of those suffering from example elephantiasis shying away from the public eye”* – KII#009 (Kwale County).

There were eleven a priori identified main themes with up to 62 emerging sub-themes, which were derived based on recommendations to improve PC-NTDs surveillance and response within the existing IDSR system according to health workers’ perspectives (Tables 12 and 13). The numerical value of code groundedness was used to determine the degree of probable evidence for each code [40]. A high degree of code groundedness was defined as those codes (recommendations) that were mentioned fifteen or more times (G ≥ 15) by the research participants under each main theme.

**Table 12:** Recommendations to improve PC-NTDs surveillance core activities

Themes	Codes (Recommendations)	Code groundedness (Quotations)
Case detection	Provide PC-NTDs case definitions	30
	Provide training on application of PC-NTDs case definitions	25
	Simplify PC-NTDs case definitions	15
Case confirmation	Improved laboratory capacity	25
	Sensitisation and training on PC-NTD case confirmation	11
Case registration	Availing PC-NTDs case registers	5
	Improved PC-NTDs case registration	1
Reporting	Prioritising PC-NTDs reporting	79
	Improved and updated PC-NTDs reporting tools	69
	Enhanced training on PC-NTDs data reporting	42
	Adequate provision of reporting forms	20
	Provision of reporting guidelines	5
	Competing tasks for the limited time and resources	4
	Support supervision on reporting	2
	Provision of electronic reporting tools	1
Data analysis	Enhance training on data analysis	87
	Prioritising PC-NTDs surveillance data analysis	24
	Frequent updates on data analysis skills	21
	Involvement of all health cadres in surveillance activities	17
	Provision of data analysis tools and equipment	4
	Providing ample time for data analysis	3
	Provision of guidelines for data analysis	3
	Provide designated staff responsible for data analysis	2
Feedback	Timely feedback on surveillance reports	58
	Regular feedback on surveillance reports	48
	Adopting electronic feedback mechanisms	25
	Enhanced feedback to lower levels	9
	Prioritising PC-NTDs feedback	6
	Training on feedback mechanisms	1
Epidemic preparedness and response	Adequate outbreak response supplies	25
	Well constituted rapid response teams	5
	Training on NTDs epidemic preparedness	4

**Table 13:** Recommendations to improve PC-NTDs surveillance support activities

Themes	Codes (Recommendations)	Code groundedness (Quotations)
Standards and guidelines	Availing PC-NTDs surveillance manuals	20
	Provide guidelines for supervision	18
	Provision of reporting guidelines	5
	Provide guidelines for data analysis	3
Supervision	Regular supervision from higher levels	76
	Prioritising PC-NTDs in supervision agenda	59
	Training and sensitisation on surveillance supervisory activities	29
	Provide properly constituted supervisory teams	22
	Resource provision to facilitate supervisory activities	22
	Community involvement in supervisory activities	12
	Provide focal person for surveillance supervisory activities	7
	Regular supervisory visits to lower levels	7
	Well formulated supervision schedules	4
	Provision of written supervisory reports	3
	Providing tools for conducting supervision	2
Training	Regular sensitisation of health workers on PC-NTDs surveillance	59
	Prioritising PC-NTDs surveillance in training	55
	Involvement of all health workers in PC-NTDs surveillance training	31
	Providing adequate surveillance training materials	28
	Providing frequent updates on PC-NTDs	21
	Retention of trained surveillance staff	10
	Assessment of surveillance training needs for health workers	4
	Proper coordination of surveillance training activities	2
	Adopting modern training techniques using social media platforms	1
Resources	Provide funding to facilitate PC-NTDs surveillance activities	103
	Enhance human resource responsible for surveillance activities	83
	Provision of surveillance tools and equipment	78

## Discussion

The study assessed surveillance core and support function performance based on specific measurable indicators. These indicators were compared to the gold standard IDSR indicator performance target of 80% across all surveillance levels [8]. Study findings identified satisfactory performance of IDSR indicators at the sub-national level as relates to adequate provision of IDSR standard case definitions, capacity to handle PC-NTD specimens, data analysis, trained health personnel and sufficient resources to undertake surveillance activities regarding PC-NTDs. However, indicators at lower surveillance levels hardly met the IDSR performance targets due to surveillance activities implementation challenges relating to unavailability of PC-NTD case definition guidelines and case registers, limited reporting and data analysis, minimal supervision of surveillance activities at lower levels and limited training on surveillance, which together contributed to the sub-optimal performance of PC-NTDs surveillance activities at the sub-national level. These challenges are comparable to findings from studies conducted in other countries in the African region [41–46].

The idea of an integrated disease surveillance and response framework was propositioned by WHO based on the sub-optimal performance of vertical surveillance systems characterised by unstandardised case definitions, numerous reporting forms, limited laboratory capacity and inadequately trained human resource [47]. Optimal functioning and implementation of the IDSR system requires consolidation of all strategic components including effective communication, enhanced laboratory capacity, improved training and sustainable resource provision [44, 48]. A recent assessment of the regional IDSR system implementation status in Africa indicated that most countries had implemented the IDSR framework [49]. About 85% of the countries had initiated IDSR training at the sub-national levels and slightly more than two-thirds had started community-based surveillance in the IDSR context with about 26% having met the desired target of at least 90% implementation coverage in peripheral health facilities [49]. Therefore, improved PC-NTDs surveillance through the IDSR framework is the most pragmatic approach. The IDSR process would enable extensive baseline assessment and gaps identification to inform formulation of PC-NTDs prioritised plans of action for implementation by concerned stakeholders within the health system [50, 51]. Hence, it is critical for countries to recognise hindrances to IDSR implementation by identifying feasible solutions that are tailored for specific countries [52].

In the current study, an assessment of IDSR surveillance functions considering PC-NTDs revealed sub-optimal system performance, which hampered adequate detection and prompt implementation of appropriate public health actions. Focused efforts to improve specific surveillance functions in connection with PC-NTDs are paramount for effective identification of disease foci to inform targeted treatment and halt disease transmission. However, a review of existing literature identified unwavering focus on notifiable diseases in assessing the performance of surveillance systems [43, 44, 53–57]. Therefore, comparable findings to the current study were based mostly on assessment of surveillance functions within the IDSR system with a leaning to notifiable conditions. It is assumable that findings from previous studies assessing the performance of surveillance functions and the ensuing recommendations would be applicable to most diseases under surveillance given the consolidated approach of the IDSR system.

### Core functions

Efficient case detection is dependent on the availability of case definition guidelines and well-trained health personnel on utilising the available case definitions [58]. Availability of surveillance guidelines at the health facility level is the cornerstone of a strengthened surveillance system especially regarding disease notification [42]. In the current study, low utilisation of PC-NTDs case definitions was commonly reported by health facility workers due to unavailability of such guidelines. Similarly elsewhere, health facilities lacked up-to-date IDSR technical guidelines, which bear the standard case definitions [59]. In a study in Madagascar, about two-thirds of healthcare facilities had case definitions available with most health workers being aware of standard case definitions for commonly notifiable conditions such as malaria, measles, diarrheal and respiratory conditions [43]. However, health providers were less aware of case definitions for neglected conditions such as dengue fever [43].

Health providers reported lack of specific case registers for PC-NTDs with the exception of facilities designated as treatment sites. Similarly elsewhere, health facilities lacked dedicated registers to capture specific diseases during investigation [48, 60]. Disease-specific case registers were hardly utilised with most facilities utilising the outpatient registers. However, study participants reported that having disease-specific registers would provide a rumor log for suspected cases detected at the community level, ease assessment of disease burden and facilitate follow-up efforts for the reported PC-NTD cases. Use of general outpatient registers for recording all diseases tends to put emphasis on common conditions given reporting frequency, hence concealing the burden of other neglected conditions [58]. Inadequate registration of PC-NTDs cases according to respondents in the current study was due to lack of specific case registers and increased workload. Similarly, in Zambia, inconsistent case registration resulted from health workers lacking ample time to accurately register cases due to high patient demand and lack of designated data entry clerks [42]. In the present study, peripheral levels lacked the capacity to collect and handle PC-NTD specimens. This was consistent with other findings, where routine case confirmation was limited at the lower health facilities in comparison to disease confirmation efforts at higher-level facilities [56, 58]. Limited laboratory capacities and inadequate number of health workers at the health facility level hinder case detection efforts as depicted in the current study [41]. Health providers identified lack of adequate laboratory guidelines as a barrier to confirmation of PC-NTD cases. Similarly elsewhere, only a few health facilities had laboratory standard operating procedures [14]. Further observations showed that most laboratories lacked essential laboratory reagents, specimen collection and storage equipment and laboratory standard operating procedures for confirming PC-NTDs. Adequate case confirmation is directly dependent on laboratory capacity in terms of having appropriate equipment and availability of skilled laboratory workforce [58].

Some PC-NTDs but not all are reported through the health management information system (HMIS), for instance, data on Soil transmitted helminths, Guinea worm disease and Schistosomiasis are captured at the peripheral level. However, data on Lymphatic Filariasis, Leishmaniasis, Onchocerciasis, Trachoma among other PC-NTDs are not incorporated in the health facility physical registers and the HMIS [24]. These diseases are mostly categorised and reported as “other” conditions. A major challenge hindering PC-NTDs reporting at the lower surveillance levels was lack of adequate reporting tools. Lack of such forms deters efforts to effectively investigate specific diseases and results to loss of vital information to inform adequate response. Reporting forms unavailability and missing guidelines hinder disease notification efforts [45]. Moreover, lack of simplified surveillance tools limits the overall performance of surveillance and response systems [47]. Other previous studies showed that availing the relevant reporting tools improved surveillance performance at the health facility level [32, 52, 61]. Further findings from the current study demonstrated that lack of adequate reporting tools and training on proper reporting demotivated health workers engagement in the surveillance system [56].

Overall, current study findings mainly identified use of paper-based reporting systems especially at the health facility level. Similarly elsewhere, weekly surveillance reports prepared at the health facility level were mostly submitted in paper format [19, 45, 59, 60, 62]. However, use of manual reporting tools reduced data accuracy, hence weakening the surveillance system [60]. Furthermore, our study findings reported cost-burden on health workers linked to reporting forms reproduction. Contrarily, the regional surveillance levels in Ghana took up the responsibility of reproducing and distributing reporting forms, hence reducing the burden to incur reproduction costs on the lower reporting levels [63]. In line with our study findings on irregular reporting methods, inconsistent use of various reporting channels results to inaccuracies in the compiled surveillance reports [32, 41]. Use of varied reporting channels especially at the health facility level hindered effective retrieval of previously reported PC-NTDs data with some of the health workers using paper-based reports while others transmitted reports through phone calls or text messages. In a study assessing the factors associated with weekly surveillance reporting in health facilities in Kenya, the short message service (SMS) was the most preferred channel for reporting [32]. Elsewhere, reporting from health facilities to higher levels was mostly either through phone calls, emails or hand-delivery of hard copy reports [42]. Furthermore, inconsistent use of alternative electronic reports submission mechanisms fosters reluctance among health personnel to submit reports using the conventional paper-based reports [41]. This identifies the need to adapt standard reporting channels to maintain data quality and ease data validation across the surveillance levels. Moreover, increased adoption of the DHIS2 system especially at the lower surveillance levels would improve data accuracy [64]. However, most resource-constrained African nations still face challenges relating to adoption of electronic reporting tools [43].

Findings revealed that surveillance data analysis at the peripheral levels of PC-NTD endemic regions was inadequate. Comparably, routine data analysis was conducted in only a third of the health facilities surveyed from selected WHO African Region countries [47]. Routine surveillance data analysis requirements are dependent on the surveillance level with common forms of data analysis based on person, place and time [42, 65, 66]. Similar to current study findings, several variables including age, sex, place and time were cited as possible data analysis stratifiers. However, challenges of limited data analysis capacities cutting across all surveillance levels have been reported elsewhere [48, 58, 65–68]. Inadequate data analysis limits surveillance system capacity to efficiently detect outbreaks and constrain use of the data for planning and decision-making [58, 63]. Notably, reduced odds of conducting data analysis among regularly supervised facilities in our study could be attributed to limited efforts by health workers to analyse surveillance data due to insufficient data analysis skills. Additionally, respondents claimed lack of adequate time to conduct data analysis due to other competing tasks that were prioritised during the supervisory visits. Further findings discerned that most surveillance levels rarely undertook trend analysis for PC-NTDs except for priority diseases. A notable number of health facilities in the African region showed evidence of plotted trend lines especially for priority conditions [47]. In line with other previous studies, trend analysis was especially conducted for more common conditions such as malaria and diarrheal cases [58, 65]. Routine data analysis is mostly restricted to notifiable conditions and hardly is analysis done on surveillance data collected for other non-priority conditions [59]. Current study findings showed trend analysis was mostly conducted for common conditions but PC-NTDs surveillance data collected by health facilities was hardly sufficient to assess disease trends. Therefore, more emphasis on regularly tracking the number of PC-NTD cases on a short-term or long-term basis in endemic regions could enable adequate planning and inform decisions by stakeholders at the sub-national level. This necessitates reinstitutionalisation of data analysis of all surveillance data to achieve equivalent surveillance and response to all conditions.

Our study findings identified limited feedback to peripheral levels on PC-NTDs surveillance data submitted to the higher surveillance levels. Several other studies have reported low feedback from national or regional levels to the lower surveillance levels [10, 46, 59]. Feedback to peripheral levels of the health system is limited and mostly provided only during health facility supervisory meetings similar to health worker reports from the current study [41]. Contrarily elsewhere, most health facilities regularly provided the peripheral levels with feedback [58]. Other challenges facing feedback from regional to lower surveillance levels relate to unreliable follow up mechanisms to ascertain whether all concerned lower surveillance levels received the feedback [56]. Consistent feedback to the peripheral level motivates community’s involvement in surveillance activities [42, 58]. Additionally, frequent feedback positively influence surveillance system acceptability and willingness of health providers to participate in surveillance activities [69]. Despite reports of low feedback from higher to peripheral surveillance levels, there was relatively higher feedback from the national to regional levels in other NTD endemic countries [58]. Inconsistencies in relaying feedback reports to lower surveillance levels especially to health facilities and the communities have been increasingly reported in other NTD endemic regions similar to the current study findings [42, 58]. Feedback tends to be provided on as-needed basis and hardly on routine-basis. For instance, feedback to health facility levels was mostly prompted by discrepancies with data from the weekly and monthly surveillance reports in Ghana [59]. Motivation of health personnel involvement in surveillance reporting activities can be attributed to provision of regular feedback [52]. Health workers responsible for surveillance activities at the health facility level in Zambia identified timely feedback as being critical to strengthening all aspects of the IDSR system [46]. Similarly, current study findings linked delayed feedback to ineffective PC-NTDs surveillance. In respect to these findings, support supervision across all surveillance levels along provision of prompt feedback would ensure optimal functioning of surveillance activities [10, 60].

Health personnel reported limited outbreak preparedness at the sub-national level regarding adequacy of supplies to respond to probable PC-NTD epidemics in Kenya. Another study showed regional surveillance levels lacked adequate outbreak preparedness supplies due to insufficient budgetary allocation for epidemic emergencies [41]. Poor budgeting and limited logistical support impede adequate epidemic preparedness [41]. Moreover, limited supplies hinder outbreak preparedness and response across the surveillance levels [60]. Hence, underscoring the importance of strengthening outbreak preparedness and response at the peripheral level, which constitutes the first level of responders to suspected epidemics. Findings from the current study indicated that most sub-national levels had well-constituted rapid response teams in place. However, these teams lacked adequate preparedness plans to respond to PC-NTD epidemics. Notably, formulation of standardised disease-specific guidelines for outbreak management and case confirmation would be vital in curbing neglected tropical conditions [63]. Similarly, other countries in Africa had functional outbreak rapid response teams mostly with the capacity to effectively respond to priority diseases but their preparedness to respond to NTDs was obscure [42, 48].

### Support functions

Inaccurate case registration at health facility levels was due to inconsistent use of the standard case definitions similar to our study findings on irregular use of PC-NTDs case definitions [65]. Similarly, inconsistencies in surveillance reports resulted from health personnel relying on their basic training skills for disease diagnoses as opposed to applying the available standard case definitions [65, 66, 70]. Lack of updated case definitions across surveillance system levels was reported in other African countries adopting the IDSR system [60]. Contrarily, health workers tend to be more aware of case definitions for priority conditions as reported in our study findings [47]. Low utilisation of available surveillance guidelines or complete lack of the guidelines at health facility and community levels was reported by health workers in the current study. In comparison to a previous study conducted in Kenya, less than one-tenth of health facilities in an urban setting had IDSR technical guidelines available to health workers [32]. Therefore, unavailability of surveillance guidelines and lack of training on use of the guidelines among health workers responsible for surveillance reports compilation affects overall data quality [43]. Similarly, inadequate sensitisation on the available IDSR guidelines affects health workers attitudes and practices on routine reporting [42].

Supervision of surveillance activities at the peripheral levels of PC-NTD endemic regions in the current study was limited, similar to what has been reported in other studies [51, 59, 71]. Lack of consistent supervisory visits especially at the lower surveillance levels was due to lack of adequate capacity to undertake supervisory activities. Likewise, in Ghana, supervision from district to health facility levels was inconsistently conducted and hardly focused on disease surveillance issues [59]. Similarly, supervisory activities were only initiated in the event of a suspected outbreaks or when need arose in Zambia [42]. Enhanced supervisory support is critical to improving IDSR system performance [52]. In addition, support supervision across all surveillance levels is critical for effective monitoring of surveillance system indicators, improved disease reporting and persuading health workers’ engagement in surveillance activities at the peripheral level [58]. Agreeably, health providers in the present study pointed out that regular supervision motivated their involvement in surveillance activities. Additionally, in line with our findings, consistent supportive supervision to health facility personnel on case detection, surveillance data reporting and analysis is imperative to maintain high-level surveillance performance [47]. On the other hand, current study findings highlighted various challenges to undertaking supervisory activities at the sub-national level. Similar challenges were reported in previous studies, for instance unavailability of funds, unreliable means of transportation to access lower surveillance levels, inadequate number of surveillance personnel and poor attitudes and perceptions towards surveillance activities hindered efforts for support supervision [41, 42]. It is to be expected that inadequate supervisory activities negatively affect surveillance data quality [43], while inconsistent support for supervision across surveillance levels also poses a major challenge to implementation of other surveillance activities [14, 41, 46, 52].

Similar to present study findings, most surveillance staff elsewhere were trained on disease surveillance activities [60]. However, not all health workers especially at the peripheral levels were involved in surveillance training in the current study. The findings somewhat compares to reports of lower surveillance levels facing shortages of trained staff with training mostly focused on staff at the national surveillance levels [60]. Previous study findings elsewhere showed that health personnel lacked adequate training specific to IDSR implementation similar to our findings [42, 46]. Likewise, limited training among health workers on IDSR system implementation has largely been reported in other African countries [59, 72]. Insufficient post-basic training results to health workers’ over-dependence on prior experiences to undertake surveillance activities, hence limiting surveillance data quality [42]. Notably, challenges face sub-national levels in creating adequate capacity to provide IDSR training [63]. Elsewhere, most surveillance personnel had received post-basic training but there were minimal efforts to cascade the knowledge and skills gained to other health facility workers [59]. Surveillance staff reluctance to share information on surveillance activities with other health workers could be attributed to lack of interest shown by health personnel not directly involved in surveillance undertakings [41]. Similar findings from the present study demonstrated that failing to prioritise health worker training and lack of adequate funds limits the capacity of health personnel to effectively undertake surveillance activities [58]. Furthermore, trained staff turnover posed a major challenge to achieving effective surveillance, which was in agreement with our study findings [58]. Trained surveillance staff turnover to a great extent affects operationalisation of the IDSR system and poor utilisation of electronic reporting systems leading to inconsistent reports submission [59, 62]. Inconsistencies amongst health workers regarding application of knowledge gained from their basic training to PC-NTDs surveillance activities could have resulted from differences in specific diseases encountered in the different regions. Nevertheless, health workers in the current study mostly recommended inclusion of specific training on disease surveillance covering NTDs in the pre-service training. Moreover, institutionalisation of training on the IDSR system is the most pragmatic approach for effective and sustainable implementation of the system [19]. Findings also indicated limited formal training for health workers specifically on surveillance activities in PC-NTDs endemic regions. Hence, health workers mostly relied upon on-the job training and regular updates during supervisory visits. Similarly, elsewhere, IDSR training strategies adapted included pre-service training, cascading and on-the job training [47, 60]. The cost burden of facilitating IDSR training requires ministries of health and other development partners to identify other cost-effective and sustainable training strategies [52]. Present study findings identified the need for training health workers on all PC-NTDs surveillance aspects. Elsewhere, health personnel were mostly trained on surveillance supervision strategies and on data management [47]. These demonstrates that disease-specific needs assessment of surveillance aspects that require training is imperative and further elucidates the need to prioritise surveillance training on neglected tropical conditions.

Insubstantial funding in support of surveillance activities has been identified as a major hindrance to optimal functioning of surveillance and response systems [58, 59]. Moreover, inadequate funding of surveillance activities limits preparedness and response to epidemics [44]. Most health workers in our study identified the need for adequate resources to facilitate day-to-day surveillance activities. Similarly, respondents elsewhere reported inadequate financial support for surveillance activities at the health facility level [46]. Surveillance activities are hugely impeded by lack of sufficient financial, human, logistical and infrastructural resources [59]. Similar to reports from the present study, health workers had to bear out-of-pocket costs when submitting surveillance reports due to lack of adequate resource provision to cover for communication expenses [42]. This demonstrated the snowball effect that limited financial resources had on the performance of other surveillance functions and on health workers’ attitudes. Therefore, there is need for increased funding to support disease surveillance activities at the sub-national level [52]. Limited government funds to support implementation of routine surveillance activities require external development partners funding supplementation [48]. Strengthened resource capacity are a requisite measure for sustainable positive gains within surveillance systems [48]. Present study findings identified disproportionate involvement of nurses in surveillance reports preparation and this was because majority of the facilities were second-tier (dispensaries), which were commonly overseen by health workers of the nursing cadres. Furthermore, limited human resource capacity hinders active surveillance activities at the periphery, which compares to current findings showing community levels lacked designated surveillance focal persons [41, 58]. Elsewhere, having a designated surveillance health worker was positively associated with adequate surveillance data reporting [32].

Health providers outlined various logistical challenges limiting surveillance performance in PC-NTDs endemic regions in Kenya. Comparably, elsewhere, weak technical and logistical capacities hindered IDSR system implementation [42]. Consequently, these limited capacities indirectly affected optimal performance of other core surveillance functions [42]. Seemingly, inadequate resource capacity limits ideal functioning and sustainable performance of the surveillance systems [42, 58, 73]. Other resource challenges reported by health workers in the current study related to lack of dependable electricity supply. Previous studies showed that unreliable electricity supply in remote regions impede the use of electronic equipment such as computers [62]. Inadequate communication infrastructure and equipment limits timely transmission of surveillance data [63]. A well-functioning IDSR system necessitates all surveillance levels to reliably communicate and transmit data on a routine-basis or when prompted by an outbreak [63]. Surveillance data reporting at the lower health facility levels in the current study in Kenya was hampered by limited access to reliable communication facilities and ample network connectivity [58, 59]. Effective case notification is hinged on reliable communication mechanisms and enhanced logistical capacity across all surveillance levels [58]. Our study findings indicated unavailability and low utilisation of computers for surveillance-related activities especially at the peripheral levels. Similarly, there was low utilisation of computers for surveillance data management in health facilities in South Sudan and Uganda [14, 46, 60]. Findings elsewhere depicted that the availability of computers did not necessarily mean that they were being put to use to facilitate surveillance activities [63]. This could be attributed to low knowledge levels among health workers on use of computers to perform data analysis. However, access to functional computers would facilitate data analysis and surveillance reports compilation. Information, education and communication resources also played a key role to guide surveillance activities especially at the health facility levels in the current study. Elsewhere, posters displaying IDSR functions were positively associated with adequate surveillance data reporting [32]. However, our findings showed that case definition posters on display at the health facility level were mostly for routinely reported notifiable conditions similar to findings from a previous study in Khartoum State [56].

### Strengths and limitations

The main strength of the current study was the use of a mixed methods survey approach, which gave an in depth understanding of the issues relating to PC-NTDs surveillance within the existing IDSR system based on users’ perceptions. However, there were several limitations to this study, firstly. In the first instance, study participants were required to recount specific surveillance activities conducted in the past, a situation that could have introduced instances of recall bias. This was minimised by limiting the assessment to a one-year surveillance period to ensure accurate recollection of specific surveillance activities. Secondly, the study was based on individual perceptions, which may have influenced some of their responses to what they considered socially desirable. To overcome this limitation, the study participants’ responses were supplemented by direct observations. Thirdly, the study focused on assessing health personnel perspectives at the sub-national, which provides the first contact of health services to NTDs afflicted communities. However, stakeholder perspectives at the national level are crucial in the policymaking process and further investigations need to be undertaken in subsequent studies. Fourth, logistical challenges relating to inaccessible study sites, which were characterised by very poor terrain, harsh climatic conditions and personal security concerns, were mitigated through use of telephone interviews. Lastly, there were limited number of studies assessing PC-NTDs surveillance activities to make sufficient comparisons with the current study findings. However, this study provides a comprehensive overview of critical recommendations to address key challenges facing PC-NTDs surveillance within the IDSR system.

## Conclusion

The sub-national level is the crux of IDSR implementation with the capacity to provide adequate response to public health conditions. Over reliance on donor-initiated PC-NTDs control programmes in endemic regions leads to reluctant control efforts by the sub-national health systems. Principally, strengthened health information systems are a product of quality data generated through effective and well-functioning disease surveillance and response systems. Surveillance core and support functions regarding common disease conditions seem to perform optimally and have been documented extensively in Kenya [11, 32]. However, findings from the current study indicate that a great deal of effort is required to achieve effective surveillance and response to PC-NTDs. Findings revealed implementation of the IDSR system in PC-NTD endemic regions in Kenya hardly prioritised PC-NTDs reporting with low utilisation of case definitions and limited application of surveillance guidelines, lacked adequate feedback mechanisms and support supervision, was short of trained surveillance staff and faced challenges of limited financial and logistical resources. These outcomes were in part due to lack of proper PC-NTDs case-based surveillance systems across the sub-national surveillance levels with the current IDSR system focusing mainly on notifiable conditions. Findings depicted the symbiotic association between surveillance core and support functions to achieve optimal surveillance system performance. Therefore, reviewing existing sub-national policies with a keen focus on strengthening specific surveillance functions based on recommendations derived from health personnel perceptions would improve the overall surveillance and response efforts for PC-NTDs. Recommendations specific to improving PC-NTDs core surveillance activities at the sub-national level were linked to provision of simplified case definitions, improved laboratory capacity, provision of PC-NTDs reporting tools, timely and regular feedback on surveillance reports, adopting electronic feedback mechanisms, improved data analysis skills, provision of adequate outbreak response supplies. Furthermore, recommendations to improve surveillance support functions pointed to availing surveillance guidelines for supervision, having properly constituted supervisory teams, regular supervision from higher levels, providing frequent updates on PC-NTDs, regular sensitisation and involvement of all health workers in surveillance activities and enhanced training on PC-NTDs case definitions, data reporting, data analysis and supervisory activities. Moreover, enhancing human resource capacity, provision of surveillance tools, equipment and training materials, provision of adequate funding to facilitate surveillance activities and provision of reliable transport means.

The sub-national levels are compelled to strengthen PC-NTDs case-based surveillance to ensure adequate case confirmation of suspected cases and early detection of probable outbreaks as a critical measure to control and eliminate the diseases. The study further recommends periodic and objective assessment of the surveillance system focusing on PC-NTDs. The sub-national level of the healthcare system provides an adequate platform for operational activities of disease surveillance and response systems as opposed to the national levels that provide a much more strategic platform for decision-making [74]. Therefore, formulating strategic plans for disease surveillance at the sub-national will ensure long-term sustainable achievements. Moreover, decentralising key surveillance functions to the peripheral levels would be critical to improving commitment among health personnel by fully engaging them in surveillance activities right from the beginning. Essentially, there is need to raise profiles of surveillance units at the sub-national level and compel policy makers to prioritise surveillance activities for domestic revenue allocations.

## Supplementary Information

**Additional file 1.**

## Data Availability

The datasets generated and/or analysed to assess the surveillance system attributes are not publicly available due to the need to keep the identities of respondents confidential as they granted consent to be enrolled in the study on the basis of remaining anonymous, but are available from the corresponding author on reasonable request.

## Abbreviations

AOR: Adjusted Odds Ratio
ARNTD: African Research Network for Neglected Tropical Diseases
CDC: Centers for Disease Control and Prevention
CDSRCs: County Disease Surveillance and Response Coordinators
CHEWs: Community Health Extension Workers
CHIROs: County Health Information and Records Officers
CHUs: Community Health Units
CI: Confidence Interval
COR-NTD: Coalition for Operational Research on Neglected Tropical Diseases
DHIS2: Demographic Health Information System II
ESPEN: Expanded Special Project for Elimination of Neglected Tropical Diseases
GWD: Guinea Worm Disease
HFWs: Healthcare Facility Workers
HIS: Health Information Systems
HRMS: Health Records Management Staff
IDS: Integrated Disease Surveillance
IDSR: Integrated Diseases Surveillance and Response
IEC: Information, Education and Communication
KII: Key Informant Interview
KMHFL: Kenya Master Health Facility List
LS: Laboratory Staff
MOH: Ministry of Health
NTDs: Neglected Tropical Diseases
OR: Odds Ratio
PC-NTDs: Preventive Chemotherapy-targeted Neglected Tropical Diseases
PHS: Public Health Staff
SARAM: Service Availability and Readiness Mapping
SCDSRCs: Sub-County Disease Surveillance and Response Coordinators
SDGs: Sustainable Development Goals
SMSs: Short Message Services
UK Aid: UK Aid from the British People
USA: United States of America
USAID: United States Agency for International Development
VPD: Vaccine Preventable Diseases
WHO: World Health Organization
WHO-AFRO: World Health Organization Regional Office for Africa

## References

[1] Block MG, Akosa A, Chowdhury M. Health systems research and infectious diseases of poverty: from the margins to the mainstream. Global Rep Res Infect Dis Pov. 2012; https://www.who.int/tdr/capacity/global_report/2012/authors/en.

[2] Fisher G, Pappas G, Limb M (1996). Prospects, problems, and prerequisites for national health examination surveys in developing countries. Soc Sci Med.

[3] Gyapong JO, Gyapong M, Yellu N, Anakwah K, Amofah G, Bockarie M (2010). Integration of control of neglected tropical diseases into health-care systems: challenges and opportunities. Lancet.

[4] Thacker SB, Berkelman RL (1988). Public health surveillance in the United States. Epidemiol Rev.

[5] WHO (2006). Communicable disease surveillance and response systems: guide to monitoring and evaluating.

[6] WHO. Integrated disease surveillance in the African Region: a regional strategy for communicable diseases (1999-2003). World Health Organization Regional Office for Africa 1998. https://apps.who.int/iris/handle/10665/1749.

[7] WHO (2000). Assessment protocol for national disease surveillance and epidemic preparedness and response.

[8] WHO. Technical guidelines for integrated disease surveillance and response in the African region: World Health Organization Regional Office for Africa; 2010. p. 1–398. https://www.afro.who.int/publications/technical-guidelines-integrated-disease-surveillance-and-response-african-region-third

[9] Nsubuga P, Eseko N, Tadesse W, Ndayimirije N, Stella C, McNabb S (2002). Structure and performance of infectious disease surveillance and response, United Republic of Tanzania, 1998. Bull World Health Organ.

[10] Xiong W, Lv J, Li L (2010). A survey of core and support activities of communicable disease surveillance systems at operating-level CDCs in China. BMC Public Health.

[11] MOH-Kenya (2012). Technical Guidelines for Integrated Disease Surveillance and Response in Kenya.

[12] United Nations (2013). Sixty-ninth session of the united National General Assembly: draft outcome document of the United Nations summit for the adoption of the post-2015 development agenda.

[13] Fitzpatrick C, Engels D (2016). Leaving no one behind: a neglected tropical disease indicator and tracers for the sustainable development goals. Int Health.

[14] Wamala JF, Okot C, Makumbi I, Natseri N, Kisakye A, Nanyunja M (2010). Assessment of core capacities for the international health regulations (IHR [2005]) - Uganda 2009. BMC Public Health.

[15] Hollingsworth TD, Langley I, Nokes DJ, Macpherson EE, McGivern G, Adams ER (2015). Infectious disease and health systems modelling for local decision making to control neglected tropical diseases. BMC Proc.

[16] WHO (2017). Integrating neglected tropical diseases into global health and development: fourth WHO report on neglected tropical diseases.

[17] WHO (2018). Controlling and eliminating neglected tropical diseases [fact sheet].

[18] Tambo E, Ai L, Zhou X, Chen JH, Hu W, Bergquist R (2014). Surveillance-response systems: the key to elimination of tropical diseases. Infect Dis Poverty.

[19] Phalkey RK, Yamamoto S, Awate P, Marx M (2015). Challenges with the implementation of an integrated disease surveillance and response (IDSR) system: systematic review of the lessons learned. Health Policy Plan.

[20] Sahal N, Reintjes R, Aro AR (2009). Communicable diseases surveillance lessons learned from developed and developing countries: literature review. Scand J Public Health.

[21] MOH-Kenya (2013). Report on the Baseline Assessment of Capacity for Monitoring and Evaluation.

[22] WHO (2001). Protocol for the assessment of national communicable disease surveillance and response systems: guidelines for assessment teams.

[23] CDC. Updated guidelines for evaluating public health surveillance systems. MMWR Recomm Rep. 2001;50(1–35). Available from: https://www.cdc.gov/mmwr/preview/mmwrhtml/rr5013a1.htm. Accessed 11 Feb 2020. 18634202

[24] MOH-Kenya (2016). The 2nd Kenya National Strategic Plan for Control of Neglected Tropical Diseases 2016-2020.

[25] MOH-Kenya (2019). Kenya National Breaking Transmission Strategy for Soil-Transmitted Helminthiasis, Schistosomiasis, Lymphatic Filariasis and Trachoma (2019-2023).

[26] WHO. The Expanded Special Project for Elimination of Neglected Tropical Diseases (ESPEN) 2017. Annu Rep:2018 https://apps.who.int/iris/handle/10665/272344?show=full.

[27] Mapsland. Detailed location map of Kenya in Africa. Available from: https://www.mapsland.com/africa/kenya/detailed-location-map-of-kenya-in-africa. Accessed 13 July 2020.

[28] Slidemodel. Editable Kenya PowerPoint Map. Available from:https://slidemodel.com/templates/editable-kenya-powerpoint-map/. Accessed 22 July 2020.

[29] Kenya National Bureau of Statistics (2019). Kenya population and housing census volume I: population by county and sub-county.

[30] Sightsavers (2019). Kenya landscape analysis for neglected tropical diseases (NTDs), WASH and behaviour change.

[31] Oyore J, Mwitari J, Ouma J, Ndung’u E (2010). Evaluation report of the community health strategy implementation in Kenya.

[32] Mwatondo AJ, Ng'ang'a Z, Maina C, Makayotto L, Mwangi M, Njeru I (2016). Factors associated with adequate weekly reporting for disease surveillance data among health facilities in Nairobi County, Kenya, 2013. Pan Afr Med J.

[33] Curry LA, Krumholz HM, O’Cathain A, Clark VLP, Cherlin E, Bradley EH (2013). Mixed methods in biomedical and health services research. Circ Cardiovasc Qual Outcomes.

[34] MOH-Kenya. Kenya Master Health Facility List. Kenya: Ministry of Health. Available from: http://kmhfl.health.go.ke. Accessed on 15 Jan 2020.

[35] Government of Kenya (2014). Kenya Service Availability and Readiness Assessment Mapping (SARAM).

[36] Polkinghorne DE (2005). Language and meaning: data collection in qualitative research. J Couns Psychol.

[37] Patton MQ. Qualitative research & evaluation methods: integrating theory and practice: Sage publications; 2014.

[38] Rubin H, Rubin I (2005). Qualitative interviewing.

[39] Friese S (2019). Qualitative data analysis with ATLAS. ti. Sage publications.

[40] Muhr T (2003). ATLAS. ti Visual qualitative data analysis (Version 5).

[41] Adokiya MN, Awoonor-Williams JK, Barau IY, Beiersmann C, Mueller O (2015). Evaluation of the integrated disease surveillance and response system for infectious diseases control in northern Ghana. BMC Public Health.

[42] Mandyata CB, Olowski LK, Mutale W (2017). Challenges of implementing the integrated disease surveillance and response strategy in Zambia: a health worker perspective. BMC Public Health.

[43] Randriamiarana R, Raminosoa G, Vonjitsara N, Randrianasolo R, Rasamoelina H, Razafimandimby H (2018). Evaluation of the reinforced integrated disease surveillance and response strategy using short message service data transmission in two southern regions of Madagascar, 2014-15. BMC Health Serv Res.

[44] Issah K, Nartey K, Amoah R, Bachan EG, Aleeba J, Yeetey E (2015). Assessment of the usefulness of integrated disease surveillance and response on suspected ebola cases in the Brong Ahafo region, Ghana. Infect Dis Poverty.

[45] Mairosi N, Tshuma C, Juru T, Gombe N, Shambira G, Tshimanga M (2017). Evaluation of notifiable disease surveillance system in Centenary District, Zimbabwe, 2016. O J Epi.

[46] Haakonde T, Lingenda G, Munsanje F, Chishimba K (2018). Assessment of factors affecting the implementation of the integrated disease surveillance and response in public health care facilities-the case of Rufunsa District. Zambia Divers Equal Health Care.

[47] Sow I, Alemu W, Nanyunja M, Duale S, Perry HN, Gaturuku P (2010). Trained district health personnel and the performance of integrated disease surveillance in the WHO African region. East Afr J Public Health.

[48] Lukwago L, Nanyunja M, Ndayimirije N, Wamala J, Malimbo M, Mbabazi W (2013). The implementation of integrated disease surveillance and response in Uganda: a review of progress and challenges between 2001 and 2007. Health Policy Plan.

[49] Fall IS, Rajatonirina S, Yahaya AA, Zabulon Y, Nsubuga P, Nanyunja M (2019). Integrated disease surveillance and response (IDSR) strategy: current status, challenges and perspectives for the future in Africa. BMJ Glob Health.

[50] Perry HN, McDonnell SM, Alemu W, Nsubuga P, Chungong S, Otten MW (2007). Planning an integrated disease surveillance and response system: a matrix of skills and activities. BMC Med.

[51] Nsubuga P, Brown WG, Groseclose SL, Ahadzie L, Talisuna AO, Mmbuji P (2010). Implementing integrated disease surveillance and response: four African countries' experience, 1998-2005. Glob Public Health.

[52] Masiira B, Nakiire L, Kihembo C, Katushabe E, Natseri N, Nabukenya I (2019). Evaluation of integrated disease surveillance and response (IDSR) core and support functions after the revitalisation of IDSR in Uganda from 2012 to 2016. BMC Public Health.

[53] Maponga BA, Chirundu D, Shambira G, Gombe NT, Tshimanga M, Bangure D (2014). Evaluation of the Notifiable diseases surveillance system in Sanyati district, Zimbabwe, 2010-2011. Pan Afr Med J.

[54] Tsitsi JP, Nomagugu N, Gombe NT, Tshimanga M, Donewell B, Mungati M (2015). Evaluation of the Notifiable diseases surveillance system in Beitbridge District, Zimbabwe 2015. O J Epi.

[55] Ngwa MC, Liang S, Mbam LM, Mouhaman A, Teboh A, Brekmo K (2016). Cholera public health surveillance in the Republic of Cameroon-opportunities and challenges. Pan Afr Med J.

[56] Baghdadi I (2016). Assessment of core and support functions of case-based surveillance of meningitis in hospitals in Khartoum state in 2015. East Mediterr Health J.

[57] Lakew GA, Wassie E, Ademe A, Fenta A, Wube S, Werede M (2017). Status of surveillance and routine immunization performances in Amhara region, Ethiopia: findings from in-depth peer review. Pan Afr Med J.

[58] Phalkey RK, Shukla S, Shardul S, Ashtekar N, Valsa S, Awate P (2013). Assessment of the core and support functions of the integrated disease surveillance system in Maharashtra, India. BMC Public Health.

[59] Adokiya MN, Awoonor-Williams JK, Beiersmann C, Muller O (2015). The integrated disease surveillance and response system in northern Ghana: challenges to the core and support functions. BMC Health Serv Res.

[60] Sahal N, Reintjes R, Eltayeb E, Aro AR (2012). Assessment of core activities and supportive functions for the communicable diseases surveillance system in Khartoum state, Sudan, 2005-2007. East Mediterr Health J.

[61] Lar LA, Tagurum YO, Uzochukwu B, Zoakah AI, Afolaranmi TO (2015). Challenges of integrated disease surveillance response reporting among healthcare personnel in Mangu, plateau state, Nigeria. J Public Health and Epidemiol.

[62] Kiberu VM, Matovu JK, Makumbi F, Kyozira C, Mukooyo E, Wanyenze RK (2014). Strengthening district-based health reporting through the district health management information software system: the Ugandan experience. BMC Med Inform Decis Mak.

[63] Franco LM, Setzer J, Banke K (2006). Improving performance of IDSR at district and facility levels: experiences in Tanzania and Ghana in making IDSR operational.

[64] Adokiya MN, Awoonor-Williams J, Beiersmann C, Mueller O (2016). Evaluation of the reporting completeness and timeliness of the integrated disease surveillance and response system in northern Ghana. Ghana Med J.

[65] Rumisha SF, Mboera LE, Senkoro KP, Gueye D, Mmbuji PK (2007). Monitoring and evaluation of integrated disease surveillance and response in selected districts in Tanzania. Tanzan Health Res Bull.

[66] Mghamba JM, Mboera LEG, Krekamoo W, Senkoro KP, Rumisha SF, Shayo E (2004). Challenges of implementing an integrated disease surveillance and response strategy using the current health management information system in Tanzania. Tanzan J Health Res.

[67] Abubakar A, Sambo M, Idris S, Sabitu K, Nguku P (2013). Assessment of integrated disease surveillance and response strategy implementation in selected local government areas of Kaduna state. Ann Niger Med.

[68] Gueye D, Senkoro KP, Rumisha SF (2005). Baseline monitoring and evaluation of integrated disease surveillance and response in Tanzania.

[69] Benson FG, Musekiwa A, Blumberg L, Rispel LC (2016). Survey of the perceptions of key stakeholders on the attributes of the south African Notifiable diseases surveillance system. BMC Public Health.

[70] Franco LM, Fields R, Mmbuji PK, Posner S, Mboera LE (2003). Situation analysis of infectious disease surveillance in two districts in Tanzania 2002.

[71] Nsubuga P, Nwanyanwu O, Nkengasong JN, Mukanga D, Trostle M (2010). Strengthening public health surveillance and response using the health systems strengthening agenda in developing countries. BMC Public Health.

[72] Nnebue CC, Onwasigwe CN, Adogu PO, Onyeonoro UU (2012). Awareness and knowledge of disease surveillance and notification by health-care workers and availability of facility records in Anambra state, Nigeria. Niger Med J.

[73] Somda ZC, Meltzer MI, Perry HN, Messonnier NE, Abdulmumini U, Mebrahtu G (2009). Cost analysis of an integrated disease surveillance and response system: case of Burkina Faso, Eritrea, and Mali. Cost Eff Resour Alloc.

[74] AbouZahr C, Boerma T (2005). Health information systems: the foundations of public health. Bull World Health Organ.

