# Supplementary material for: Assessment of surveillance core and support functions regarding neglected tropical diseases in Kenya
Source: BMC Public Health. 2021 Jan 15;21:142. doi: 10.1186/s12889-021-10185-1 (PMC7809780; doi:10.1186/s12889-021-10185-1)
Supplement: Supplementary file 1 — Additional file 1. [file 12889_2021_10185_MOESM1_ESM.pdf]

COMMUNITY LEVEL QUESTIONNAIRE (DODOSO LA KIWANGO CHA JAMII)

**GENERAL INFORMATION**<br>***(Habari kwa ujumla)***	**Please mark with a tick (√) in the appropriate box**<br>***(Tafadhali weka alama ya tiki (√) katika sehemu inayofaa zaidi)***	
Demographic Characteristics<br>*(Demografia ya mhojiwa)*	Age *(Umri)*	☐ 18-30 ☐ 31-40<br>☐ 41-50 ☐ >50
	Sex *(Jinsia)*	☐ Male *(Kiume)*<br>☐ Female *(Kike)*
Area of residence<br>*(Makazi ya mhojiwa)*	County *(Kaunti)*: …………………………………<br><br>Sub-County *(Kaunti ndogo)*: ……………………..	
Community Health Unit & linked health facility<br>*(Kitengo cha afya cha jamii na kituo cha afya husika)*	……………………………………. linked to *(kiungo cha)*<br><br>………………………………………………..	
What is your current designation?<br>*(Wajibu wako kikazi sasa hivi ni ipi?)*	…………………………………………………	
Number of years worked in your current designation<br>*(Miaka yako kikazi katika uajibu huu?)*	☐ Less than 1 year *(Chini ya mwaka 1)*<br>☐ 1-2 years *(Kati ya mwaka 1-2)*<br>☐ 2-3 years *(Kati ya miaka 2-3)*<br>☐ 3-5 years *(Kati ya miaka 3-5)*<br>☐ More than 5years *(Zaidi ya miaka 5)*	
What is your highest level of education?<br>*(Kiwango chako cha juu zaidi katika masomo?)*	☐ PhD *(Shahada ya uzamifu)*<br>☐ Masters *(Shahada ya uzamili)*<br>☐ Degree *(Shahada)*<br>☐ Diploma *(Stashahada)*<br>☐ Certificate *(Astashahada)*<br>☐ No education *(Hamna masomo)*	
**DISEASE SURVEILLANCE *(UFUATILIAJI WA MAGONJWA)***		
**Q1**. What do you understand by community-based disease surveillance?<br>*(Je unaelewa vipi ufuatiliaji wa magonjwa katika kiwango cha jamii?)*		
**Q2 (a).** Are you involved in disease surveillance activities in this region?<br>*(Je unahusika na shughuli za ufuatiliaji wa magonjwa katika mkoa huu?*	Yes *(Ndio)* ☐ No *(La)* ☐	

**Q2 (b).** If YES, what is your role in disease surveillance in this region?<br>*(Kama NDIO, jukumu lako ni lipi kwa ufuatiliaji wa magonjwa katika mkoa huu?)*	
**Q3 (a).** Are you familiar with the term Neglected Tropical Diseases?<br>*(Je unafahamu magonjwa ya kitropiki yaliyo telekezwa?*	Yes *(Ndio)* ☐ No *(La)* ☐
**Q3 (b).** If YES, which are the preventive chemotherapy targeted neglected tropical diseases (PC-NTDs) common in this area?<br>*(Kama NDIO, je magonjwa haya ya kitropiki yaliyo telekezwa ambazo ni za kawaida katika mkoa huu ni zipi?)*	☐ Lymphatic Filariasis *(Matende)*<br>☐ Soil Transmitted Helminths *(Minyoo)*<br>☐ Trachoma *(Trakoma)*<br>☐ Schistosomiasis *(Kichocho)*<br>☐ Others *(Magonjwa zinginezo)*: …………………..
**Q4 (a).** Have you come across any of the PC-NTDs mentioned above in this region in the past one year?<br>*(Je, kwa mwaka uliyopita ulikabiliana na magonjwa haya ya kitropiki yaliyo telekezwa ambayo ulitaja hapo awali?)*	Yes *(Ndio)* ☐ No *(La)* ☐ DK *(Sijui)* ☐ N/A *(Si Husika)* ☐
**Q4 (b).** If YES, what action did you take?<br>*(Kama NDIO, je ulichukua hatua ipi?)*	
**Q4 (c).** Did you report the PC-NTD/s?<br>*(Je, uliripoti magonjwa haya ya kitropiki yaliyo telekezwa?)*	Yes *(Ndio)* ☐ No *(La)* ☐ DK *(Sijui)* ☐ N/A *(Si Husika)* ☐
**Q4 (d).** If YES, to whom did you report the disease/s to?<br>*(Kama NDIO, je uliripoti magonjwa haya kwa nani?)*	☐ Health Facility *(Kituo cha afya)*<br>☐ Fellow Colleague *(Mfanyakazi mwenza)*<br>☐ Others *(Wengineyo)*: ……………………………
**Q4 (e).** Which PC-NTD/s have you reported to the health facility in the past one year?<br>*(Je, ni magonjwa yapi ya kitropiki yaliyo telekezwa ulizoripoti katika kituo cha afya kwa mwaka uliyopita?*	☐ Lymphatic Filariasis *(Matende)*<br>☐ Soil Transmitted Helminths *(Minyoo)*<br>☐ Trachoma *(Trakoma)*<br>☐ Schistosomiasis *(Kichocho)*<br>☐ Others *(Magonjwa zinginezo)*: …………………...
**Q5 (a).** Are there forms for reporting diseases to the health facility?<br>*(Je kuna fomu maalum za kuripoti magonjwa kwa madhumuni ya kuzielekeza katika kituo cha afya?)*	Yes *(Ndio)* ☐ No *(La)* ☐ DK *(Sijui)* ☐ N/A *(Si Husika)* ☐
**Q5 (b).** If YES, are the reporting forms readily available?<br>*(Kama NDIO, je fomu hizi ya kuripoti zinapatikana kwa urahisi?)*	Yes *(Ndio)* ☐ No *(La)* ☐ DK *(Sijui)* ☐ N/A *(Si Husika)* ☐
**Q5 (c).** If NO, why do you say so?<br>*(Kama LA, tafadhali nipe sababu zako?)*	
**Q6.** Have you lacked disease reporting forms at any given time in the past 6 months?	Yes *(Ndio)* ☐ No *(La)* ☐ DK *(Sijui)* ☐ N/A *(Si Husika)* ☐

(Je, mushawahi kumbwa na ukosefu wa fomu hizi za kuripoti magonjwa kwa wakati wowote kwa muda wa miezi sita zilizopita?)	
**Q7 (a).** Do the reporting forms have provision for reporting PC-NTDs?<br>*(Je, fomu hizi za kuripoti zipo na utoaji wa kuripotia magonjwa haya ya kitropiki yaliyo telekezwa?*	Yes *(Ndio)* ☐ No *(La)* ☐ DK *(Sijui)* ☐ N/A *(Si Husika)* ☐
**Q7 (b).** If YES, is the provision sufficient for reporting PC-NTDs?<br>*(Kama NDIO, je utoaji huu ni wakutosha katika kuripotia magonjwa haya ya kitropiki yaliyo telekezwa?)*	Yes *(Ndio)* ☐ No *(La)* ☐ DK *(Sijui)* ☐ N/A *(Si Husika)* ☐
**Q7 (c).** If NO, why is this so?<br>*(Kama LA, tafadhali nipe sababu zako?)*	
**Q8 (a).** Are the disease reporting forms easy to complete?<br>*(Je, ni rahisi kukamilisha fomu hizi za kuripotia magonjwa?)*	Yes *(Ndio)* ☐ No *(La)* ☐ DK *(Sijui)* ☐ N/A *(Si Husika)* ☐
**Q8 (b).** If NO, why do you say so?<br>*(Kama LA, tafadhali nipe sababu zako?)*	
**Q9.** What channel do you use to report surveillance data for PC-NTDs to the health facility?<br>*(Je, unatumia njia zipi kuripoti habari za ufuatiliaji wa magonjwa haya ya kitropiki yaliyo telekezwa kwa madhumuni ya kuzielekeza katika kituo cha afya?)*	☐ In Person *(Katika mtu/Mwenyewe)*<br>☐ Phone Call *(Kupiga simu)*<br>☐ Mobile SMS *(Kutuma ujumbe fupi kwa kutumia simu ya rununu)*<br>☐ Email *(Kutumia barua pepe)*<br>☐ Other *(Njia zinginezo)*: ………………………..<br>☐ N/A *(Si husika)*
**Q10.** How can PC-NTDs reporting be improved?<br>*(Je, kuripoti kwa magonjwa haya ya kitropiki yaliyo telekezwa inaweza kuboreshwa kwa njia zipi?)*	
**Q11 (a).** Do you receive feedback of the reported PC-NTD/s from the health facility?<br>*(Je, unapokea maoni kutoka kwa kituo cha afya ambayo unaripoti magonjwa haya ya kitropiki yaliyo telekezwa?)*	Yes *(Ndio)* ☐ No *(La)* ☐ DK *(Sijui)* ☐ N/A *(Si Husika)* ☐
**Q11 (b).** If YES, after how long do you receive feedback?<br>*(Kama NDIO, je inachukua muda gani kupokea maoni haya?)*	☐ 1-3days<br>☐ 2-7days<br>☐ >1week
**Q12.** How is feedback conveyed from the health facility?<br>*(Je, maoni kutoka kwa kituo cha afya inapokewa kwa njia ipi?)*	☐ In Person *(Katika mtu/Mwenyewe)*<br>☐ Phone Call *(Kupiga simu)*<br>☐ Mobile SMS *(Kutuma ujumbe fupi kwa kutumia simu ya rununu)*<br>☐ Email *(Kutuma barua pepe)*<br>☐ Other *(Njia zinginezo)*: …………………………

	☐ N/A *(Si husika)*
**Q13.** How can feedback from the health facility be improved?<br>*(Je maoni kutoka kwa kituo cha afya inaweza kuboreshwa kwa njia zipi?)*	
**CASE DETECTION, REGISTRATION & GUIDELINES *(UGUNDUZI, USAJILI NA MIONGOZO)***	**NB: Continue to this section if participant answered YES to Q4 (c) *(endelea ikiwa mhojiwa alijibu NDIO kwa swali Q4(c)*)**
**Q14.** How did you identify the suspected PC-NTD case you reported to the health facility?<br>*(Je, ni vipi ulibaini tuhumu la kesi la ugonjwa huu wa kitropiki iliyo telekezwa ambayo uliripoti katika kituo cha afya?)*	☐ Observed symptoms *(Kwa kutazama dalili za ugonjwa)*<br>☐ Screening *(Kwa uchunguzi)*<br>☐ Rumors *(Kwa njia za fununu)*<br>☐ Others *(Zinginezo): ……………………………...*<br>☐ N/A *(Si husika)*
**Q15 (a).** Have you been issued with guidelines for identifying PC-NTDs?<br>*(Je, kuna mitoleo ya kuongoza utambulishaji wa magonjwa haya ya kitropiki yaliyo telekezwa?)*	Yes *(Ndio)* ☐ No *(La)* ☐ DK *(Sijui)* ☐ N/A *(Si Husika)* ☐
**Q15 (b).** If YES, who issued you with the guidelines?<br>*(Kama NDIO, ni nani anatoa miongozo hii?)*	☐ Health facility *(Kituo cha Afya)*<br>☐ NGOs *(Shirika zisizo za serikali)*<br>☐ Other *(Zinginezo)*: ………………………………<br>☐ N/A *(Si husika)*
**Q16 (a).** Have you been trained on use of the guidelines?<br>*(Je, umepewa mafunzo ya kutumia miongozo hii?)*	Yes *(Ndio)* ☐ No *(La)* ☐
**Q16 (b).** If YES, how long ago were you trained?<br>*(Kama NDIO, mafunzo ilikuwa kwa muda gani?)*	☐ <1 year ago (Zaidi ya mwaka moja iliopita)<br>☐ 2-4 years ago (Kati ya mbili na nne iliopita)<br>☐ >5years ago (Zaidi ya miaka tano iliopita)<br>☐ N/A *(Si husika)*
**Q16 (c).** Who facilitated the training?<br>*(Je, ni nani aliwezesha mafunzo haya?)*	☐ Health facility *(Kituo cha Afya)*<br>☐ NGOs *(Shirika zisizo za serikali)*<br>☐ Other *(Zinginezo)*: ………………………………<br>☐ N/A *(Si husika)*
**Q17 (a).** Do you find the guideline useful in identifying PC-NTD/s?<br>*(Je, miongozo hii ni ya muhumi katika kutambua magonjwa haya ya kitropiki yaliyo telekezwa?)*	Yes *(Ndio)* ☐ No *(La)* ☐ DK *(Sijui)* ☐ N/A *(Si Husika)* ☐
**Q17 (b).** If NO, why do you say so?<br>*(Kama LA, tafadhali nipe sababu zako?)*	
**Q18 (a).** Are you aware of the term case definitions?<br>*(Je, unafahamu miongozo za kueleza kesi za magonjwa?)*	Yes *(Ndio)* ☐ No *(La)* ☐
**Q18 (b).** If YES, are PC-NTDs case definitions provided in the guidelines?	Yes *(Ndio)* ☐ No *(La)* ☐ DK *(Sijui)* ☐ N/A *(Si Husika)* ☐

(Kama NDIO, je miongozo hii inaeleza kesi za magonjwa ya kitropiki yaliyo telekezwa?)	
**Q18 (c).** If YES, is it easy to apply the PC-NTDs case definitions provided in the guidelines?<br>*(Kama NDIO, je ni rahisi kutumia miongozo hii inayoeleza kesi za magonjwa ya kitropiki yaliyo telekezwa?)*	Yes *(Ndio)* ☐ No *(La)* ☐ DK *(Sijui)* ☐ N/A *(Si Husika)* ☐
**Q19 (a).** Did you register any information of the last PC-NTD case identified?<br>*(Je, ulirekodi habari zozote kuhusu kesi ya mwisho iliyotambulika ya ugonjwa wa kitropiki iliyo telekezwa?)*	Yes *(Ndio)* ☐ No *(La)* ☐ DK *(Sijui)* ☐ N/A *(Si Husika)* ☐
**Q19 (b).** If YES, what information did you register about the PC-NTD case identified?<br>*(Kama NDIO, je ni habari zipi ulizorekodi za kesi hizi za magonjwa ya kitropiki yaliyo telekezwa?)*	☐ Name of disease *(Jina la ugonjwa)*<br>☐ Symptoms observed *(Dalili ulizo tazama)*<br>☐ Location of patient *(Makazi ya mgonjwa)*<br>☐ Personal details of patient *(Maelezo kuhusu mgonjwa)*<br>☐ Dates and Time *(Tarehe na saa)*<br>☐ Other details *(Maelezo zinginezo)*:……………… …………<br>☐ N/A *(Si husika)*
**CASE CONFIRMATION *(KUTHIBITISHA KESI ZA MAGONJWA)***	
**Q20 (a).** Have you received any training on specimen collection?<br>*(Je, umepokea mafunzo ya ukusanyaji wa sampuli za magonjwa?)*	Yes *(Ndio)* ☐ No *(La)* ☐
**Q20 (b).** If YES, how long ago was the training?<br>*(Kama NDIO, mafunzo hii ilikuwa kwa muda gani?)*	☐ <1 year ago *(Chini ya mwaka moja uliopita)*<br>☐ 2-4 years ago *(Kati ya miaka mbili na miaka nne)*<br>☐ >5years ago *(Zaidi ya miaka tano)*<br>☐ N/A *(Si husika)*
**Q21 (a).** Did you ever collect specimen/s from a suspected PC-NTD case you identified in the previous year?<br>*(Je, umehusika na ukusanyaji wa sampuli za magonjwa za kitropiki yaliyo telekezwa ulizo tambua kwa mwaka uliopita?)*	Yes *(Ndio)* ☐ No *(La)* ☐
**Q21 (b).** If YES, what kind of specimen/s did you collect?<br>*(Kama NDIO, je ni sampuli zipi haswa ulizokusanya?)*	☐ Blood *(Damu)*<br>☐ Urine *(Mkojo)*<br>☐ Stool *(Kinyesi)*<br>☐ Others *(Zinginezo)*: ……………………………...<br>☐ N/A *(Si husika)*
**Q21 (c).** Where did you send the specimen/s?<br>*(Je, ni wapi haswa ulituma sampuli hizi?)*	☐ Public health facility *(Katika kituo cha afya)*<br>☐ Private laboratory *(Maabara ya kibinafsi)*<br>☐ Other *(Zinginezo)*: ……………………………….

	☐ N/A *(Si husika)*
**Q21 (d).** After how long did you get feedback on the specimen sent of the last suspected PC-NTD case you reported in the past year?<br>*(Je, kwa mwaka uliopita ulipata ripoti ya maoni kuhusu sampuli za magonjwa ya kitropiki yaliyo telekezwa baada ya muda gani?*	☐ 1-3days *(Kati ya siku moja hadi tatu)*<br>☐ 4-7days *(Kati ya siku nne hadi saba)*<br>☐ >1week *(Baada ya zaidi ya wiki moja)*<br>☐ N/A *(Si husika)*
**DATA ANALYSIS *(UCHANGANUZI WA DATA)***	
**Q22 (a).** Do you analyse data collected from the identified cases of PC-NTDs?<br>*(Je, huwa unachanganua habari za magonjwa ya kitropiki yaliyo telekezwa ulizo kusanya?)*	Yes *(Ndio)* ☐ No *(La)* ☐
**Q22 (b).** If YES, how do you analyse the PC-NTDs data collected?<br>*(Kama NDIO, je unachanganua habari hizi ulizo kusanya kwa njia zipi?)*	☐ By age *(Kwa Umri)* ☐ By place *(Kwa Makao)*<br>☐ By sex *(Kwa Jinsia)* ☐ By time (*Kwa Saa)*<br>☐ Other *(Njia zinginezo):* ………………………….<br>☐ N/A *(Si husika)*
**OPPORTUNITIES FOR IMPROVEMENT *(NAFASI ZA UBORESHAJI WA UFUATILIAJI WA MAGONJWA)***	
**Q23 (a).** Are you satisfied with the community based-surveillance systems for PC-NTDs in this region as it is?<br>*(Je, umeridhishwa na shughuli za ufuatiliaji wa magonjwa ya kitropiki yaliyo telekezwa katika kiwango cha jamii wa mkoa huu?)*	Yes *(Ndio)* ☐ No *(La)* ☐
**Q23 (b).** If NO, why do you say so?<br>*(Kama LA, tafadhali nipe sababu zako?)*	
**Q24.** What can be done to improve community based surveillance for PC-NTDs in the region?<br>*(Je, shughuli hizi za ufuatiliaji wa magonjwa ya kitropiki yaliyo telekezwa katika kiwango cha jamii zinaweza kuboreshwa kwa njia zipi kwa mkoa huu?)*	

HEALTH FACILITY LEVEL QUESTIONNAIRE *(DODOSO LA KIWANGO CHA KITUO CHA AFYA)*

**GENERAL INFORMATION**<br>***(Habari kwa ujumla)***	**Please mark with a tick (√) in the appropriate box**<br>***(Tafadhali weka alama ya tiki (√) katika sehemu inayofaa zaidi)***	
Health facility name<br>*(Jina la kituo cha afya)*	………………………………………………………….	
Demographic Characteristics<br>*(Demografia ya mhojiwa)*	Age *(Umri)*	☐ 18-30 ☐ 31-40<br>☐ 41-50 ☐ >50
	Sex *(Jinsia)*	Male *(Kiume)* ☐ Female *(Kike)* ☐
**Q1 (a).** What is your health cadre?<br>*(Wajibu wa mfanyakazi wa afya)*	☐ Medical Practitioner *(Matibabu daktari)*<br>☐ Nurse *(Muuguzi)*<br>☐ Public Health Staff *(Mfanyakazi wa afya ya umma)*<br>☐ Laboratory Staff *(Mfanyakazi wa maabara)*<br>☐ Health Records Management Staff *(Mfanyakazi wa kumbukumbu za afya)*<br>☐ Other Cadre *(Uajibu zinginezo):*……………………….	
**Q1 (b).** Number of years worked in health cadre<br>*(Miaka yako kikazi katika uajibu huu?)*	☐ Less than a year *(Chini ya mwaka 1)*<br>☐ 1-2 years *(Kati ya mwaka 1-2)*<br>☐ 2-3 years *(Kati ya miaka 2-3)*<br>☐ 3-5 years *(Kati ya miaka 3-5)*<br>☐ More than 5years *(Zaidi ya miaka 5)*	
**Q2.** What is your highest level of education?<br>*(Kiwango chako cha juu zaidi katika masomo?)*	☐ PhD *(Shahada ya uzamifu)*<br>☐ Masters *(Shahada ya uzamili)*<br>☐ Degree *(Shahada)*<br>☐ Diploma *(Stashahada)*<br>☐ Certificate *(Astashahada)*	
**DISEASE SURVEILLANCE (*UFUATILIAJI WA MAGONJWA)***		
**Q3 (a).** What do you understand by the term disease surveillance?<br>*(Je unaelewa vipi ufuatiliaji wa magonjwa?)*		
**Q3 (b).** What do you understand by the term integrated disease surveillance and response (IDSR) system?<br>*(Je unaelewa vipi mfumo wa IDSR?)*		
**Q4 (a).** What do you understand by health facility based disease surveillance?<br>*(Je unaelewa vipi ufuatiliaji wa magonjwa katika kituo cha afya?)*		

**Q4 (b).** In your current cadre, how many years have you been involved in health facility based disease surveillance?<br>*(Je, katika wajibu wako umehusika na shughli za ufuatiliaji wa magonjwa katika kituo hiki cha afya kwa miaka ngapi)*	☐ Less than a year *(Chini ya mwaka 1)*<br>☐ 2-3 years *(Kati ya miaka 2-3)*<br>☐ 3-5 years *(Kati ya miaka 3-5)*<br>☐ More than 5years *(Zaidi ya miaka 5)*
**Q5.** What do you understand by the term neglected tropical diseases?<br>*(Je, unaelewa ki vipi istilahi ya magonjwa ya kitropiki yaliyo telekezwa?)*	
**Q6 (a).** Are you aware of any neglected tropical diseases in this area?<br>*(Je unafahamu magonjwa haya ya kitropiki yaliyo telekezwa katika mkoa huu?)*	☐ Yes *(Ndio)* ☐ No *(La)* ☐ DK *(Sijui)*
**Q6 (b).** If YES, which are the neglected tropical diseases (PC-NTDs) common in this area?<br>*(Kama NDIO, je magonjwa haya ya kitropiki yaliyo telekezwa ambazo ni za kawaida katika mkoa huu ni zipi?)*	☐ Lymphatic Filariasis *(Matende)*<br>☐ Soil Transmitted Helminths *(Minyoo)*<br>☐ Trachoma *(Trakoma)*<br>☐ Schistosomiasis *(Kichocho)*<br>☐ Others *(Magonjwa zinginezo)*: ……………………….....<br>☐ Don't Know *(Sijui)*
**Q6 (c).** Which of the diseases mentioned above were commonly diagnosed in this facility in the previous year?<br>*(Je ni magonjwa yapi ya kitropiki yaliyo telekezwa ulizo taja zilizofanyiwa utambuzi kwa mwaka uliopita katika kituo hiki cha afya?)*	☐ Lymphatic Filariasis *(Matende)*<br>☐ Soil Transmitted Helminths *(Minyoo)*<br>☐ Trachoma *(Trakoma)*<br>☐ Schistosomiasis *(Kichocho)*<br>☐ Others *(Magonjwa zinginezo)*: ……………………….....<br>☐ Don't Know *(Sijui)*
**Q7 (a).** Is there a health facility based disease surveillance system present in this facility?<br>*(Je kuna mfumo maalum ya ufuatiliaji wa magonjwa katika kituo hiki cha afya?)*	☐ Yes *(Ndio)* ☐ No *(La)* ☐ DK *(Sijui)*<br>☐ N/A *(Si husika)*
**Q7 (b).** If YES, are any of the above mentioned PC-NTDs reported through this system?<br>*(Kama NDIO, je magonjwa ya kitropiki yaliyo telekezwa zinazoripotiwa kupitia mfumo huu?)*	Yes *(Ndio)* ☐ No *(La)* ☐ DK *(Sijui)* ☐ N/A
**Q7 (c).** If YES, specifically which PC-NTDs are reported through this system?<br>*(Kama NDIO, je ni magonjwa yapi ya kitropiki yaliyo telekezwa zinazoripotiwa kupitia mfumo huu?)*	☐ Lymphatic Filariasis *(Matende)*<br>☐ Soil Transmitted Helminths *(Minyoo)*<br>☐ Trachoma *(Trakoma)*<br>☐ Schistosomiasis *(Kichocho)*<br>☐ Others *(Magonjwa zinginezo)*: ……………………….....<br>☐ Don't Know *(Sijui)*<br>☐ N/A *(Si Husika)*
**Q8 (a).** In your own opinion, is it important to have PC-NTDs surveillance systems in this facility?<br>*(Kwa maoni yako binafsi, je mfumo huu wa ufuatiliaji wa magonjwa haya ya kitropiki yaliyo telekezwa ni ya muhimu katika kituo hiki cha afya?)*	☐ Yes *(Ndio)* ☐ No *(La)* ☐ DK *(Sijui)* ☐ N/A
**Q8 (b).** If NO, why do you say so?	

(Kama LA, tafadhali nipe sababu zako?)	
**CASE DETECTION, REGISTRATION & GUIDELINES** ***(UTAMBUZI, USAJILI NA MIONGOZO ZA KESI ZA MAGONJWA)***	
**Q9 (a).** Are standard case definitions as provided in the IDSR guideline available in this facility? *(Je, miongozo za kutambua kesi za magonjwa zinapatikana katika kituo hiki cha afya?)*	☐ Yes *(Ndio)* ☐ No *(La)* ☐ DK *(Sijui)* ☐ N/A
**Q9 (b).** If NO, why is this so? *(Kama LA, sababu ni zipi?)*	
**Q9 (c).** If YES, are the standard case definitions being utilized in this facility? *(Kama NDIO, je miongozo hizi za utambuzi wa kesi za magonjwa zinatumika katika kituo hiki cha afya?)*	☐ Yes *(Ndio)* ☐ No *(La)* ☐ DK *(Sijui)* ☐ N/A
**Q9 (d).** If NO, why do you think this is so? *(Kama LA, sababu ni zipi?)*	
**Q10 (a).** Are the standard case definitions for PC-NTDs provided in the IDSR guideline clear and easy to use? *(Je, miongozo za kutambua kesi za magonjwa ya kitropiki yaliyo telekezwa ni wazi na rahisi kutumika?)*	☐ Yes *(Ndio)* ☐ No *(La)* ☐ DK *(Sijui)* ☐ N/A
**Q10 (b).** If NO, why do you say so? *(Kama LA, sababu ni zipi?)*	
**Q11 (a).** Do you have case registers specific for registration of PC-NTDs in this facility? *(Je, kuna orodha maalum ya kuandikisha kesi za magonjwa ya kitropiki yaliyo telekezwa katika kituo hiki cha afya?)*	☐ Yes *(Ndio)* ☐ No *(La)* ☐ DK *(Sijui)* ☐ N/A
**Q11 (b).** If NO, why is this so? *(Kama LA, sababu ni zipi?)*	
**Q12 (a).** Is a manual for disease surveillance present in this facility? *(Je kuna mwongozo maalum ya ufuatiliaji wa magonjwa katika kituo hiki cha afya?)*	☐ Yes *(Ndio)* ☐ No *(La)* ☐ DK *(Sijui)* ☐ N/A
**Q12 (b).** If YES, is the disease surveillance manual in use in this facility of a recent version? *(Kama NDIO, je mwongozo huu wa ufuatiliaji wa magonjwa ni wa hivi karibuni?)*	☐ Yes *(Ndio)* ☐ No *(La)* ☐ DK *(Sijui)* ☐ N/A
**Q12 (c).** Is the manual useful in guiding disease surveillance activities in this facility? *(Je, mwongozo huu ni muhimu katika kuongoza shughli za ufuatiliaji wa magonjwa katika kituo hiki cha afya?)*	☐ Yes *(Ndio)* ☐ No *(La)* ☐ DK *(Sijui)* ☐ N/A
**Q12 (d).** If NO, why is this so? *(Kama LA, sababu ni zipi?)*	
**Q12 (e).** Does this manual specifically guide PC-NTDs surveillance activities in this facility? *(Je, mwongozo huu inaongoza shughli za ufuatiliaji wa magonjwa ya kitropiki yaliyo telekezwa kwa kituo hiki?)*	☐ Yes *(Ndio)* ☐ No *(La)* ☐ DK *(Sijui)* ☐ N/A
**Q12 (f).** If NO, why is this so? *(Kama LA, sababu ni zipi?)*	
**CASE CONFIRMATION** ***(UTHIBITISHO WA KESI ZA MAGONJWA)***	
**Q13 (a).** Is there a functional laboratory in this facility? *(Je kuna maabara inayotumika katika kituo hiki cha afya?)*	☐ Yes *(Ndio)* ☐ No *(La)* ☐ DK *(Sijui)* ☐ N/A

**Q13 (b).** If YES, is the laboratory adequately equipped to confirm cases of PC-NTDs*?*<br>*(Kama NDIO, je maabara hii inavifaa vya kutosha vya kudhibitisha magonjwa ya kitropiki yaliyo telekezwa?)*	☐ Yes *(Ndio)* ☐ No *(La)* ☐ DK *(Sijui)* ☐ N/A
**Q14.** Is this facility able to collect and store specimens for PC-NTDs in this facility?<br>*(Je kituo hiki cha afya kina uwezo wa kukusanya na kuhifadhi sampuli za magonjwa ya kitropiki yaliyo telekezwa?)*	☐ Yes *(Ndio)* ☐ No *(La)* ☐ DK *(Sijui)* ☐ N/A
**Q15.** Does this facility have the capacity to transport specimens for PC-NTDs to a higher level laboratory?<br>*(Je kituo hiki cha afya kina uwezo wa kusafirisha sampuli za magonjwa ya kitropiki yaliyo telekezwa kwa maabara za juu zaidi?)*	☐ Yes *(Ndio)* ☐ No *(La)* ☐ DK *(Sijui)* ☐ N/A
**Q16 (a).** Does this facility have guidelines for specimen collection, handling, storage and transportation to the next level?<br>(*Je kituo hiki cha afya kina miongozo za ukusanyaji, utunzaji, uhifaji na usafirishaji wa sampuli za magonjwa kuelekezwa katika maabara za juu zaidi?)*	☐ Yes *(Ndio)* ☐ No *(La)* ☐ DK *(Sijui)* ☐ N/A
**Q16 (b).** If NO, why is this so?<br>*(Kama LA, sababu ni zipi?)*	
**Q16 (c).** Has this facility sent specimens for any of the PC-NTDs mentioned earlier to a higher laboratory in the past one year?<br>*(Je kwa mwaka uliopita kituo hiki cha afya kimeweza kutuma sampuli za magonjwa ya kitropiki yaliyo telekezwa ulizo taja hapo awali kwa maabara za juu zaidi?)*	☐ Yes *(Ndio)* ☐ No *(La)* ☐ DK *(Sijui)* ☐ N/A
**Q17 (a).** If YES, did this facility receive reports for PC-NTDs specimens sent to the higher-level laboratory in the past one year?<br>*(Je kwa mwaka uliopita kituo hiki cha afya ilipokea ripoti za sampuli za magonjwa ya kitropiki yaliyo telekezwa yaliyotumwa kwa maabara za juu zaidi?)*	☐ Yes *(Ndio)* ☐ No *(La)* ☐ DK *(Sijui)* ☐ N/A
**Q17 (b).** If NO, why is this so?<br>*(Kama LA, sababu ni zipi?)*	
**Q17 (c).** If YES, were the reports received from the higher level laboratory reliable for diagnosis of PC-NTDs?<br>*(Kama NDIO, je ripoti hizi kutoka maabara za juu zilikuwa za kuaminika katika utambuzi wa magonjwa haya ya kitropiki yaliyo telekezwa?)*	☐ Yes *(Ndio)* ☐ No *(La)* ☐ DK *(Sijui)* ☐ N/A
**Q17 (d).** If NO, why is this so?<br>(*Kama LA, sababu ni zipi?)*	
**Q17 (e).** Were the reports received from the higher level laboratory complete?<br>*(Je ripoti hizi kutoka maabara za juu zilikuwa kamili?)*	☐ Yes *(Ndio)* ☐ No *(La)* ☐ DK *(Sijui)* ☐ N/A
**Q17 (f).** If NO, why do you think this was so?<br>(*Kama LA, sababu ni zipi?)*	
**SURVEILLANCE DATA REPORTING *(KURIPOTI KWA HABARI ZA UFUATILIAJI WA MAGONJWA)***	
**Q18 (a).** Are disease surveillance reporting forms always available in this facility?	☐ Yes *(Ndio)* ☐ No *(La)* ☐ DK *(Sijui)* ☐ N/A

(Je fomu za kuripoti ufuatiliaji wa magonjwa zinapatikana kila wakati katika kituo hiki cha afya?)	
**Q18 (b).** If NO, why is this so?<br>(*Kama LA, sababu ni zipi?)*	
**Q19 (a).** Have you lacked disease surveillance reporting forms at any given time over the past 6 months?<br>*(Je, mushawahi kumbwa na ukosefu wa fomu hizi za kuripoti magonjwa kwa wakati wowote kwa muda wa miezi sita zilizopita?)*	☐ Yes *(Ndio)* ☐ No *(La)* ☐ DK *(Sijui)* ☐ N/A
**Q19 (b).** If YES, why was this so?<br>(*Kama NDIO, sababu ni zipi?)*	
**Q20 (a).** Do the surveillance forms have provision for reporting PC-NTDs?<br>*(Je fomu hizi zinautoaji wa kuripoti magonjwa haya ya kitropiki yaliyo telekezwa)?*	☐ Yes *(Ndio)* ☐ No *(La)* ☐ DK *(Sijui)* ☐ N/A
**Q20 (b).** If NO, why do you think this is the case?<br>(*Kama LA, sababu ni zipi?)*	
**Q21 (a).** Is provision in the forms sufficient for reporting PC-NTDs?<br>*(Je utoaji huu ni wa kutosha katika kuripotia magonjwa haya ya kitropiki yaliyo telekezwa?*	☐ Yes *(Ndio)* ☐ No *(La)* ☐ DK *(Sijui)* ☐ N/A
**Q21 (b).** If NO, why do you say so?<br>(*Kama LA, sababu ni zipi?)*	
**Q22 (a).** Are the reporting forms easy to complete?<br>*(Je, ni rahisi kukamilisha fomu hizi za kuripotia magonjwa?)*	☐ Yes *(Ndio)* ☐ No *(La)* ☐ DK *(Sijui)* ☐ N/A
**Q22 (b).** If NO, why is this so?<br>(*Kama LA, sababu ni zipi?)*	
**Q23 (a).** Are there deadlines for sending PC-NTDs surveillance reports from this facility?<br>(*Je kuna tarehe za mwisho za kutuma ripoti za ufuatiliaji wa magonjwa ya kitropiki yaliyo telekezwa kutoka kwa kituo hiki cha afya?)*	☐ Yes *(Ndio)* ☐ No *(La)* ☐ DK *(Sijui)* ☐ N/A
**Q23 (b).** What is the deadline for submitting PC-NTDs surveillance reports to the higher level?<br>*(Je tarehe za mwisho za kutuma ripoti za ufuatiliaji wa magonjwa haya ya kitropiki yaliyo telekezwa kuelekea kiwango ya juu ni zipi?)*	Q23(b)(i)…..……….............of every week *(wa kila wiki)*<br>Q23(b)(ii)………………..of every month *(wa kila mwezi)*<br>☐ DK *(Sijui)*
**Q23 (c).** Do you often meet the deadline for submission of PC-NTDs surveillance reports?<br>*(Je, munafaulu kwa mara nyingi kutuma ripoti hizi za ufuatiliaji wa magonjwa ya kitropiki yaliyo telekezwa kabla ya tarehe za mwisho kufika?)*	☐ Yes *(Ndio)* ☐ No *(La)* ☐ DK *(Sijui)* ☐ N/A
**Q23 (d).** If NO, why is this so?<br>(*Kama LA, sababu ni zipi?)*	
**Q24.** How long does it take to prepare the PC-NTDs surveillance reports to be sent to the higher level?<br>*(Je inachukua muda upi kutayarisha ripoti hizi za ufuatiliaji wa magonjwa ya kitropiki yaliyo telekezwa kabla kutumwa katika kiwango cha juu?)*	☐ A couple of minutes *(madakika kadhaa)*<br>☐ A couple of hours *(masaa kadhaa)*<br>☐ A couple of days *(siku kadhaa)*<br>☐ A couple of weeks *(wiki kadhaa)*

	☐ A couple of months *(miezi kadhaa)*<br>☐ N/A
**Q25 (a).** Who prepares PC-NTDs surveillance reports in this facility?<br>*(Nani anahusika na utayarishaji wa ripoti hizi za ufuatiliaji wa magonjwa ya kitropiki yaliyo telekezwa katika kituo hiki cha afya?)*	☐ Medical/Clinical Officer In-Charge *(Daktari mkuu)*<br>☐ Nurse In-Charge *(Muuguzi mkuu)*<br>☐ Public Health Staff *(Mfanyakazi wa afya ya umma)*<br>☐ Laboratory Staff *(Mfanyakazi wa maabara)*<br>☐ Health Records Management Staff *(Mfanyakazi wa kumbukumbu za afya)*<br>☐ Other Cadre *(Uajibu zinginezo)*:……………………….<br>☐ N/A
**Q25 (b).** Is he/she trained in preparing disease surveillance reports inclusive of PC-NTDs?<br>*(Je anaye husika na utayarishaji wa ripoti hizi za ufuatiliaji wa magonjwa ikiwemo ya kitropiki yaliyo telekezwa amepata mafunzo maalum?)*	☐ Yes *(Ndio)* ☐ No *(La)* ☐ DK *(Sijui)* ☐ N/A
**Q26 (a).** Is "zero-reporting" undertaken for instances where there were no PC-NTDs reportable cases at any given month?<br>(*Je ripoti za sufuri zinawasilishwa kwa mfano pasipo magonjwa haya ya kitropiki yaliyo telekezwa kwa kila mwezi?)*	☐ Yes *(Ndio)* ☐ No *(La)* ☐ DK *(Sijui)* ☐ N/A
**Q26 (b).** If NO, why is this not done?<br>(*Kama LA, sababu za kutotuma ripoti hizi ni zipi?)*	
**Q27 (a).** Through which channel/s do you report surveillance data for PC-NTDs to the next level?<br>*(Je, unatumia njia zipi kuripoti habari za ufuatiliaji wa magonjwa haya ya kitropiki yaliyo telekezwa kuzielekeza katika kiwango cha juu?)*	☐ In Person *(Katika mtu/Mwenyewe)*<br>☐ Phone Call *(Kupiga simu)*<br>☐ Mobile SMS *(Kutuma ujumbe fupi kwa kutumia simu ya rununu)*<br>☐ Email *(Kutumia barua pepe)*<br>☐ eIDSR/DHIS *(Njia za kielektroniki)*<br>☐ Other *(Njia zinginezo)*: ……………………………..<br>☐ N/A
**Q27 (b).** Are there challenges experienced reporting PC-NTDs surveillance data through this channel/s?<br>*(Je kuna changamoto zozote zinazo kumba njia hizi za kuripoti habari za ufuatiliaji wa magonjwa ya kitropiki yaliyo telekezwa ulizotaja?)*	☐ Yes *(Ndio)* ☐ No *(La)* ☐ DK *(Sijui)* ☐ N/A
**Q27 (c).** If YES, what are some of these challenges?<br>*(Kama NDIO, tafadhali taja changamoto hizi)*	
**Q28.** In your own opinion, how can reporting of PC-NTDs surveillance data be improved in this facility?<br>*(Kwa maoni yako binafsi, kuripoti kwa habari za ufuatiliaji wa magonjwa ya kitropiki yaliyo telekezwa zinaweza kuimarishwa kwa njia zipi katika kituo hiki cha afya?)*	
**DATA ANALYSIS *(UCHANGANUZI WA HABARI ZA UFUATILIAJI WA MAGONJWA)***	

**Q29 (a).** Do you analyse surveillance data for PC-NTDs in this facility?<br>*(Je, kwa kawaida huwa munachanganua habari za ufuatiliaji wa magonjwa ya kitropiki yaliyo telekezwa katika kituo hiki cha afya?*	☐ Yes *(Ndio)* ☐ No *(La)* ☐ DK *(Sijui)* ☐ N/A
**Q29 (b).** If YES, how is the data analysed?<br>*(Kama NDIO, munachanganua habari hizi mulizo kusanya kwa njia zipi?)*	☐ By age *(Kwa umri)*<br>☐ By sex *(Kwa jinsia)*<br>☐ By place *(Kwa makao)*<br>☐ By time *(Kwa saa)*<br>☐ Others *(Kwa njia zinginezo):……………………………*<br>☐ N/A
**Q30 (a).** Do you perform trend analysis of PC-NTD cases in this facility?<br>*(Je, munachanganua mienendo ya magonjwa haya ya kitropiki yaliyo telekezwa katika kituo hiki cha afya?)*	☐ Yes *(Ndio)* ☐ No *(La)* ☐ DK *(Sijui)* ☐ N/A
**Q30 (b).** If NO, why is this not done?<br>(*Kama LA, sababu za kutofanya hivyo ni zipi?)*	
**Q31 (a).** Do you have an action threshold for the PC-NTDs reported in this facility?<br>*(Je, munavizingiti vya matendo katika kituo hiki cha afya inayofuata kuripotiwa kwa magonjwa haya ya kitropiki yaliyo telekezwa?)*	☐ Yes *(Ndio)* ☐ No *(La)* ☐ DK *(Sijui)* ☐ N/A
**Q31 (b).** If YES, what was the action threshold for PC-NTDs reported in the past year?<br>*(Kama NDIO, je vizingiti hivi vya matendo ambavyo vilifuata kuripotiwa kwa magonjwa ya kitropiki yaliyo telekezwa kwa mwaka uliyopita ni yapi?)*	☐ Number of cases *(Nambari ya kesi za magonjwa)*<br>☐ Percentage increase in number of cases *(Ongezeko wa asilimia fulani ya kesi za magonjwa)*<br>☐ Rates *(Kulingana na viwango maalum)*<br>☐ N/A
**Q31 (c).** What action followed the last PC-NTD reported that met the action threshold in the last year?<br>*(Je, kwa mwaka uliyopita ni matendo yapi yalifuatilia kuripotiwa kwa magonjwa ya kitropiki yaliyo telekezwa ambazo zilitimia vizingiti vya matendo yaliyowekwa?)*	…………………………………………………………<br>☐ DK *(Sijui)* ☐ N/A *(Si husika)*
**Q32 (a).** Are you satisfied with the analysis done for PC-NTDs surveillance data in this facility?<br>*(Je, umeridhishwa na uchanganuzi wa habari za ufuatiliaji wa magonjwa haya ya kitropiki yaliyo telekezwa katika kituo hiki cha afya?)*	☐ Yes *(Ndio)* ☐ No *(La)* ☐ DK *(Sijui)* ☐ N/A
**Q32 (b).** If NO, why is this so?<br>(*Kama LA, sababu ni zipi?)*	
**FEEDBACK *(MAONI)***	
**Q33 (a).** Is feedback received on PC-NTDs reports sent to the higher level?<br>*(Je, munapokea maoni kufuatilia ripoti za ufuatiliaji wa magonjwa ya kitropiki yaliyo telekezwa yaliyotumwa kuelekea kiwango cha juu?)*	☐ Yes *(Ndio)* ☐ No *(La)* ☐ DK *(Sijui)* ☐ N/A
**Q33 (b).** If YES, how many feedback reports for PC-NTDs has this health facility received from the higher level in the past one year?<br>*(Kama NDIO, je kwa mwaka uliopita mulipokea ripoti ngapi za maoni katika kituo hiki cha afya kufuatilia*	☐ 1-2 *(Moja au Miwili)*<br>☐ 3 or more *(Tatu au Zaidi)*<br>☐ Don't Know *(Sijui)*<br>☐ N/A *(Si Husika)*

kutuma ripoti za ufuatiliaji wa magonjwa ya kitropiki yaliyo telekezwa kwa kiwango cha juu?)	
**Q34.** How many meetings regarding PC-NTDs has this health facility conducted with those representing the Community Health Units in the past one year?<br>*(Je, kwa mwaka uliopita mumeandaa mikutano ngapi katika kituo hiki cha afya na baadhi ya wanachama wa kitengo cha afya cha jamii zinazohusu magonjwa haya ya kitropiki yaliyo telekezwa?)*	☐ None *(Hamna)*<br>☐ 1-2 *(Mara moja au miwili)*<br>☐ 3 or more (*Mara tatu au zaidi)*<br>☐ Don't Know *(Sijui)*<br>☐ N/A *(Si Husika)*
**Q35.** How can feedback coming from the higher level be improved?<br>*(Je, ripoti za maoni kutoka viwango vya juu vinaweza kuimarishwa na wasimamizi kwa njia zipi?)*	
**SUPERVISION *(USIMAMIZI)***	
**Q36 (a).** Do you receive regular supervisory visits from the higher levels in this facility?<br>*(Je munapokea usimamizi wa mara kwa mara katika kituo hiki cha afya kutoka kwa maafisa wa viwango vya juu?)*	☐ Yes *(Ndio)* ☐ No *(La)* ☐ DK *(Sijui)* ☐ N/A
**Q36 (b).** If NO, why do think this is so?<br>(*Kama LA, sababu ni zipi?)*	
**Q36 (c).** If YES, how often have you received supervisory visits from the higher level in the past one year?<br>*(Kama NDIO, je kwa mwaka uliopita mulipokea ziara za usimamizi mara ngapi kutoka kwa viwango vya juu?)*	☐ At least once *(Mara moja)*<br>☐ At least twice *(Mara mbili)*<br>☐ More than twice *(Zaidi ya mara mbili)*<br>☐ Quarterly *(Mwisho wa robo wa kila mwaka)*<br>☐ N/A
**Q37 (a).** In the last supervisory visits, were disease surveillance activities in this facility reviewed?<br>(*Je, masuala ya shughuli za ufuatiliaji wa magonjwa yalikaguliwa katika ziara za mwisho za usimamizi wa kituo hiki cha afya?)*	☐ Yes *(Ndio)* ☐ No *(La)* ☐ DK *(Sijui)* ☐ N/A
**Q37 (b).** If NO, why do you think this was so?<br>(*Kama LA, sababu ni zipi?)*	
**Q37 (c).** If YES, were the reviewed disease surveillance activities appropriate for this facility level?<br>*(Kama NDIO, je ukaguzi hii ya shughuli za ufuatiliaji wa magonjwa yalikuwa ya kufaa kwa kiwango cha kituo hiki cha afya?)*	☐ Yes *(Ndio)* ☐ No *(La)* ☐ DK *(Sijui)* ☐ N/A
**Q37 (d).** If NO, why do you say so?<br>(*Kama LA, sababu ni zipi?)*	
**Q38 (a).** In the last supervisory visits, were PC-NTDs surveillance data reviewed or discussed?<br>*(Je, suala za ufuatiliaji wa magonjwa ya kitropiki yaliyo telekezwa zilikaguliwa katika ziara za mwisho za usimamizi wa kituo hiki cha afya?)*	☐ Yes *(Ndio)* ☐ No *(La)* ☐ DK *(Sijui)* ☐ N/A
**Q38 (b).** If NO, why was this so?<br>(*Kama LA, sababu ni zipi?)*	
**Q38 (c).** If YES, did you receive a supervisory report on PC-NTDs surveillance performance in this facility?	☐ Yes *(Ndio)* ☐ No *(La)* ☐ DK *(Sijui)* ☐ N/A

(Kama NDIO, je mulipokea ripoti maalum ya ufuatiliaji wa magonjwa haya ya kitropiki yaliyo telekezwa baada ya ziara za mwisho za usimamizi wa kituo hiki cha afya?)	
**Q38 (d).** If NO, why was this so?<br>(*Kama LA, sababu ni zipi?)*	
**Q39 (a).** In the last supervisory visits, were there any recommendations made concerning PC-NTDs surveillance in this facility?<br>*(Je, kulikuwepo na mapendekezo zozote kuhusu ufuatiliaji wa magonjwa ya kitropiki yaliyo telekezwa wakati wa ziara za mwisho za usimamizi wa kituo hiki cha afya?)*	☐ Yes *(Ndio)* ☐ No *(La)* ☐ DK *(Sijui)* ☐ N/A
**Q39 (b).** If YES, what were some of the recommendations made?<br>*(Kama NDIO, je mapendekezo hizo zilikuwa zipi?)*	
**Q39 (c).** In the last supervisory visits, were there any follow-ups on recommendations concerning PC-NTDs surveillance made from a previous visit in this facility?<br>*(Je, kulikuwepo na ufuatiliaji wowote wa mapendekezo yaliyotolewa kuhusu ufuatiliaji wa magonjwa ya kitropiki yaliyo telekezwa wakati wa ziara za mwisho za usimamizi wa kituo hiki cha afya?)*	☐ Yes *(Ndio)* ☐ No *(La)* ☐ DK *(Sijui)* ☐ N/A
**Q39 (d).** If YES, what did the follow-up entail?<br>*(Kama NDIO, je ufuatiliaji huu uligusia nini haswa?)*	
**Q40 (a).** Does this facility conduct supervisory visits regarding disease surveillance activities at the lower levels?<br>*(Je, kituo hiki cha afya kina tekeleza ziara za usimamizi kuhusu shughuli za ufuatiliaji wa magonjwa katika kiwango cha jamii?)*	☐ Yes *(Ndio)* ☐ No *(La)* ☐ DK *(Sijui)* ☐ N/A
**Q40 (b).** If NO, why is this so?<br>(*Kama LA, sababu ni zipi?)*	
**Q40 (c).** If YES, how often has this facility conducted supervisory visits regarding disease surveillance activities at the lower levels in the past one year?<br>*(Kama NDIO, je kwa mwaka uliopita kituo hiki cha afya kilitekeleza kwa mara ngapi ziara hizi za usimamizi wa shughuli za ufuatiliaji wa magonjwa katika kiwango cha jamii?)*	☐ At least once *(Mara moja)*<br>☐ At least twice *(Mara mbili)*<br>☐ More than twice *(Zaidi ya mara mbili)*<br>☐ Quarterly *(Mwisho wa robo wa kila mwaka)*<br>☐ N/A
**Q40 (d).** What is the recommended number of supervisory visits that need to be undertaken at the lower levels by this facility in a period of one year?<br>*(Je, inapendekezwa kituo hiki cha afya itimize shughuli za ziara za usimamizi katika kiwango cha jamii kwa mara ngapi kwa mwaka?)*	☐ At least once *(Mara moja)*<br>☐ At least twice *(Mara mbili)*<br>☐ More than twice *(Zaidi ya mara mbili)*<br>☐ Quarterly *(Mwisho wa robo wa kila mwaka)*<br>☐ Specify (Baini): …………………………………….<br>☐ DK *(Sijui)*<br>☐ N/A *(Si husika)*
**Q40 (e).** Is there a schedule/plan for future supervisory visits to the lower levels in this facility?	☐ Yes *(Ndio)* ☐ No *(La)* ☐ DK *(Sijui)* ☐ N/A

(Je, katika kituo hiki cha afya kuna ratiba maalum ya kutekeleza ziara za usimamizi katika kiwango cha jamii kwa siku zijazo?)	
**Q40 (f).** If NO, why is this so?<br>(*Kama LA, sababu ni zipi?)*	
**Q41.** Who in this facility undertakes the supervisory visits at the lower levels?<br>*(Je, ni nani haswa katika kituo hiki cha afya anawajibika na ziara za usimamizi katika kiwango cha jamii?)*	☐ Medical Officer In-Charge *(Daktari mkuu)*<br>☐ Clinical Officer In-Charge *(Afisa mkuu wa kliniki)*<br>☐ Nurse In-Charge *(Muuguzi mkuu)*<br>☐ Public Health Staff *(Mfanyakazi wa afya ya umma)*<br>☐ Laboratory Staff *(Mfanyakazi wa maabara)*<br>☐ Health Records Management Staff *(Mfanyakazi wa kumbukumbu za afya)*<br>☐ Other Cadre *(Uajibu zinginezo)*:………………………<br>☐ N/A
**Q42 (a).** In the last lower level supervisory visits, were PC-NTDs surveillance activities reviewed?<br>*(Katika ziara za mwisho za usimamizi, je shughuli za ufuatiliaji wa magonjwa ya kitropiki yaliyo telekezwa zilikaguliwa?)*	☐ Yes *(Ndio)* ☐ No *(La)* ☐ DK *(Sijui)* ☐ N/A
**Q42 (b).** If NO, why was this so?<br>(*Kama LA, sababu ni zipi?)*	
**Q42 (c).** Was a supervisory feedback report on PC-NTDs surveillance activities given to the lower levels?<br>*(Je mulipokeza kiwango cha jamii ripoti maalum kufuatia ziara za mwisho za usimamizi wa shughuli za ufuatiliaji wa magonjwa ya kitropiki yaliyo telekezwa katika kiwango hicho?)*	☐ Yes *(Ndio)* ☐ No *(La)* ☐ DK *(Sijui)* ☐ N/A
**Q42 (d).** If No, why was this so?<br>(*Kama LA, sababu ni zipi?)*	
**Q43 (a).** Do you experience any challenges conducting supervisory activities at the lower levels?<br>*(Je, munapata changamoto zozote munapo tekeleza ziara za usimamizi katika kiwango cha jamii?)*	☐ Yes *(Ndio)* ☐ No *(La)* ☐ DK *(Sijui)* ☐ N/A
**Q43 (b).** If YES, what are some of these challenges?<br>*(Kama NDIO, je changamoto hizi ni zipi?)*	
**Q44.** In your own opinion, how can supervision of PC-NTDs surveillance activities from the higher levels to this facility be improved?<br>*(Je, kwa maoni yako binafsi, viwango vya afya vya juu zaidi vinaweza kuboresha ki vipi ziara za usimamizi wa shughuli za ufuatiliaji wa magonjwa ya kitropiki yaliyo telekezwa katika kituo hiki cha afya?)*	
**Q45.** In your own opinion, how can supervision of PC-NTDs surveillance activities by this facility to the lower levels be improved?<br>*(Je, kwa maoni yako binafsi, je kituo hiki cha afya kinaweza kuboresha ki vipi ziara za usimamizi wa shughuli za ufuatiliaji wa magonjwa ya kitropiki yaliyo telekezwa katika kiwango cha jamii?*	
**TRAINING *(MAFUNZO)***	

**Q46 (a).** In your basic training were you trained on disease surveillance?<br>*(Je, katika elimu yako ya msingi ulipata mafunzo ya ufuatiliaji wa magonjwa?)*	☐ Yes *(Ndio)* ☐ No *(La)* ☐ DK *(Sijui)* ☐ N/A
**Q46 (b).** If NO, why do you think this was so?<br>(*Kama LA, je sababu zako ni zipi?)*	
**Q47 (a).** Do you feel your basic training is sufficient for you to adequately undertake disease surveillance activities in this facility?<br>*(Je, unahisi mafunzo yako ya msingi uliyopata yalikuwa ya kutosha kutimiza ufuatiliaji wa magonjwa katika kituo hiki cha afya?)*	☐ Yes *(Ndio)* ☐ No *(La)* ☐ DK *(Sijui)* ☐ N/A
**Q47 (b).** If NO, why do you say so?<br>(*Kama LA, sababu zako ni zipi?)*	
**Q48 (a).** Is your basic training applicable to PC-NTDs surveillance in this facility?<br>*(Je, unahisi mafunzo yako ya msingi uliyopata inatumika kwa kutimiza ufuatiliaji wa magonjwa ya kitropiki yaliyo telekezwa munazoshuhudia katika kituo hiki cha afya?)*	☐ Yes *(Ndio)* ☐ No *(La)* ☐ DK *(Sijui)* ☐ N/A
**Q48 (b).** If NO, why do you say so?<br>(*Kama LA, je sababu zako ni zipi?)*	
**Q49 (a).** While working in this facility have you received any post-basic training on disease surveillance?<br>*(Je, kama mhudumu wa afya katika kituo hiki cha afya umepata mafunzo zaidi maalum kuhusiana na ufuatiliaji wa magonjwa kando na elimu yako ya msingi?)*	☐ Yes *(Ndio)* ☐ No *(La)* ☐ DK *(Sijui)* ☐ N/A
**Q49 (b).** If YES *(Kama NDIO),*<br><br>When were you last trained *(Ulipata mafunzo hayo ya mwisho lini?)*<br><br>Where was the training *(Ulipata mafunzo hayo wapi?)*<br><br>Who facilitated the training *(Nani aliwezesha mafunzo hayo?)*<br><br>What was the duration of the training *(Mafunzo hayo yalikua kwa muda gani?)*	……………………………………………………..☐ N/A<br><br>……………………………………………………..☐ N/A<br><br>……………………………………………………..☐ N/A<br><br>……………………………………………………..☐ N/A
**Q49 (c).** Which specific disease surveillance and response elements were covered in your last post-basic training?<br>*(Je mafunzo hayo yaliangazia mambo yapi haswa kuhusu ufuatiliaji wa magonjwa?)*	☐ Case Detection *(Ugunduzi wa kesi za magonjwa)*<br>☐ Case Registration *(Usajili wa kesi za magonjwa)*<br>☐ Case Confirmation *(Uthibitisho wa kesi za magonjwa)*<br>☐ Reporting *(Kuripoti kesi za magonjwa)*<br>☐ Data Analysis *(Uchanganuzi wa kesi za magonjwa)*<br>☐ Outbreak Investigation *(Uchunguzi wa kuzuka kwa magonjwa)*<br>☐ Response and Control *(Uajibu na udhibiti wa mgonjwa)*<br>☐ Others *(Mada zinginezo)*:…………………………….<br>☐ DK *(Sijui)*

	☐ N/A *(Si Husika)*
**Q49 (d).** In your last post-basic training, was disease surveillance and response specific to PC-NTDs covered? *(Je, mafunzo hayo ya mwisho yaliangazia ufuatiliaji wa magonjwa ya kitropiki yaliyo telekezwa?)*	☐ Yes *(Ndio)* ☐ No *(La)* ☐ DK *(Sijui)* ☐ N/A
**Q49 (e).** If YES, which specific elements regarding PC-NTDs surveillance and response were covered in the training? *(Kama NDIO, je ni mambo yapi haswa yaliangaziwa kuhusiana na ufuatiliaji wa magonjwa ya kitropiki yaliyo telekezwa?)*	☐ Case Detection *(Ugunduzi wa kesi za magonjwa)*<br>☐ Case Registration *(Usajili wa kesi za magonjwa)*<br>☐ Case Confirmation *(Uthibitisho wa kesi za magonjwa)*<br>☐ Reporting *(Kuripoti kesi za magonjwa)*<br>☐ Data Analysis *(Uchanganuzi wa kesi za magonjwa)*<br>☐ Outbreak Investigation *(Uchunguzi wa kuzuka kwa magonjwa)*<br>☐ Response and Control *(Uajibu na udhibiti wa mgonjwa)*<br>☐ Others *(Mada zinginezo)*:…………………………….<br>☐ N/A
**Q49 (f).** If NO, would you be interested in a training on PC-NTDs surveillance and response? *(Kama LA, je unania ya mafunzo inayoangazia ufuatiliaji wa magonjwa ya kitropiki yaliyo telekezwa?)*	☐ Yes *(Ndio)* ☐ No *(La)*
**Q49 (g).** If YES, which specific aspect/s regarding PC-NTDs surveillance and response would you like the training to focus on amongst those mentioned in this interview? *(Kama NDIO, je ni mambo yapi haswa ungependa mafunzo hayo ya angazie kuhusiana na ufuatiliaji wa magonjwa ya kitropiki yaliyo telekezwa?)*	☐ Case Detection *(Ugunduzi wa kesi za magonjwa)*<br>☐ Case Registration *(Usajili wa kesi za magonjwa)*<br>☐ Case Confirmation *(Uthibitisho wa kesi za magonjwa)*<br>☐ Reporting *(Kuripoti kesi za magonjwa)*<br>☐ Data Analysis *(Uchanganuzi wa kesi za magonjwa)*<br>☐ Outbreak Investigation *(Uchunguzi wa kuzuka kwa magonjwa)*<br>☐ Response and Control *(Uajibu na udhibiti wa mgonjwa)*<br>☐ Others *(Mada zinginezo)*:…………………………….<br>☐ DK (Sijui)<br>☐ N/A
**Q50 (a).** Are there any challenges facing organizing post-basic training of health personnel in this facility? *(Je, kuna chamgamoto zozote zinazokumba maandalizi ya mafunzo ya wafanyakazi wa afya katika kituo hiki cha afya?)*	☐ Yes *(Ndio)* ☐ No *(La)* ☐ DK *(Sijui)* ☐ N/A
**Q50 (b).** If YES, what are some of these challenges? *(Kama NDIO, je changamoto hizi ni zipi?)*	
**Q51.** In your own opinion, how can post-basic training on PC-NTDs surveillance and response be improved in this facility?	

(Kwa maoni yako binafsi, je mafunzo yanayoangazia ufuatiliaji wa magonjwa ya kitropiki yaliyo telekezwa zinaeza kuboreshwa ki vipi katika kituo hiki cha afya?)	
**OPPORTUNITIES FOR IMPROVEMENT** ***(NAFASI ZA UBORESHAJI WA UFUATILIAJI WA MAGONJWA)***	
**Q52 (a).** Are you satisfied with PC-NTDs surveillance and response within the existing IDSR system in this region? *(Je, umeridhishwa na shughuli za ufuatiliaji wa magonjwa ya kitropiki yaliyo telekezwa katika mkoa huu vile zilivyo?)*	☐ Yes *(Ndio)* ☐ No *(La)* ☐ DK *(Sijui)* ☐ N/A
**Q52 (b).** If NO, why do you say so? *(Kama LA, tafadhali nipe sababu zako)?*	
**Q52 (c).** If NO, how can surveillance and response to PC-NTDs be improved within the existing IDSR system? *(Kama LA, je shughuli hizi za ufuatiliaji wa magonjwa ya kitropiki zinaweza kuboreshwa kwa njia zipi?)*	
**Q53.** In your own opinion, what will be the benefits of improving surveillance and response to PC-NTDs in this region? *(Kwa maoni yako binafsi, je faida za kuboresha shughuli hizi za ufuatiliaji wa magonjwa ya kitropiki yaliyo telekezwa katika mkoa huu ni zipi?)*	

HEALTH FACILITY OBSERVATION CHECKLIST

**O1.** Observed the availability of guideline/manual for disease surveillance	Yes ☐ No ☐ N/A ☐
**O2.** Observed that the disease surveillance guidelines/manual is of a recent version	Yes ☐ No ☐ N/A ☐
**O3.** Observed the availability of standard case definition for PC-NTDs	Yes ☐ No ☐ N/A ☐
**O4.** Observed case registers specific for registration of PC-NTD cases?	Yes ☐ No ☐ N/A ☐
**O5.** Observed correct registration of an PC-NTD case based on the standard case definition	Yes ☐ No ☐ N/A ☐
**O6.** Observed an existing rumour log (database for registration of suspected PC-NTDs from informal sources)	Yes ☐ No ☐ N/A ☐
**O7.** Observed the presence of equipment/materials required to collect specimen for PC-NTDs	Yes ☐ No ☐ N/A ☐
**O8.** Observed presence of transport media (e.g. cool box) for PC-NTDs specimen at the health facility	Yes ☐ No ☐ N/A ☐
**O9.** Observed presence of packing materials for shipment of PC-NTDs specimens to a higher level	Yes ☐ No ☐ N/A ☐
**O10.** Observed reports of PC-NTDs specimens sent to a higher level	Yes ☐ No ☐ N/A ☐
**O11.** Observed the presence of a laboratory facility	Yes ☐ No ☐ N/A ☐
**O12.** Observed that the laboratory is adequately equipped to confirm cases of PC-NTDs	Yes ☐ No ☐ N/A ☐
**O13.** Observed guidelines for specimen collection, packaging and transportation	Yes ☐ No ☐ N/A ☐
**O14.** Observed that the last monthly report agreed with the IDSR standard format for reporting PC-NTDs targeted for elimination and those of major public health importance	Yes ☐ No ☐ N/A ☐
**O15.** Observed that PC-NTDs reports were being sent before the deadline for submission	Yes ☐ No ☐ N/A ☐
**O16.** Observed “zero-reporting” specific to PC-NTDs in reporting forms sent to the next level	Yes ☐ No ☐ N/A ☐
**O17 (a).** Observed analysis of PC-NTDs surveillance data by age	Yes ☐ No ☐ N/A ☐
**O17 (b).** Observed analysis of PC-NTDs surveillance by sex	Yes ☐ No ☐ N/A ☐
**O17 (c).** Observed analysis of PC-NTDs surveillance by place (village/locality/sub-county)	Yes ☐ No ☐ N/A ☐
**O17 (d).** Observed analysis of PC-NTDs surveillance by time	Yes ☐ No ☐ N/A ☐
**O18.** Observed performance of trend analysis for PC-NTD cases	Yes ☐ No ☐ N/A ☐
**O19.** Observed plotting of line graph of PC-NTDs cases by time (predefined action thresholds)	Yes ☐ No ☐ N/A ☐
**O20.** Observed availability of demographic data of the PC-NTDs endemic region at the facility (E.g. population <5 yr., population by village, total popn)	Yes ☐ No ☐ N/A ☐
**O21.** Observed rates for PC-NTD cases based on demographic data of the endemic region	Yes ☐ No ☐ N/A ☐

**O22.** Observed the existence of a written case management guideline for at least one of the PC-NTD/s	Yes ☐ No ☐ N/A ☐
**O23.** Observed a case management report for an PC-NTD case	Yes ☐ No ☐ N/A ☐
**O24.** Observed reports of PC-NTD cases that met the action threshold	Yes ☐ No ☐ N/A ☐
**O25.** Observed reports of complete PC-NTDs case investigations	Yes ☐ No ☐ N/A ☐
**O26.** Observed feedback reports on PC-NTD/s or at least one report or bulletin from a higher level in the past one year	Yes ☐ No ☐ N/A ☐
**O27.** Observed copy of feedback reports on PC-NTD/s to the lower levels in the past one year	Yes ☐ No ☐ N/A ☐
**O28.** Observed copy of feedback reports on PC-NTD/s to the lower levels are based on a standard format	Yes ☐ No ☐ N/A ☐
**O29.** Observed the minutes or report on PC-NTDs of at least one meeting between the health facility team and community level representatives in the past one year	Yes ☐ No ☐ N/A ☐
**O30.** Observed supervision report/s or evidence of supervision on PC-NTDs surveillance activities in the past one year from the higher level	Yes ☐ No ☐ N/A ☐
**O31.** Observed copy of supervisory visits report/s on PC-NTDs surveillance activities conducted at the lower levels in the past one year	Yes ☐ No ☐ N/A ☐
**O32.** Observed a schedule for future supervisory visits to the lower levels	Yes ☐ No ☐ N/A ☐
**O33.** Observed availability of training manuals/modules for PC-NTDs surveillance and response	Yes ☐ No ☐ N/A ☐
**O34.** Observed availability of PC-NTDs surveillance and response training plans/schedules for healthcare workers	Yes ☐ No ☐ N/A ☐

**Resources**	**Available**			**Functional**			**Facilitates surveillance activities**		
**(a). Logistics & Power**	**Y**	**N**	**NA**	**Y**	**N**	**NA**	**Y**	**N**	**NA**
1.Electricity									
2.Motor vehicles									
3.Motor cycles									
4.Bicycles									
**(b). Data Management**									
1.Stationery									
2.Calculators									
3.Computers									
4.Printers									
5.Photocopier									
6.Data Analysis Software (e.g. Ms Excel)									
**(c). Communication**									
1.Telephone/Mobile Services									
2.Fax services									
3.Internet services									
**(d). Information Education & Communication**									
1.Posters									
2.Pamphlets									
3.Flipcharts									
4.Electronic Visual Aids									

SUB-COUNTY LEVEL QUESTIONNAIRE (*DODOSO LA KIWANGO CHA KAUNTI NDOGO)*

**GENERAL INFORMATION**<br>***(Habari kwa ujumla)***	**Please mark (X) in the appropriate box**<br>***(Tafadhali weka alama ya tiki (√) katika sehemu inayofaa zaidi)***	
Region<br>*(Eneo)*	……………………………………… Sub-County<br>*(Mkoa mdogo)*	
Demographic Characteristics<br>*(Demografia ya mhojiwa)*	Age *(Umri)*	☐ 18-30 ☐ 31-40<br>☐ 41-50 ☐ >50
	Sex *(Jinsia)*	Male *(Kiume)* ☐ Female *(Kike)* ☐
**Q1.** What is your current designation?<br>*(Wajibu wa mfanyakazi wa afya)*	…………………………………………………	
Number of years worked in your current designation<br>*(Miaka yako kikazi katika uajibu huu?)*	☐ Less than a year *(Chini ya mwaka 1)*<br>☐ 1-2 years *(Kati ya mwaka 1-2)*<br>☐ 2-3 years *(Kati ya miaka 2-3)*<br>☐ 3-5 years *(Kati ya miaka 3-5)*<br>☐ More than 5years *(Zaidi ya miaka 5)*	
**Q2.** What is your highest level of education?<br>*(Kiwango chako cha juu zaidi katika masomo?)*	☐ PhD *(Shahada ya uzamifu)*<br>☐ Masters *(Shahada ya uzamili)*<br>☐ Degree *(Shahada)*<br>☐ Diploma *(Stashahada)*<br>☐ Certificate *(Astashahada)*	
**DISEASE SURVEILLANCE (*UFUATILIAJI WA MAGONJWA)***		
**Q3.** What do you understand by the term disease surveillance?<br>*(Je unaelewa vipi ufuatiliaji wa magonjwa?)*		
**Q4.** What do you understand by the term neglected tropical diseases?<br>*(Je, unaelewa ki vipi istilahi ya magonjwa ya kitropiki yaliyo telekezwa?)*		
**Q5 (a).** Are you aware of any neglected tropical diseases in this sub-county?<br>*(Je unafahamu magonjwa haya ya kitropiki yaliyo telekezwa katika kaunti hii ndogo?)*	☐ Yes *(Ndio)* ☐ No *(La)* ☐ DK *(Sijui)*	
**Q5 (b).** If YES, which are the preventive chemotherapy targeted neglected tropical diseases (PC-NTDs) common in this sub-county?	☐ Lymphatic Filariasis *(Matende)*<br>☐ Soil Transmitted Helminths *(Minyoo)*<br>☐ Trachoma *(Trakoma)*	

(Kama NDIO, je magonjwa haya ya kitropiki yaliyo telekezwa ambazo ni za kawaida katika kaunti hii ndogo ni zipi?)	☐ Schistosomiasis *(Kichocho)*<br>☐ Others *(Magonjwa zinginezo)*: ………………………....<br>☐ Don't Know *(Sijui)*<br>☐ N/A *(Si Husika)*
**Q5 (c).** Which of the PC-NTDs mentioned above are commonly reported in this sub-county?<br>*(Je ni magonjwa yapi ya kitropiki yaliyo telekezwa ulizo taja zinaripotiwa katika kaunti hii ndogo?)*	☐ Lymphatic Filariasis *(Matende)*<br>☐ Soil Transmitted Helminths *(Minyoo)*<br>☐ Trachoma *(Trakoma)*<br>☐ Schistosomiasis *(Kichocho)*<br>☐ Others *(Magonjwa zinginezo)*: ………………………....<br>☐ Don't Know *(Sijui)*<br>☐ N/A *(Si Husika)*
**Q6 (a).** Is there a functional disease surveillance system present in this sub-county?<br>*(Je kuna mfumo maalum ya ufuatiliaji wa magonjwa katika kaunti hii ndogo?)*	Yes *(Ndio)* ☐ No *(La)* ☐ DK *(Sijui)* ☐ N/A *(Si Husika)* ☐
**Q6 (b).** If NO, why do you say so?<br>*(Kama LA, tafadhali nipe sababu zako?)*	
**Q7 (a).** Are you aware of the integrated disease surveillance and response (IDSR) system?<br>*(Je unaelewa vipi mfumo wa IDSR?)*	Yes *(Ndio)* ☐ No *(La)* ☐ DK *(Sijui)* ☐ N/A *(Si Husika)* ☐
**Q7 (b).** If YES, are any of the above mentioned PC-NTDs reported through the IDSR system?<br>*(Kama NDIO, je magonjwa ya kitropiki yaliyo telekezwa zinazoripotiwa kupitia mfumo huu?)*	Yes *(Ndio)* ☐ No *(La)* ☐ DK *(Sijui)* ☐ N/A *(Si Husika)* ☐
**Q7 (c).** If NO, why is this so?<br>*(Kama LA, sababu ni zipi?)*	
**Q7 (d).** If YES, specifically which PC-NTDs are reported through this system?<br>*(Kama NDIO, je ni magonjwa yapi ya kitropiki yaliyo telekezwa zinazoripotiwa kupitia mfumo huu?)*	☐ Lymphatic Filariasis *(Matende)*<br>☐ Soil Transmitted Helminths *(Minyoo)*<br>☐ Trachoma *(Trakoma)*<br>☐ Schistosomiasis *(Kichocho)*<br>☐ Others *(Magonjwa zinginezo)*: ………………………....<br>☐ Don't Know *(Sijui)*<br>☐ N/A *(Si Husika)*
**Q8 (a).** In your own opinion, are PC-NTDs surveillance data adequately reported within the IDSR system in this sub-county?<br>*(Kwa maoni yako binafsi, je magonjwa haya ya kitropiki yaliyo telekezwa zinaripotiwa kwa njia mwafaka kupitia mfumo wa IDSR katika kaunti hii ndogo?)*	Yes *(Ndio)* ☐ No *(La)* ☐ DK *(Sijui)* ☐ N/A *(Si Husika)* ☐
**Q8 (b).** If NO, why do you say so?<br>*(Kama LA, sababu ni zipi?)*	

**SURVEILLANCE MANUALS & CASE DEFINITIONS *(MIONGOZO ZA UFUATILIAJI NA KESI ZA MAGONJWA)***	
**Q9 (a).** Is there a manual for disease surveillance present in this sub-county? *(Je, kuna miongozo maalum za ufuatiliaji wa magonjwa katika kaunti hii ndogo?)*	Yes *(Ndio)* ☐ No *(La)* ☐ DK *(Sijui)* ☐ N/A *(Si Husika)* ☐
**Q9 (b).** If NO, why is this so? *(Kama LA, sababu ni zipi?)*	
**Q9 (c).** If YES, is the disease surveillance manual in use of a recent version? *(Kama NDIO, je miongozo hizi za ufuatiliaji wa magonjwa ni za hivi karibuni?)*	Yes *(Ndio)* ☐ No *(La)* ☐ DK *(Sijui)* ☐ N/A *(Si Husika)* ☐
**Q9 (d).** Is the manual useful in guiding disease surveillance activities at this sub-county level? *(Je, miongozo hizi zinaelekeza vyema ufuatiliaji wa magonjwa katika kaunti hii ndogo?)*	Yes *(Ndio)* ☐ No *(La)* ☐ DK *(Sijui)* ☐ N/A *(Si Husika)* ☐
**Q9 (e).** If NO, why is this so? *(Kama LA, sababu ni zipi?)*	
**Q9 (f).** Is the disease surveillance manual easy to use? *(Je, miongozo hizi za ufuatiliaji wa magonjwa ni rahisi kutumia?)*	Yes *(Ndio)* ☐ No *(La)* ☐ DK *(Sijui)* ☐ N/A *(Si Husika)* ☐
**Q9 (g).** If NO, why do you say so? *(Kama LA, sababu ni zipi?)*	
**Q9 (h).** Does the manual specifically guide PC-NTDs surveillance activities in this sub-county? *(Je, miongozo hizi zinaelekeza ufuatiliaji wa magonjwa ya kitropiki yaliyo telekezwa katika kaunti hii ndogo?)*	Yes *(Ndio)* ☐ No *(La)* ☐ DK *(Sijui)* ☐ N/A *(Si Husika)* ☐
**Q9 (i).** If NO, why do you think so? *(Kama LA, sababu ni zipi?)*	
**Q10 (a).** Are PC-NTDs case definitions available in this sub-county? *(Je, miongozo za kutambua kesi za magonjwa ya kitropiki yaliyo telekezwa zinapatikana kwa urahisi katika kaunti hii ndogo?)*	Yes *(Ndio)* ☐ No *(La)* ☐ DK *(Sijui)* ☐ N/A *(Si Husika)* ☐
**Q10 (b).** If NO, why is this so? *(Kama LA, sababu ni zipi?)*	
**Q10 (c).** If YES, describe the availability of case definitions for the following PC-NTDs: *(Kama NDIO, tafadhali eleza kama miongozo za kutambua kesi zifuatazo za magonjwa ya kitropiki yaliyo telekezwa zinapatikana kwa urahisi*?)	
1. Lymphatic Filariasis *(Matende)*	Yes *(Ndio)* ☐ No *(La)* ☐ DK *(Sijui)* ☐ N/A *(Si Husika)* ☐
2. Soil Transmitted Helminths *(Minyoo)*	Yes *(Ndio)* ☐ No *(La)* ☐ DK *(Sijui)* ☐ N/A *(Si Husika)* ☐
3. Trachoma *(Trakoma)*	Yes *(Ndio)* ☐ No *(La)* ☐ DK *(Sijui)* ☐ N/A *(Si Husika)* ☐

4. Schistosomiasis *(Kichocho)*	Yes *(Ndio)* ☐ No *(La)* ☐ DK *(Sijui)* ☐ N/A *(Si Husika)* ☐
5. Other PC-NTDs: ………………………………….. *(Magonjwa zinginezo)*	Yes *(Ndio)* ☐ No *(La)* ☐ DK *(Sijui)* ☐ N/A *(Si Husika)* ☐
**CASE CONFIRMATION *(UTHIBITISHO WA KESI ZA MAGONJWA)***	
**Q11 (a).** Are there an adequate number of functional laboratories in this sub-county? *(Je kuna maabara za kutosha zinaozofanya kazi katika kaunti hii ndogo?)*	Yes *(Ndio)* ☐ No *(La)* ☐ DK *(Sijui)* ☐ N/A *(Si Husika)* ☐
**Q11 (b).** If NO, why do you say so? *(Kama LA, sababu ni zipi?)*	
**Q11 (c).** If YES, are the laboratories adequately equipped to confirm cases of PC-NTDs? *(Kama NDIO, je maabara haya yanavifaa vya kutosha vya kudhibitisha magonjwa ya kitropiki yaliyo telekezwa?)*	Yes *(Ndio)* ☐ No *(La)* ☐ DK *(Sijui)* ☐ N/A *(Si Husika)* ☐
**Q11 (d).** If NO, why do you say so? *(Kama LA, sababu ni zipi?)*	
**Q12 (a).** Does this sub-county have guidelines for specimen collection, handling, storage and transportation to the next higher level? (*Je kaunti hii ndogo ina miongozo za ukusanyaji, utunzaji, uhifaji na usafirishaji wa sampuli za magonjwa kuelekezwa katika maabara za juu zaidi?)*	Yes *(Ndio)* ☐ No *(La)* ☐ DK *(Sijui)* ☐ N/A *(Si Husika)* ☐
**Q12 (b).** If NO, why is this so? *(Kama LA, sababu ni zipi?)*	
**Q13 (a).** Are there any challenges facing PC-NTDs specimen collection, storage and transportation in this sub-county? *(Je, kuna changamoto zozote zinazokumba ukusanyaji, utunzaji, uhifaji na usafirishaji wa sampuli za magonjwa za kitropiki yaliyo telekezwa katika kaunti hii ndogo?)*	Yes *(Ndio)* ☐ No *(La)* ☐ DK *(Sijui)* ☐ N/A *(Si Husika)* ☐
**Q13 (b).** If YES, what are some of these challenges? *(Kama NDIO, changamoto hizi ni zipi?)*	
**Q14 (a).** Does this sub-county have the capacity to transport PC-NTDs specimens to a higher level laboratory? *(Je kaunti hii ndogo ina uwezo wa kusafirisha sampuli za magonjwa ya kitropiki yaliyo telekezwa kwa maabara za juu zaidi?)*	Yes *(Ndio)* ☐ No *(La)* ☐ DK *(Sijui)* ☐ N/A *(Si Husika)* ☐
**Q14 (b).** Do you make follow-ups on PC-NTDs specimens at this level? *(Je, munafuatilia matokeo ya sampuli hizi za magonjwa haya ya kitropiki yaliyo telekezwa katika kaunti hii ndogo?)*	Yes *(Ndio)* ☐ No *(La)* ☐ DK *(Sijui)* ☐ N/A *(Si Husika)* ☐
**Q14 (c).** If NO, why is this so? *(Kama LA, sababu ni zipi?)*	

**Q14 (d).** Do you receive reports for PC-NTDs specimens sent to the higher level laboratory? *(Je, munapokea matokeo ya sampuli hizi za magonjwa ya kitropiki yaliyo telekezwa kutoka kwa maabara za juu zaidi?)*	Yes *(Ndio)* ☐ No *(La)* ☐ DK *(Sijui)* ☐ N/A *(Si Husika)* ☐
**Q14 (e).** If NO, why is this so? *(Kama LA, sababu ni zipi?)*	
**Q14 (f).** Are the reports received from the higher level laboratory complete and elaborate? *(Je, matokeo ya sampuli hizi kutoka kwa maabara za juu zaidi ni kamili na zenye ufafanuzi tosha?)*	Yes *(Ndio)* ☐ No *(La)* ☐ DK *(Sijui)* ☐ N/A *(Si Husika)* ☐
**Q14 (g).** If NO, why do you say so? *(Kama LA, sababu ni zipi?)*	
**Q14 (h).** Are the reports received from the higher-level laboratory timely? *(Je, matokeo ya sampuli hizi kutoka kwa maabara za juu zaidi munazipata kwa wakati unaofaa?*	Yes *(Ndio)* ☐ No *(La)* ☐ DK *(Sijui)* ☐ N/A *(Si Husika)* ☐
**Q14 (i).** If NO, why do you say so? *(Kama LA, sababu ni zipi?)*	
**SURVEILLANCE DATA REPORTING *(KURIPOTI KWA HABARI ZA UFUATILIAJI WA MAGONJWA)***	
**Q15.** Which form of reporting is used to report surveillance data to the next level in this sub-county? *(Je, ni mfumo upi unaotumika kuripoti habari za ufuatiliaji wa magonjwa kuelekea katika kiwango cha juu zaidi?)*	☐ Paper-based *(Kwa njia za karatasi)*<br>☐ Electronic-based (*Kwa njia za kielektroniki)*<br>☐ Both Paper and Electronic-based (*Kwa njia za karatasi na za kielektroniki)*<br>☐ DK *(Sijui)*<br>☐ N/A *(Si Husika)*
**Q16 (a).** Has this sub-county lacked disease surveillance reporting forms at any given time in the past six months? *(Je, mushawahi kumbwa na ukosefu wa fomu hizi za kuripoti magonjwa kwa wakati wowote kwa muda wa miezi sita zilizopita?)*	Yes *(Ndio)* ☐ No *(La)* ☐ DK *(Sijui)* ☐ N/A *(Si Husika)* ☐
**Q16 (b).** If YES, why was this so? (*Kama NDIO, sababu ni zipi?)*	
**Q17 (a).** Are the surveillance reporting forms adequate for reporting PC-NTDs? *(Je fomu hizi zinautoaji tosha wa kuripoti magonjwa haya ya kitropiki yaliyo telekezwa)?*	Yes *(Ndio)* ☐ No *(La)* ☐ DK *(Sijui)* ☐ N/A *(Si Husika)* ☐
**Q17 (b).** If NO, why do you say so? *(Kama LA, sababu ni zipi?)*	
**Q18 (a).** Are the surveillance reporting forms easy to complete? *(Je, ni rahisi kukamilisha fomu hizi za kuripotia magonjwa?)*	Yes *(Ndio)* ☐ No *(La)* ☐ DK *(Sijui)* ☐ N/A *(Si Husika)* ☐
**Q18 (b).** If NO, why do you say so?	

(Kama LA, sababu ni zipi?)	
**Q19.** How often are PC-NTDs surveillance reports prepared and sent to the county level from this sub-county?<br>*(Je, ripoti za ufuatiliaji wa magonjwa ya kitropiki yaliyo telekezwa kueleka kiwango cha kaunti zinatumwa kwa kipindi ipi mara kwa mara?)*	☐ Weekly *(Kwa kila wiki)*<br>☐ Monthly *(Kwa kila mwezi)*<br>☐ Quarterly *(Kwa kila robo wa mwisho wa mwezi)*<br>☐ Bi-annually *(Mara mbili kwa mwaka)*<br>☐ Annually *(Mara moja kwa mwaka)*<br>☐ DK *(Sijui)*<br>☐ N/A *(Si Husika)*
**Q20 (a).** Are there deadlines for sending PC-NTDs surveillance reports to the county level?<br>*(Je kuna tarehe za mwisho za kutuma ripoti za ufuatiliaji wa magonjwa ya kitropiki yaliyo telekezwa kueleka kiwango cha kaunti?)*	Yes *(Ndio)* ☐ No *(La)* ☐ DK *(Sijui)* ☐ N/A *(Si Husika)* ☐
**Q20 (b).** If YES, what are the deadlines for submitting PC-NTDs surveillance reports to the county level?<br>*(Kama NDIO, je tarehe za mwisho za kutuma ripoti hizi ni zipi?)*	☐ Beginning of every week *(Mwanzo wa kila wiki)*<br>☐ End of every week *(Mwisho wa kila wiki)*<br>☐ Beginning of every month *(Mwanzo wa kila mwezi)*<br>☐ End of every month *(Mwisho wa kila mwezi)*<br>☐ End of every quarter *(Mwisho wa kila robo wa mwaka)*<br>☐ End of every year *(Mwisho wa kila mwaka)*<br>☐ DK *(Sijui)*<br>☐ N/A *(Si Husika)*
**Q20 (c).** Do you often meet the deadlines for submission of PC-NTDs surveillance reports to the county level?<br>*(Je munafaulu kwa mara nyingi kutuma ripoti hizi za ufuatiliaji wa magonjwa ya kitropiki yaliyo telekezwa kueleka kiwango cha kaunti kwa wakati mwafaka?)*	Yes *(Ndio)* ☐ No *(La)* ☐ DK *(Sijui)* ☐ N/A *(Si Husika)* ☐
**Q20 (d).** If NO, what are the reasons for not meeting the deadlines?<br>*(Kama LA, sababu ni zipi?)*	
**Q21 (a).** Are the PC-NTDs surveillance reports from the health facilities accurate?<br>*(Je, ripoti za ufuatiliaji wa magonjwa ya kitropiki yaliyo telekezwa kutoka kwa viwango vya vituo vya afya huwa sahihi?)*	Yes *(Ndio)* ☐ No *(La)* ☐ DK *(Sijui)* ☐ N/A *(Si Husika)* ☐
**Q21 (b).** If NO, why do you say so?<br>*(Kama LA, sababu ni zipi?)*	
**Q22 (a).** Who prepares PC-NTDs surveillance reports in this sub-county?<br>*(Nani anahusika na utayarishaji wa ripoti hizi za ufuatiliaji wa magonjwa ya kitropiki yaliyo telekezwa katika kaunti hii ndogo?)*	☐ Sub County Disease Surveillance Coordinator *(Msimamizi wa shughuli za ufuatiliaji wa magonjwa katika kaunti ndogo)*<br>☐ Sub County Public Health Nurse

	(Msimamizi wa wauguzi wa afya ya jamii katika kaunti ndogo)<br>☐ Sub County Health Records and Information Officer *(Msimamizi wa habari za afya katika kaunti ndogo)*<br>☐ Sub County Public Health Officer *(Msimamizi wa afya ya umma katika kaunti ndogo)*<br>☐ Other cadre *(Wenye wajibu zinginezo):* ………………………<br>☐ DK *(Sijui)*<br>☐ N/A *(Si Husika)*
**Q22 (b).** Is he/she trained in preparing PC-NTDs surveillance reports?<br>*(Je, anaye husika na utayarishaji wa ripoti hizi za ufuatiliaji wa magonjwa ikiwemo ya kitropiki yaliyo telekezwa amepata mafunzo maalum?)*	Yes *(Ndio)* ☐ No *(La)* ☐ DK *(Sijui)* ☐ N/A *(Si Husika)* ☐
**Q23 (a).** Is there a standard surveillance reporting format for PC-NTDs used in this sub-county?<br>*(Je, kuna muundo maluum unaotumika katika kaunti hii ndogo wa kuripoti magonjwa haya ya kitropiki yaliyo telekezwa?)*	Yes *(Ndio)* ☐ No *(La)* ☐ DK *(Sijui)* ☐ N/A *(Si Husika)* ☐
**Q23 (b).** If NO, why is this so?<br>*(Kama LA, sababu ni zipi?)*	
**Q24 (a).** Is "zero-reporting" done for instances where there were no reportable PC-NTD cases at a given time in this sub-county?<br>(*Je ripoti za sufuri zinawasilishwa kwa mfano pasipo magonjwa haya ya kitropiki yaliyo telekezwa kwa wakati wowote katika kaunti hii ndogo?)*	Yes *(Ndio)* ☐ No *(La)* ☐ DK *(Sijui)* ☐ N/A *(Si Husika)* ☐
**Q24 (b).** If NO, why is this not done?<br>*(Kama LA, sababu ni zipi?)*	
**Q25 (a).** Through which channel/s do you report PC-NTDs surveillance data from this sub-county to the county level?<br>*(Je, unatumia njia zipi kuripoti habari za ufuatiliaji wa magonjwa haya ya kitropiki yaliyo telekezwa kuzielekeza katika kiwango cha juu?)*	☐ In Person *(Katika mtu/Mwenyewe)*<br>☐ Phone Call *(Kupiga simu)*<br>☐ Mobile SMS *(Kutuma ujumbe fupi kwa kutumia simu ya rununu)*<br>☐ Email *(Kutumia barua pepe)*<br>☐ eIDSR/DHIS *(Njia za kielektroniki)*<br>☐ Other *(Njia zinginezo)*: ……………………………..<br>☐ N/A
**Q25 (b).** Are there challenges experienced reporting PC-NTDs surveillance data through this channel/s?<br>*(Je, kuna changamoto zozote zinazo kumba njia hizi za kuripoti habari za ufuatiliaji wa magonjwa ya kitropiki yaliyo telekezwa ulizotaja?)*	Yes *(Ndio)* ☐ No *(La)* ☐ DK *(Sijui)* ☐ N/A *(Si Husika)* ☐
**Q25 (c).** If YES, what are some of these challenges?<br>*(Kama NDIO, tafadhali taja changamoto hizi)*	

**Q26.** In your own opinion, how can reporting of PC-NTDs surveillance data be improved in this sub-county?<br>*(Kwa maoni yako binafsi, kuripoti kwa habari za ufuatiliaji wa magonjwa ya kitropiki yaliyo telekezwa zinaweza kuimarishwa kwa njia zipi katika kaunti hii ndogo?)*	
**DATA ANALYSIS *(UCHANGANUZI WA HABARI ZA UFUATILIAJI WA MAGONJWA)***	
**Q27 (a).** Do you analyse surveillance data for PC-NTDs at this sub-county level?<br>*(Je, kwa kawaida huwa munachanganua habari za ufuatiliaji wa magonjwa ya kitropiki yaliyo telekezwa katika kaunti hii ndogo?*	Yes *(Ndio)* ☐ No *(La)* ☐ DK *(Sijui)* ☐ N/A *(Si Husika)* ☐
**Q27 (b).** If YES, how is the data analysed?<br>*(Kama NDIO, munachanganua habari hizi mulizo kusanya kwa njia zipi?)*	☐ By age *(Kwa umri)*<br>☐ By sex *(Kwa jinsia)*<br>☐ By place *(Kwa makao)*<br>☐ By time *(Kwa saa)*<br>☐ Others *(Kwa njia zinginezo):……………………………*<br>☐ N/A
**Q28 (a).** Does this sub-county perform trend analysis for PC-NTD cases reported from the health facilities?<br>*(Je, munachanganua mienendo ya magonjwa haya ya kitropiki yaliyo telekezwa katika kaunti hii ndogo?)*	Yes *(Ndio)* ☐ No *(La)* ☐ DK *(Sijui)* ☐ N/A *(Si Husika)* ☐
**Q28 (b).** If NO, why is this not done?<br>*(Kama LA, sababu ni zipi?)*	
**Q29 (a).** Do you have an action threshold for the PC-NTDs reported in this sub-county?<br>*(Je, munavizingiti vya matendo katika kaunti hii ndogo inayofuata kuripotiwa kwa magonjwa haya ya kitropiki yaliyo telekezwa?)*	Yes *(Ndio)* ☐ No *(La)* ☐ DK *(Sijui)* ☐ N/A *(Si Husika)* ☐
**Q29 (b).** If YES, what was the action threshold for PC-NTDs reported in the past year?<br>*(Kama NDIO, je vizingiti hivi vya matendo ambavyo vilifuata kuripotiwa kwa magonjwa ya kitropiki yaliyo telekezwa kwa mwaka uliyopita ni yapi?)*	☐ Number of cases *(Nambari ya kesi za magonjwa)*<br>☐ Percentage increase in number of cases *(Ongezeko wa asilimia fulani ya kesi za magonjwa)*<br>☐ Rates *(Kulingana na viwango maalum)*<br>☐ N/A
**Q29 (c).** What action followed the last PC-NTD/s reported from the health facilities, which met the action threshold in this sub-county in the past year?<br>*(Je, kwa mwaka uliyopita ni matendo yapi yalifuatilia kuripotiwa kwa magonjwa ya kitropiki yaliyo telekezwa ambazo zilitimia vizingiti vya matendo yaliyowekwa?)*	………………………………………………………………………..<br>☐ DK *(Sijui)* ☐ N/A *(Si husika)*
**Q30 (a).** Are you satisfied with the analysis done for PC-NTDs surveillance data at this sub-county level?<br>*(Je, umeridhishwa na uchanganuzi wa habari za ufuatiliaji wa magonjwa haya ya kitropiki yaliyo telekezwa katika kaunti hii ndogo?)*	Yes *(Ndio)* ☐ No *(La)* ☐ DK *(Sijui)* ☐ N/A *(Si Husika)* ☐

**Q30 (b).** If NO, why do you so?<br>*(Kama LA, sababu ni zipi?)*	
**Q31 (a).** Are there challenges facing analysis of PC-NTDs surveillance data at this sub-county level?<br>*(Je, kuna changamoto zozote zinazokumba uchanganuzi wa habari za ufuatiliaji wa magonjwa haya ya kitropiki yaliyo telekezwa katika kaunti hii ndogo?)*	Yes *(Ndio)* ☐ No *(La)* ☐ DK *(Sijui)* ☐ N/A *(Si Husika)* ☐
**Q31 (b).** If YES, what are some of these challenges?<br>*(Kama NDIO, tafadhali taja changamoto hizi)*	
**Q32.** In your own opinion, what can be done to improve PC-NTDs surveillance data analysis in this sub-county?<br>*(Kwa maoni yako binafsi, uchanganuzi wa habari za ufuatiliaji wa magonjwa haya ya kitropiki yaliyo telekezwa zinaweza kuimarishwa kwa njia zipi katika kaunti hii ndogo?)*	
**CASE INVESTIGATIONS & EPIDEMIC RESPONSE *(UCHUNGUZI WA KESI ZA MAGONJWA NA MWITIKIO WA MKURUPUKO WA MAGONJWA)***	
**Q33 (a).** Are PC-NTDs reported cases investigated at this sub-county level?<br>*(Je, kesi za magonjwa ya kitropiki yaliyo telekezwa yanachunguzwa katika kaunti hii ndogo?)*	Yes *(Ndio)* ☐ No *(La)* ☐ DK *(Sijui)* ☐ N/A *(Si Husika)* ☐
**Q33 (b).** If NO, why is this so?<br>*(Kama LA, sababu ni zipi?)*	
**Q33 (c).** If YES, describe case investigation for an PC-NTD reported to this sub-county level in the past one year.<br>*(Kama NDIO, eleza kwa kikamilifu uchunguzi uliyofanywa ya moja wapo ya kesi ya ugonjwa wa kitropiki iliyo telekezwa katika kaunti hii ndogo kwa mwaka uliopita?)*	
**Q33 (d).** What action/s followed the case investigation?<br>*(Je, ni hatua zipi zilifuatilia uchunguzi wa kesi hizi?)*	
**Q33 (e).** Was the PC-NTD case investigation guided by IDSR standard guidelines?<br>*(Je, uchunguzi huu ulielekezwa kutokana na mfumo maalum wa IDSR?)*	Yes *(Ndio)* ☐ No *(La)* ☐ DK *(Sijui)* ☐ N/A *(Si Husika)* ☐
**Q33 (f).** If NO, why is this so?<br>*(Kama LA, sababu ni zipi?)*	
**Q34 (a).** Is there a rapid response team that responds to PC-NTDs outbreaks in this sub-county?<br>*(Je, kuna timu maalum ya kuzingatia mwitikio wa mkurupuko wa magonjwa ya kitropiki yaliyo telekezwa katika kaunti hii ndogo?)*	Yes *(Ndio)* ☐ No *(La)* ☐ DK *(Sijui)* ☐ N/A *(Si Husika)* ☐
**Q34 (b).** Are there adequate supplies in this sub-county to respond to PC-NTDs outbreaks?	Yes *(Ndio)* ☐ No *(La)* ☐ DK *(Sijui)* ☐ N/A *(Si Husika)* ☐

(Je, kuna vifaa vya kutosha vya kuwezesha mwitikio wa mkurupuko wa magonjwa ya kitropiki yaliyo telekezwa katika kaunti hii ndogo?)	
**FEEDBACK *(MAONI)***	
**Q35 (a).** Does this sub-county receive feedback on PC-NTDs reports sent to the higher level?<br>*(Je, kaunti hii ndogo inapokea maoni kufuatilia ripoti za ufuatiliaji wa magonjwa ya kitropiki yaliyo telekezwa yaliyotumwa kuelekea kiwango cha juu?)*	Yes *(Ndio)* ☐ No *(La)* ☐ DK *(Sijui)* ☐ N/A *(Si Husika)* ☐
**Q35 (b).** If YES, how many PC-NTDs feedback reports has this sub-county received from the higher level in the past one year?<br>*(Kama NDIO, je kwa mwaka uliopita mulipokea ripoti ngapi za maoni katika kaunti hii ndogo kufuatilia kutuma ripoti za ufuatiliaji wa magonjwa ya kitropiki yaliyo telekezwa kwa kiwango cha juu?)*	☐ 1-2 *(Moja au Miwili)*<br>☐ 3 or more *(Tatu au Zaidi)*<br>☐ Don't Know *(Sijui)*<br>☐ N/A *(Si Husika)*
**Q36 (a).** Do you feel feedback from the higher level regarding PC-NTDs is important to this sub-county?<br>*(Je, unahisi ya kwamba maoni manazopokea kutoka kiwango cha juu kuhusiana na ufuatiliaji wa magonjwa ya kitropiki yaliyo telekezwa yana manufaa kwenyu katika kaunti hii ndogo?)*	Yes *(Ndio)* ☐ No *(La)* ☐ DK *(Sijui)* ☐ N/A *(Si Husika)* ☐
**Q36 (b).** If NO, why do you say so?<br>*(Kama LA, sababu ni zipi?)*	
**Q36 (c).** If YES, how can feedback from the higher level to this sub-county be improved?<br>*(Kama NDIO, je ripoti za maoni kutoka viwango vya juu vinaweza kuimarishwa na wasimamizi kwa njia zipi?)*	
**Q37 (a).** Do you conduct feedback meetings regarding PC-NTDs with health facilities reporting PC-NTDs surveillance data to this sub-county level?<br>*(Je, mufanya mikutano ya maoni na baadhi ya vituo vya afya vinavyo ripoti magonjwa haya ya kitropiki yaliyo telekezwa?)*	Yes *(Ndio)* ☐ No *(La)* ☐ DK *(Sijui)* ☐ N/A *(Si Husika)* ☐
**Q37 (b).** If YES, how many feedback meetings have been held in the past one year?<br>*(Kama NDIO, je mumeandaa mikutano ngapi ya maoni na baadhi ya vituo hivi vya afya kwa mwaka uliopita?)*	☐ None *(Hamna)*<br>☐ 1-2 *(Mara moja au miwili)*<br>☐ 3 or more (*Mara tatu au zaidi)*<br>☐ Don't Know *(Sijui)*<br>☐ N/A *(Si Husika)*
**Q37 (c).** If NO, why is this so?<br>*(Kama LA, sababu ni zipi?)*	
**Q38 (a).** Does this sub-county send written PC-NTDs feedback reports to the health facilities reporting to this level?<br>*(Je, kiwango hiki cha kaunti ndogo kinatuma ripoti za maoni kuhusiana na magonjwa ya kitropiki yaliyo*	Yes *(Ndio)* ☐ No *(La)* ☐ DK *(Sijui)* ☐ N/A *(Si Husika)* ☐

telekezwa kwa vituo vya afya vinavyo ripoti kwa kiwango hiki?	
**Q38 (b).** If NO, why is this so?<br>*(Kama LA, sababu ni zipi?)*	
**Q38 (c).** If YES, are the written feedback reports to the health facilities produced according to a standard guideline?<br>*(Kama NDIO, je ripoti hizi za maoni zinatengenezwa kwa mtindo maalum?)*	Yes *(Ndio)* ☐ No *(La)* ☐ DK *(Sijui)* ☐ N/A *(Si Husika)* ☐
**Q38 (d).** How many feedback reports regarding PC-NTDs has this sub-county disseminated to the health facilities in the past one year?<br>*(Je, kwa mwaka uliopita kiwango hiki kilisambaza ripoti ngapi za maoni kuhusu magonjwa ya kitropiki yaliyo telekezwa kuelekea kwa viwango vya vituo vya afya katika kaunti hii ndogo?)*	☐ None *(Hamna)*<br>☐ 1-2 *(Moja au Miwili)*<br>☐ 3 or more *(Tatu au Zaidi)*<br>☐ Don't Know *(Sijui)*<br>☐ N/A *(Si Husika)*
**Q38 (e).** Are there challenges in disseminating PC-NTDs feedback reports to the health facilities reporting to this sub-county level?<br>(*Je, kuna changamoto zozote zinazo kumba kusambazwa kwa ripoti za maoni kuhusu magonjwa ya kitropiki yaliyo telekezwa kuelekea kwa viwango vya vituo vya afya katika kaunti hii ndogo?)*	Yes *(Ndio)* ☐ No *(La)* ☐ DK *(Sijui)* ☐ N/A *(Si Husika)* ☐
**Q38 (f).** If YES, what are some of these challenges?<br>*(Kama NDIO, changamoto hizi ni zipi?)*	
**Q39.** In your own opinion, how can feedback coming from the higher level regarding reported PC-NTDs surveillance data be improved?<br>*(Je, kwa maoni yako binafsi viwango vya juu zaidi vinaweza kuboresha ki vipi kusambaza maoni kuhusu ufuatiliaji wa magonjwa ya kitropiki yaliyo telekezwa zilizo ripotiwa na kiwango hiki?)*	
**Q40.** In your own opinion, how can feedback to health facilities reporting PC-NTDs surveillance data to this level be improved?<br>*(Je, kwa maoni yako binafsi kiwango hiki cha kaunti ndogo kinaweza kuboresha ki vipi kusambaza maoni kuhusu ufuatiliaji wa magonjwa ya kitropiki yaliyo telekezwa kueleka kwa viwango vya vituo vya afya vinavoripoti kesi hizi?)*	
**STANDARDS AND GUIDELINES (UBORESHAJI NA VIELEKEZI)**	
**Q41 (a).** Are IDSR guidelines and manuals available in this sub-county?<br>*(Je vielekezi vya ufuatiliaji wa magonjwa kupitia kwa mfumo wa IDSR vinapatikana katika kaunti hii ndogo?)*	
**Q41 (b).** If NO, why is this so?<br>*(Kama LA, sababu ni zipi?)*	

**Q42 (a).** Do the IDSR manuals guide PC-NTDs surveillance activities in this sub-county?<br>*(Je vielekezi hivi vya IDSR vinaelekeza shughuli za ufuatiliaji wa magonjwa ya kitropiki yaliyo telekezwa katika kaunti hii ndogo?)*	Yes *(Ndio)* ☐ No *(La)* ☐ DK *(Sijui)* ☐ N/A *(Si Husika)* ☐
**Q42 (b).** If NO, why is this so?<br>*(Kama LA, sababu ni zipi?)*	
**Q43 (a).** Do you have standard guidelines for PC-NTDs case detection, confirmation, reporting and data analysis in this sub-county?<br>*(Je, muna vielekezo maalum za ugunduzi, kuthibiti, kuripoti na uchanganuzi wa kesi za magonjwa ya kitropiki yaliyo telekezwa katika kaunti hii ndogo?)*	Yes *(Ndio)* ☐ No *(La)* ☐ DK *(Sijui)* ☐ N/A *(Si Husika)* ☐
**Q43 (b).** If NO, why is this so?<br>*(Kama LA, sababu ni zipi?)*	
**SUPERVISION *(USIMAMIZI)***	
**Q44 (a).** Do you receive regular supervisory visits from the higher level in this sub-county?<br>*(Je munapokea usimamizi wa mara kwa mara katika kaunti hii ndogo kutoka kwa maafisa wa viwango vya juu?)*	Yes *(Ndio)* ☐ No *(La)* ☐ DK *(Sijui)* ☐ N/A *(Si Husika)* ☐
**Q44 (b).** If NO, why do think this is so?<br>*(Kama LA, sababu ni zipi?)*	
**Q44 (c).** If YES, how often have you received supervisory visits from the higher level in the past one year?<br>*(Kama NDIO, je kwa mwaka uliopita mulipokea ziara za usimamizi mara ngapi kutoka kwa viwango vya juu?)*	☐ At least once *(Mara moja)*<br>☐ At least twice *(Mara mbili)*<br>☐ More than twice *(Zaidi ya mara mbili)*<br>☐ Quarterly *(Mwisho wa robo wa kila mwaka)*<br>☐ N/A
**Q45 (a).** In the last supervisory visits, were surveillance activities in this sub-county reviewed?<br>(*Je, masuala ya shughuli za ufuatiliaji wa magonjwa yalikaguliwa katika ziara za mwisho za usimamizi katika kiwango hiki cha kaunti ndogo?)*	Yes *(Ndio)* ☐ No *(La)* ☐ DK *(Sijui)* ☐ N/A *(Si Husika)* ☐
**Q45 (b).** If NO, why was this so?<br>*(Kama LA, sababu ni zipi?)*	
**Q46 (a).** In the last supervisory visits, were PC-NTDs surveillance activities reviewed or discussed?<br>*(Je, suala za ufuatiliaji wa magonjwa ya kitropiki yaliyo telekezwa zilikaguliwa katika ziara za mwisho za usimamizi?)*	Yes *(Ndio)* ☐ No *(La)* ☐ DK *(Sijui)* ☐ N/A *(Si Husika)* ☐
**Q46 (b).** If NO, why was this so?<br>*(Kama LA, sababu ni zipi?)*	
**Q47 (a).** In the last supervisory visits, did you receive a feedback report on PC-NTDs surveillance activities performance in this sub-county?	Yes *(Ndio)* ☐ No *(La)* ☐ DK *(Sijui)* ☐ N/A *(Si Husika)* ☐

(Je, mulipokea ripoti maalum ya maoni kutokana na ufuatiliaji wa magonjwa haya ya kitropiki yaliyo telekezwa baada ya ziara za mwisho za usimamizi katika kiwango hiki cha kaunti ndogo?)	
**Q47 (b).** If NO, why was this so?<br>*(Kama LA, sababu ni zipi?)*	
**Q48 (a).** In the last supervisory visits, were there any recommendations made concerning PC-NTDs surveillance in this sub-county in the last supervisory visits?<br>*(Je, kulikuwepo na mapendekezo zozote kuhusu ufuatiliaji wa magonjwa ya kitropiki yaliyo telekezwa wakati wa ziara za mwisho za usimamizi katika kiwango hiki cha kaunti ndogo?)*	Yes *(Ndio)* ☐ No *(La)* ☐ DK *(Sijui)* ☐ N/A *(Si Husika)* ☐
**Q48 (b).** If YES, what were some of the recommendations made?<br>*(Kama NDIO, je mapendekezo hizo zilikuwa zipi?)*	
**Q48 (c).** In the last supervisory visits, were there any follow-ups on recommendations made from a previous visit?<br>*(Je, kulikuwepo na ufuatiliaji wowote wa mapendekezo yaliyotolewa kuhusu ufuatiliaji wa magonjwa ya kitropiki yaliyo telekezwa wakati wa ziara za mwisho za usimamizi katika kiwango hiki cha kaunti ndogo?)*	Yes *(Ndio)* ☐ No *(La)* ☐ DK *(Sijui)* ☐ N/A *(Si Husika)* ☐
**Q48 (d).** If YES, what did the follow-up entail?<br>*(Kama NDIO, je ufuatiliaji huu uligusia nini haswa?)*	
**Q49 (a).** Are supervisory visits regarding disease surveillance activities conducted in the health facilities reporting to this sub county level?<br>*(Je, kiwango hiki cha kaunti ndogo kina tekeleza ziara za usimamizi kuhusu shughuli za ufuatiliaji wa magonjwa katika viwango vya vituo vya afya vinavyoripoti katika kiwango hiki?)*	Yes *(Ndio)* ☐ No *(La)* ☐ DK *(Sijui)* ☐ N/A *(Si Husika)* ☐
**Q49 (b).** If NO, why is this so?<br>*(Kama LA, sababu ni zipi?)*	
**Q49 (c).** If YES, how often were supervisory visits regarding disease surveillance conducted in the health facilities reporting to this level in the past one year?<br>*(Kama NDIO, je kiwango hiki kilitekeleza kwa mara ngapi ziara hizi za usimamizi wa shughuli za ufuatiliaji wa magonjwa katika viwango vya vituo vya afya?)*	☐ At least once *(Mara moja)*<br>☐ At least twice *(Mara mbili)*<br>☐ More than twice *(Zaidi ya mara mbili)*<br>☐ Quarterly *(Mwisho wa robo wa kila mwaka)*<br>☐ N/A
**Q49 (d).** What is the recommended number of supervisory visits to the health facilities in a period of one year?<br>*(Je, inapendekezwa kiwango hiki cha kaunti ndogo kitimize shughuli za ziara za usimamizi katika viwango vya vituo vya afya kwa mara ngapi kwa mwaka?)*	☐ At least once *(Mara moja)*<br>☐ At least twice *(Mara mbili)*<br>☐ More than twice *(Zaidi ya mara mbili)*<br>☐ Quarterly *(Mwisho wa robo wa kila mwaka)*<br>☐ N/A

**Q50 (a).** In the last supervisory visits to the health facilities reporting to this level, were PC-NTDs surveillance data reviewed?<br>*(Katika ziara za mwisho za usimamizi katika viwango vya vituo vya afya, je shughuli za ufuatiliaji wa magonjwa ya kitropiki yaliyo telekezwa zilikaguliwa?)*	Yes *(Ndio)* ☐ No *(La)* ☐ DK *(Sijui)* ☐ N/A *(Si Husika)* ☐
**Q50 (b).** If NO, why was this so?<br>*(Kama LA, sababu ni zipi?)*	
**Q50 (c).** If YES, was a supervision feedback report on PC-NTDs surveillance activities performance sent to the health facilities?<br>*(Je mulipokeza viwango vya vituo vya afya na ripoti maalum kufuatia ziara za mwisho za usimamizi wa shughuli za ufuatiliaji wa magonjwa ya kitropiki yaliyo telekezwa?)*	Yes *(Ndio)* ☐ No *(La)* ☐ DK *(Sijui)* ☐ N/A *(Si Husika)* ☐
**Q50 (d).** If NO, why was this so?<br>*(Kama LA, sababu ni zipi?)*	
**Q51 (a).** Is there a schedule/plan for conducing future supervisory visits in the health facilities reporting to this sub-county level?<br>*(Je, katika kiwago hiki cha kaunti ndogo kuna ratiba maalum ya kutekeleza ziara za usimamizi katika viwango vya vituo vya afya kwa siku zijazo?)*	Yes *(Ndio)* ☐ No *(La)* ☐ DK *(Sijui)* ☐ N/A *(Si Husika)* ☐
**Q51 (b).** If NO, why is this so?<br>*(Kama LA, sababu ni zipi?)*	
**Q52 (a).** Do you experience challenges conducting supervisory activities in the health facilities level?<br>*(Je, munapata changamoto zozote munapo tekeleza ziara za usimamizi katika viwango vya vituo vya afya?)*	Yes *(Ndio)* ☐ No *(La)* ☐ DK *(Sijui)* ☐ N/A *(Si Husika)* ☐
**Q52 (b).** If YES, what are some of these challenges?<br>*(Kama NDIO, je changamoto hizi ni zipi?)*	
**Q53.** In your own opinion, how can supervision of PC-NTDs surveillance activities from the higher level to this sub-county be improved?<br>*(Je, kwa maoni yako binafsi, viwango vya juu zaidi vinaweza kuboresha ki vipi ziara za usimamizi wa shughuli za ufuatiliaji wa magonjwa ya kitropiki yaliyo telekezwa katika kiwango hiki cha kaunti ndogo?)*	
**Q54.** In your own opinion, how can supervision of PC-NTDs surveillance activities in the health facilities by this sub-county be improved?<br>*(Je, kwa maoni yako binafsi, kiwango hiki kinaweza kuboresha ki vipi ziara za usimamizi wa shughuli za ufuatiliaji wa magonjwa ya kitropiki yaliyo telekezwa katika viwango vya vituo vya afya?)*	
**TRAINING *(MAFUNZO)***	
**Q55 (a).** In your basic training were you trained on disease surveillance?	Yes *(Ndio)* ☐ No *(La)* ☐ DK *(Sijui)* ☐ N/A *(Si Husika)* ☐

(Je, katika elimu yako ya msingi ulipata mafunzo ya ufuatiliaji wa magonjwa?)	
**Q55 (b).** If NO, why was this so?<br>*(Kama LA, sababu ni zipi?)*	
**Q56 (a).** Do you feel your basic training is sufficient for you to adequately supervise disease surveillance activities in this sub county?	Yes *(Ndio)* ☐ No *(La)* ☐ DK *(Sijui)* ☐ N/A *(Si Husika)* ☐
**Q56 (b).** If NO, why do you say so?<br>*(Kama LA, sababu ni zipi?)*	
**Q57 (a).** Is your basic training applicable to PC-NTDs surveillance in this sub-county?<br>*(Je, unahisi mafunzo yako ya msingi uliyopata yalikuwa ya kutosha kutimiza ufuatiliaji wa magonjwa katika kiwango hiki cha kaunti ndogo?)*	Yes *(Ndio)* ☐ No *(La)* ☐ DK *(Sijui)* ☐ N/A *(Si Husika)* ☐
**Q57 (b).** If NO, why do you say so?<br>*(Kama LA, sababu ni zipi?)*	
**Q58 (a).** While working in your current designation have you received any post basic training on disease surveillance in the past one year?<br>*(Je, kwa uajibu wako kikazi katika kiwango hiki umepata mafunzo zaidi maalum kuhusiana na ufuatiliaji wa magonjwa kando na elimu yako ya msingi kwa mwaka uliopita?)*	Yes *(Ndio)* ☐ No *(La)* ☐ DK *(Sijui)* ☐ N/A *(Si Husika)* ☐
**Q58 (b).** If YES *(Kama NDIO),*<br><br>When were you last trained *(Ulipata mafunzo hayo ya mwisho lini?)*<br><br>Where was the training *(Ulipata mafunzo hayo wapi?)*<br><br>Who facilitated the training *(Nani aliwezesha mafunzo hayo?)*<br><br>What was the duration of the training *(Mafunzo hayo yalikua kwa muda gani?)*	…………………………………………………..☐ N/A<br><br>…………………………………………………..☐ N/A<br><br>…………………………………………………..☐ N/A<br><br>…………………………………………………..☐ N/A
**Q58 (c).** Which specific elements of disease surveillance and response elements were covered in your last post-basic training?<br>*(Je mafunzo hayo yaliangazia mambo yapi haswa kuhusu ufuatiliaji wa magonjwa?)*	☐ Case Detection *(Ugunduzi wa kesi za magonjwa)*<br>☐ Case Registration *(Usajili wa kesi za magonjwa)*<br>☐ Case Confirmation *(Uthibitisho wa kesi za magonjwa)*<br>☐ Reporting *(Kuripoti kesi za magonjwa)*<br>☐ Data Analysis *(Uchanganuzi wa kesi za magonjwa)*<br>☐ Outbreak Investigation *(Uchunguzi wa kuzuka kwa magonjwa)*<br>☐ Response and Control *(Uajibu na udhibiti wa mgonjwa)*<br>☐ Others *(Mada zinginezo)*:…………………………….<br>☐ DK *(Sijui)*<br>☐ N/A *(Si Husika)*

**Q58 (d).** In your last post-basic training, was disease surveillance and response specific to PC-NTDs covered?<br>*(Je, mafunzo hayo ya mwisho yaliangazia ufuatiliaji wa magonjwa ya kitropiki yaliyo telekezwa?)*	Yes *(Ndio)* ☐ No *(La)* ☐ DK *(Sijui)* ☐ N/A *(Si Husika)* ☐
**Q58 (e).** If YES, which specific elements regarding PC-NTDs surveillance and response were covered in the training?<br>*(Kama NDIO, je ni mambo yapi haswa yaliangaziwa kuhusiana na ufuatiliaji wa magonjwa ya kitropiki yaliyo telekezwa?)*	☐ Case Detection *(Ugunduzi wa kesi za magonjwa)*<br>☐ Case Registration *(Usajili wa kesi za magonjwa)*<br>☐ Case Confirmation *(Uthibitisho wa kesi za magonjwa)*<br>☐ Reporting *(Kuripoti kesi za magonjwa)*<br>☐ Data Analysis *(Uchanganuzi wa kesi za magonjwa)*<br>☐ Outbreak Investigation *(Uchunguzi wa kuzuka kwa magonjwa)*<br>☐ Response and Control *(Uajibu na udhibiti wa mgonjwa)*<br>☐ Others *(Mada zinginezo)*:…………………………….<br>☐ DK *(Sijui)*<br>☐ N/A *(Si Husika)*
**Q58 (f).** If NO, would you be interested in being trained specifically on PC-NTDs surveillance and response?<br>*(Kama LA, je unania ya mafunzo inayoangazia ufuatiliaji wa magonjwa ya kitropiki yaliyo telekezwa?)*	Yes *(Ndio)* ☐ No *(La)* ☐ DK *(Sijui)* ☐ N/A *(Si Husika)* ☐
**Q58 (g).** If YES, which specific aspect/s regarding PC-NTDs surveillance and response would you like the training to focus on amongst those mentioned in this interview?<br>*(Kama NDIO, je ni mambo yapi haswa ungependa mafunzo hayo ya angazie kuhusiana na ufuatiliaji wa magonjwa ya kitropiki yaliyo telekezwa?)*	☐ Case Detection *(Ugunduzi wa kesi za magonjwa)*<br>☐ Case Registration *(Usajili wa kesi za magonjwa)*<br>☐ Case Confirmation *(Uthibitisho wa kesi za magonjwa)*<br>☐ Reporting *(Kuripoti kesi za magonjwa)*<br>☐ Data Analysis *(Uchanganuzi wa kesi za magonjwa)*<br>☐ Outbreak Investigation *(Uchunguzi wa kuzuka kwa magonjwa)*<br>☐ Response and Control *(Uajibu na udhibiti wa mgonjwa)*<br>☐ Others *(Mada zinginezo)*:…………………………….<br>☐ DK (Sijui)<br>☐ N/A
**Q59 (a).** Are there any challenges facing post-basic training of health personnel in this sub-county?<br>*(Je, kuna chamgamoto zozote zinazokumba maandalizi ya mafunzo ya wafanyakazi wa afya katika kiwango hiki cha kaunti ndogo?)*	Yes *(Ndio)* ☐ No *(La)* ☐ DK *(Sijui)* ☐ N/A *(Si Husika)* ☐
**Q59 (b).** If YES, what are some of these challenges?<br>*(Kama NDIO, je changamoto hizi ni zipi?)*	
**Q60.** In your own opinion, how can post-basic training on PC-NTDs surveillance and response be improved in this sub-county?<br>*(Kwa maoni yako binafsi, je mafunzo yanayoangazia ufuatiliaji wa magonjwa ya kitropiki yaliyo telekezwa*	

zinaeza kuboreshwa ki vipi katika kiwango hiki cha kaunti ndogo?)	
**OPPORTUNITIES FOR IMPROVEMENT** ***(NAFASI ZA UBORESHAJI WA UFUATILIAJI WA MAGONJWA)***	
**Q61 (a).** Are you satisfied with PC-NTDs surveillance and response activities within the IDSR system in this sub-county as it is? *(Je, umeridhishwa na shughuli za ufuatiliaji wa magonjwa ya kitropiki yaliyo telekezwa katika kiwango hiki cha kaunti ndogo vile zilivyo?)*	Yes *(Ndio)* ☐ No *(La)* ☐ DK *(Sijui)* ☐ N/A *(Si Husika)* ☐
**Q61 (b).** If NO, what are the main challenges facing PC-NTDs surveillance and response activities within the IDSR system in this sub-county? *(Kama LA, ni changamoto zipi kuu zinazo kumba shughuli za ufuatiliaji wa magonjwa ya kitropiki yaliyo telekezwa katika kiwango hiki cha kaunti ndogo?)*	
**Q62.** In your own opinion, what can be done to improve PC-NTDs surveillance and response in this sub-county? *(Kwa maoni yako binafsi, je shughuli hizi za ufuatiliaji wa magonjwa ya kitropiki yaliyo telekezwa zinaweza kuboreshwa kwa njia zipi katika kiwango hiki cha kaunti ndogo?)*	
**RESOURCES** ***(RASILIMALI)***	
Availability of electricity *(Uwepo wa umeme)*	Yes *(Ndio)* ☐ No *(La)* ☐
Availability of computers *(Uwepo wa kompyuta)*	Yes *(Ndio)* ☐ No *(La)* ☐
Availability of telephones/mobile services *(Uwepo wa simu)*	Yes *(Ndio)* ☐ No *(La)* ☐
Availability of internet services *(Uwepo wa mtandao)*	Yes *(Ndio)* ☐ No *(La)* ☐
Availability of PC-NTD posters *(Uwepo wa tangazo za ukutani za magonjwa ya kitropiki yaliyo telekezwa zinaweza)*	Yes *(Ndio)* ☐ No *(La)* ☐

COUNTY LEVEL QUESTIONNAIRE *(DODOSO LA KIWANGO CHA KAUNTI)*

<table>
<tr><td>GENERAL INFORMATION
(Habari kwa ujumla)</td><td colspan="2">Please mark (X) in the appropriate box
(Tafadhali weka alama ya tiki (√) katika sehemu inayofaa zaidi)</td></tr>
<tr><td>Region
(Eneo)</td><td colspan="2">……………………………………… County
(Kaunti)</td></tr>
<tr><td rowspan="2">Demographic Characteristics
(Demografia ya mhojiwa)</td><td>Age (Umri)</td><td>☐ 18-30 ☐ 31-40
☐ 41-50 ☐ >50</td></tr>
<tr><td>Sex (Jinsia)</td><td>Male (Kiume) ☐ Female (Kike) ☐</td></tr>
<tr><td>Q1. What is your current designation?
(Wajibu wa mfanyakazi wa afya)</td><td colspan="2">………………………………………………</td></tr>
<tr><td>Number of years worked in your current designation
(Miaka yako kikazi katika uajibu huu?)</td><td colspan="2">☐ Less than a year (Chini ya mwaka 1)
☐ 1-2 years (Kati ya mwaka 1-2)
☐ 2-3 years (Kati ya miaka 2-3)
☐ 3-5 years (Kati ya miaka 3-5)
☐ More than 5years (Zaidi ya miaka 5)</td></tr>
<tr><td>Q2. What is your highest level of education?
(Kiwango chako cha juu zaidi katika masomo?)</td><td colspan="2">☐ PhD (Shahada ya uzamifu)
☐ Masters (Shahada ya uzamili)
☐ Degree (Shahada)
☐ Diploma (Stashahada)
☐ Certificate (Astashahada)</td></tr>
<tr><td>DISEASE SURVEILLANCE (UFUATILIAJI WA MAGONJWA)</td><td colspan="2"></td></tr>
<tr><td>Q3. What do you understand by the term disease surveillance?
(Je unaelewa vipi ufuatiliaji wa magonjwa?)</td><td colspan="2"></td></tr>
<tr><td>Q4. What do you understand by the term neglected tropical diseases?
(Je, unaelewa ki vipi istilahi ya magonjwa ya kitropiki yaliyo telekezwa?)</td><td colspan="2"></td></tr>
<tr><td>Q5 (a). Are you aware of any neglected tropical diseases in this county?
(Je unafahamu magonjwa haya ya kitropiki yaliyo telekezwa katika kaunti hii ndogo?)</td><td colspan="2">☐ Yes (Ndio) ☐ No (La) ☐ DK (Sijui)</td></tr>
<tr><td>Q5 (b). If YES, which are the preventive chemotherapy targeted neglected tropical diseases (PC-NTDs) common in this county?
(Kama NDIO, je magonjwa haya ya kitropiki yaliyo telekezwa ambazo ni za kawaida katika kaunti hii ndogo ni zipi?)</td><td colspan="2">☐ Lymphatic Filariasis (Matende)
☐ Soil Transmitted Helminths (Minyoo)
☐ Trachoma (Trakoma)
☐ Schistosomiasis (Kichocho)</td></tr>
</table>

	☐ Others *(Magonjwa zinginezo)*: ……………………….... ☐ Don't Know *(Sijui)* ☐ N/A *(Si Husika)*
**Q5 (c).** Which of the PC-NTDs mentioned above are commonly reported in this county? *(Je ni magonjwa yapi ya kitropiki yaliyo telekezwa ulizo taja zinaripotiwa katika kaunti hii ndogo?)*	☐ Lymphatic Filariasis *(Matende)* ☐ Soil Transmitted Helminths *(Minyoo)* ☐ Trachoma *(Trakoma)* ☐ Schistosomiasis *(Kichocho)* ☐ Others *(Magonjwa zinginezo)*: ……………………….... ☐ Don't Know *(Sijui)* ☐ N/A *(Si Husika)*
**Q6 (a).** Is there a functional disease surveillance system present in this county? *(Je kuna mfumo maalum ya ufuatiliaji wa magonjwa katika kaunti hii ndogo?)*	Yes *(Ndio)* ☐ No *(La)* ☐ DK *(Sijui)* ☐ N/A *(Si Husika)* ☐
**Q6 (b).** If NO, why do you say so? *(Kama LA, tafadhali nipe sababu zako?)*	
**Q7 (a).** Are you aware of the integrated disease surveillance and response (IDSR) system? *(Je unaelewa vipi mfumo wa IDSR?)*	Yes *(Ndio)* ☐ No *(La)* ☐ DK *(Sijui)* ☐ N/A *(Si Husika)* ☐
**Q7 (b).** If YES, are any of the above mentioned PC-NTDs reported through the IDSR system? *(Kama NDIO, je magonjwa ya kitropiki yaliyo telekezwa zinazoripotiwa kupitia mfumo huu?)*	Yes *(Ndio)* ☐ No *(La)* ☐ DK *(Sijui)* ☐ N/A *(Si Husika)* ☐
**Q7 (c).** If NO, why is this so? *(Kama LA, sababu ni zipi?)*	
**Q7 (d).** If YES, specifically which PC-NTDs are reported through this system? *(Kama NDIO, je ni magonjwa yapi ya kitropiki yaliyo telekezwa zinazoripotiwa kupitia mfumo huu?)*	☐ Lymphatic Filariasis *(Matende)* ☐ Soil Transmitted Helminths *(Minyoo)* ☐ Trachoma *(Trakoma)* ☐ Schistosomiasis *(Kichocho)* ☐ Others *(Magonjwa zinginezo)*: ……………………….... ☐ Don't Know *(Sijui)* ☐ N/A *(Si Husika)*
**Q8 (a).** In your own opinion, are PC-NTDs surveillance data adequately reported within the IDSR system in this county? *(Kwa maoni yako binafsi, je magonjwa haya ya kitropiki yaliyo telekezwa zinaripotiwa kwa njia mwafaka kupitia mfumo wa IDSR katika kaunti hii ndogo?)*	Yes *(Ndio)* ☐ No *(La)* ☐ DK *(Sijui)* ☐ N/A *(Si Husika)* ☐
**Q8 (b).** If NO, why do you say so? *(Kama LA, sababu ni zipi?)*	

**SURVEILLANCE MANUALS & CASE DEFINITIONS *(MIONGOZO ZA UFUATILIAJI NA KESI ZA MAGONJWA)***	
**Q9 (a).** Is there a manual for disease surveillance present in this county? *(Je, kuna miongozo maalum za ufuatiliaji wa magonjwa katika kaunti hii ndogo?)*	Yes *(Ndio)* ☐ No *(La)* ☐ DK *(Sijui)* ☐ N/A *(Si Husika)* ☐
**Q9 (b).** If NO, why is this so? *(Kama LA, sababu ni zipi?)*	
**Q9 (c).** If YES, is the disease surveillance manual in use of a recent version? *(Kama NDIO, je miongozo hizi za ufuatiliaji wa magonjwa ni za hivi karibuni?)*	Yes *(Ndio)* ☐ No *(La)* ☐ DK *(Sijui)* ☐ N/A *(Si Husika)* ☐
**Q9 (d).** Is the manual useful in guiding disease surveillance activities at this county level? *(Je, miongozo hizi zinaelekeza vyema ufuatiliaji wa magonjwa katika kaunti hii ndogo?)*	Yes *(Ndio)* ☐ No *(La)* ☐ DK *(Sijui)* ☐ N/A *(Si Husika)* ☐
**Q9 (e).** If NO, why is this so? *(Kama LA, sababu ni zipi?)*	
**Q9 (f).** Is the disease surveillance manual easy to use? *(Je, miongozo hizi za ufuatiliaji wa magonjwa ni rahisi kutumia?)*	Yes *(Ndio)* ☐ No *(La)* ☐ DK *(Sijui)* ☐ N/A *(Si Husika)* ☐
**Q9 (g).** If NO, why do you say so? *(Kama LA, sababu ni zipi?)*	
**Q9 (h).** Does the manual specifically guide PC-NTDs surveillance activities in this county? *(Je, miongozo hizi zinaelekeza ufuatiliaji wa magonjwa ya kitropiki yaliyo telekezwa katika kaunti hii ndogo?)*	Yes *(Ndio)* ☐ No *(La)* ☐ DK *(Sijui)* ☐ N/A *(Si Husika)* ☐
**Q9 (i).** If NO, why do you think so? *(Kama LA, sababu ni zipi?)*	
**Q10 (a).** Are PC-NTDs case definitions available in this county? *(Je, miongozo za kutambua kesi za magonjwa ya kitropiki yaliyo telekezwa zinapatikana kwa urahisi katika kaunti hii ndogo?)*	Yes *(Ndio)* ☐ No *(La)* ☐ DK *(Sijui)* ☐ N/A *(Si Husika)* ☐
**Q10 (b).** If NO, why is this so? *(Kama LA, sababu ni zipi?)*	
**Q10 (c).** If YES, describe the availability of case definitions for the following PC-NTDs: *(Kama NDIO, tafadhali eleza kama miongozo za kutambua kesi zifuatazo za magonjwa ya kitropiki yaliyo telekezwa zinapatikana kwa urahisi*?)	
1. Lymphatic Filariasis *(Matende)*	Yes *(Ndio)* ☐ No *(La)* ☐ DK *(Sijui)* ☐ N/A *(Si Husika)* ☐
2. Soil Transmitted Helminths *(Minyoo)*	Yes *(Ndio)* ☐ No *(La)* ☐ DK *(Sijui)* ☐ N/A *(Si Husika)* ☐
3. Trachoma *(Trakoma)*	Yes *(Ndio)* ☐ No *(La)* ☐ DK *(Sijui)* ☐ N/A *(Si Husika)* ☐

4. Schistosomiasis *(Kichocho)*	Yes *(Ndio)* ☐ No *(La)* ☐ DK *(Sijui)* ☐ N/A *(Si Husika)* ☐
5. Other PC-NTDs: ………………………………….. *(Magonjwa zinginezo)*	Yes *(Ndio)* ☐ No *(La)* ☐ DK *(Sijui)* ☐ N/A *(Si Husika)* ☐
**CASE CONFIRMATION *(UTHIBITISHO WA KESI ZA MAGONJWA)***	
**Q11 (a).** Are there an adequate number of functional laboratories in this county? *(Je kuna maabara za kutosha zinaozofanya kazi katika kaunti hii ndogo?)*	Yes *(Ndio)* ☐ No *(La)* ☐ DK *(Sijui)* ☐ N/A *(Si Husika)* ☐
**Q11 (b).** If NO, why do you say so? *(Kama LA, sababu ni zipi?)*	
**Q11 (c).** If YES, are the laboratories adequately equipped to confirm cases of PC-NTDs? *(Kama NDIO, je maabara haya yanavifaa vya kutosha vya kudhibitisha magonjwa ya kitropiki yaliyo telekezwa?)*	Yes *(Ndio)* ☐ No *(La)* ☐ DK *(Sijui)* ☐ N/A *(Si Husika)* ☐
**Q11 (d).** If NO, why do you say so? *(Kama LA, sababu ni zipi?)*	
**Q12 (a).** Does this county have guidelines for specimen collection, handling, storage and transportation to the next higher level? (*Je kaunti hii ndogo ina miongozo za ukusanyaji, utunzaji, uhifaji na usafirishaji wa sampuli za magonjwa kuelekezwa katika maabara za juu zaidi?)*	Yes *(Ndio)* ☐ No *(La)* ☐ DK *(Sijui)* ☐ N/A *(Si Husika)* ☐
**Q12 (b).** If NO, why is this so? *(Kama LA, sababu ni zipi?)*	
**Q13 (a).** Are there any challenges facing PC-NTDs specimen collection, storage and transportation in this county? *(Je, kuna changamoto zozote zinazokumba ukusanyaji, utunzaji, uhifaji na usafirishaji wa sampuli za magonjwa za kitropiki yaliyo telekezwa katika kaunti hii ndogo?)*	Yes *(Ndio)* ☐ No *(La)* ☐ DK *(Sijui)* ☐ N/A *(Si Husika)* ☐
**Q13 (b).** If YES, what are some of these challenges? *(Kama NDIO, changamoto hizi ni zipi?)*	
**Q14 (a).** Does this county have the capacity to transport PC-NTDs specimens to a higher level laboratory? *(Je kaunti hii ndogo ina uwezo wa kusafirisha sampuli za magonjwa ya kitropiki yaliyo telekezwa kwa maabara za juu zaidi?)*	Yes *(Ndio)* ☐ No *(La)* ☐ DK *(Sijui)* ☐ N/A *(Si Husika)* ☐
**Q14 (b).** Do you make follow-ups on PC-NTDs specimens at this level? *(Je, munafuatilia matokeo ya sampuli hizi za magonjwa haya ya kitropiki yaliyo telekezwa katika kaunti hii ndogo?)*	Yes *(Ndio)* ☐ No *(La)* ☐ DK *(Sijui)* ☐ N/A *(Si Husika)* ☐
**Q14 (c).** If NO, why is this so? *(Kama LA, sababu ni zipi?)*	

**Q14 (d).** Do you receive reports for PC-NTDs specimens sent to the higher level laboratory?<br>*(Je, munapokea matokeo ya sampuli hizi za magonjwa ya kitropiki yaliyo telekezwa kutoka kwa maabara za juu zaidi?)*	Yes *(Ndio)* ☐ No *(La)* ☐ DK *(Sijui)* ☐ N/A *(Si Husika)* ☐
**Q14 (e).** If NO, why is this so?<br>*(Kama LA, sababu ni zipi?)*	
**Q14 (f).** Are the reports received from the higher level laboratory complete and elaborate?<br>*(Je, matokeo ya sampuli hizi kutoka kwa maabara za juu zaidi ni kamili na zenye ufafanuzi tosha?)*	Yes *(Ndio)* ☐ No *(La)* ☐ DK *(Sijui)* ☐ N/A *(Si Husika)* ☐
**Q14 (g).** If NO, why do you say so?<br>*(Kama LA, sababu ni zipi?)*	
**Q14 (h).** Are the reports received from the higher-level laboratory timely?<br>*(Je, matokeo ya sampuli hizi kutoka kwa maabara za juu zaidi munazipata kwa wakati unaofaa?*	Yes *(Ndio)* ☐ No *(La)* ☐ DK *(Sijui)* ☐ N/A *(Si Husika)* ☐
**Q14 (i).** If NO, why do you say so?<br>*(Kama LA, sababu ni zipi?)*	
**SURVEILLANCE DATA REPORTING *(KURIPOTI KWA HABARI ZA UFUATILIAJI WA MAGONJWA)***	
**Q15.** Which form of reporting is used to report surveillance data to the next level in this county?<br>*(Je, ni mfumo upi unaotumika kuripoti habari za ufuatiliaji wa magonjwa kuelekea katika kiwango cha juu zaidi?)*	☐ Paper-based *(Kwa njia za karatasi)*<br>☐ Electronic-based (*Kwa njia za kielektroniki)*<br>☐ Both Paper and Electronic-based (*Kwa njia za karatasi na za kielektroniki)*<br>☐ DK *(Sijui)*<br>☐ N/A *(Si Husika)*
**Q16 (a).** Has this county lacked disease surveillance reporting forms at any given time in the past six months?<br>*(Je, mushawahi kumbwa na ukosefu wa fomu hizi za kuripoti magonjwa kwa wakati wowote kwa muda wa miezi sita zilizopita?)*	Yes *(Ndio)* ☐ No *(La)* ☐ DK *(Sijui)* ☐ N/A *(Si Husika)* ☐
**Q16 (b).** If YES, why was this so?<br>(*Kama NDIO, sababu ni zipi?)*	
**Q17 (a).** Are the surveillance reporting forms adequate for reporting PC-NTDs?<br>*(Je fomu hizi zinautoaji tosha wa kuripoti magonjwa haya ya kitropiki yaliyo telekezwa)?*	Yes *(Ndio)* ☐ No *(La)* ☐ DK *(Sijui)* ☐ N/A *(Si Husika)* ☐
**Q17 (b).** If NO, why do you say so?<br>*(Kama LA, sababu ni zipi?)*	
**Q18 (a).** Are the surveillance reporting forms easy to complete?<br>*(Je, ni rahisi kukamilisha fomu hizi za kuripotia magonjwa?)*	Yes *(Ndio)* ☐ No *(La)* ☐ DK *(Sijui)* ☐ N/A *(Si Husika)* ☐
**Q18 (b).** If NO, why do you say so?	

(Kama LA, sababu ni zipi?)	
**Q19.** How often are PC-NTDs surveillance reports prepared and sent to the county level from this county?<br>*(Je, ripoti za ufuatiliaji wa magonjwa ya kitropiki yaliyo telekezwa kueleka kiwango cha kaunti zinatumwa kwa kipindi ipi mara kwa mara?)*	☐ Weekly *(Kwa kila wiki)*<br>☐ Monthly *(Kwa kila mwezi)*<br>☐ Quarterly *(Kwa kila robo wa mwisho wa mwezi)*<br>☐ Bi-annually *(Mara mbili kwa mwaka)*<br>☐ Annually *(Mara moja kwa mwaka)*<br>☐ DK *(Sijui)*<br>☐ N/A *(Si Husika)*
**Q20 (a).** Are there deadlines for sending PC-NTDs surveillance reports to the county level?<br>*(Je kuna tarehe za mwisho za kutuma ripoti za ufuatiliaji wa magonjwa ya kitropiki yaliyo telekezwa kueleka kiwango cha kaunti?)*	Yes *(Ndio)* ☐ No *(La)* ☐ DK *(Sijui)* ☐ N/A *(Si Husika)* ☐
**Q20 (b).** If YES, what are the deadlines for submitting PC-NTDs surveillance reports to the county level?<br>*(Kama NDIO, je tarehe za mwisho za kutuma ripoti hizi ni zipi?)*	☐ Beginning of every week *(Mwanzo wa kila wiki)*<br>☐ End of every week *(Mwisho wa kila wiki)*<br>☐ Beginning of every month *(Mwanzo wa kila mwezi)*<br>☐ End of every month *(Mwisho wa kila mwezi)*<br>☐ End of every quarter *(Mwisho wa kila robo wa mwaka)*<br>☐ End of every year *(Mwisho wa kila mwaka)*<br>☐ DK *(Sijui)*<br>☐ N/A *(Si Husika)*
**Q20 (c).** Do you often meet the deadlines for submission of PC-NTDs surveillance reports to the county level?<br>*(Je munafaulu kwa mara nyingi kutuma ripoti hizi za ufuatiliaji wa magonjwa ya kitropiki yaliyo telekezwa kueleka kiwango cha kaunti kwa wakati mwafaka?)*	Yes *(Ndio)* ☐ No *(La)* ☐ DK *(Sijui)* ☐ N/A *(Si Husika)* ☐
**Q20 (d).** If NO, what are the reasons for not meeting the deadlines?<br>*(Kama LA, sababu ni zipi?)*	
**Q21 (a).** Are the PC-NTDs surveillance reports from the health facilities accurate?<br>*(Je, ripoti za ufuatiliaji wa magonjwa ya kitropiki yaliyo telekezwa kutoka kwa viwango vya vituo vya afya huwa sahihi?)*	Yes *(Ndio)* ☐ No *(La)* ☐ DK *(Sijui)* ☐ N/A *(Si Husika)* ☐
**Q21 (b).** If NO, why do you say so?<br>*(Kama LA, sababu ni zipi?)*	
**Q22 (a).** Who prepares PC-NTDs surveillance reports in this county?<br>*(Nani anahusika na utayarishaji wa ripoti hizi za ufuatiliaji wa magonjwa ya kitropiki yaliyo telekezwa katika kaunti hii ndogo?)*	☐ County Disease Surveillance Coordinator<br>*(Msimamizi wa shughuli za ufuatiliaji wa magonjwa katika kaunti)*<br>☐ County Public Health Nurse<br>*(Msimamizi wa wauguzi wa afya ya jamii katika kaunti)*

	☐ County Health Records and Information Officer *(Msimamizi wa habari za afya katika kaunti)*<br>☐ County Public Health Officer *(Msimamizi wa afya ya umma katika kaunti)*<br>☐ Other cadre *(Wenye wajibu zinginezo):* ……………………….<br>☐ DK *(Sijui)*<br>☐ N/A *(Si Husika)*
**Q22 (b).** Is he/she trained in preparing PC-NTDs surveillance reports?<br>*(Je, anaye husika na utayarishaji wa ripoti hizi za ufuatiliaji wa magonjwa ikiwemo ya kitropiki yaliyo telekezwa amepata mafunzo maalum?)*	Yes *(Ndio)* ☐ No *(La)* ☐ DK *(Sijui)* ☐ N/A *(Si Husika)* ☐
**Q23 (a).** Is there a standard surveillance reporting format for PC-NTDs used in this county?<br>*(Je, kuna muundo maluum unaotumika katika kaunti hii ndogo wa kuripoti magonjwa haya ya kitropiki yaliyo telekezwa?)*	Yes *(Ndio)* ☐ No *(La)* ☐ DK *(Sijui)* ☐ N/A *(Si Husika)* ☐
**Q23 (b).** If NO, why is this so?<br>*(Kama LA, sababu ni zipi?)*	
**Q24 (a).** Is "zero-reporting" done for instances where there were no reportable PC-NTD cases at a given time in this county?<br>(*Je ripoti za sufuri zinawasilishwa kwa mfano pasipo magonjwa haya ya kitropiki yaliyo telekezwa kwa wakati wowote katika kaunti hii ndogo?)*	Yes *(Ndio)* ☐ No *(La)* ☐ DK *(Sijui)* ☐ N/A *(Si Husika)* ☐
**Q24 (b).** If NO, why is this not done?<br>*(Kama LA, sababu ni zipi?)*	
**Q25 (a).** Through which channel/s do you report PC-NTDs surveillance data from this county to the county level?<br>*(Je, unatumia njia zipi kuripoti habari za ufuatiliaji wa magonjwa haya ya kitropiki yaliyo telekezwa kuzielekeza katika kiwango cha juu?)*	☐ In Person *(Katika mtu/Mwenyewe)*<br>☐ Phone Call *(Kupiga simu)*<br>☐ Mobile SMS *(Kutuma ujumbe fupi kwa kutumia simu ya rununu)*<br>☐ Email *(Kutumia barua pepe)*<br>☐ eIDSR/DHIS *(Njia za kielektroniki)*<br>☐ Other *(Njia zinginezo)*: ……………………………..<br>☐ N/A
**Q25 (b).** Are there challenges experienced reporting PC-NTDs surveillance data through this channel/s?<br>*(Je, kuna changamoto zozote zinazo kumba njia hizi za kuripoti habari za ufuatiliaji wa magonjwa ya kitropiki yaliyo telekezwa ulizotaja?)*	Yes *(Ndio)* ☐ No *(La)* ☐ DK *(Sijui)* ☐ N/A *(Si Husika)* ☐
**Q25 (c).** If YES, what are some of these challenges?<br>*(Kama NDIO, tafadhali taja changamoto hizi)*	
**Q26.** In your own opinion, how can reporting of PC-NTDs surveillance data be improved in this county?	

(Kwa maoni yako binafsi, kuripoti kwa habari za ufuatiliaji wa magonjwa ya kitropiki yaliyo telekezwa zinaweza kuimarishwa kwa njia zipi katika kaunti hii ndogo?)	
**Q27 (a).** Is the existing electronic system adequate for reporting NTDs surveillance data?<br>*(Je, mfumo wa kielektroniki inauwezo tosha wa kuripoti habari za ufuatiliaji wa magonjwa ya kitropiki yaliyo telekezwa?)*	
**Q27 (b).** If NO, why do you say so?<br>*(Kama LA, sababu ni zipi?)*	
**Q28 (a).** Do you find it easy to report NTDs surveillance data through the electronic system?<br>*(Je, mfumo huu wa kielektroniki inarahisisha kuripoti habari za ufuatiliaji wa magonjwa ya kitropiki yaliyo telekezwa?)*	
**Q28 (b).** If NO, why is this so?<br>*(Kama LA, sababu ni zipi?)*	
**Q29 (a).** Have you experienced any challenges reporting NTDs surveillance data using the existing electronic reporting system?<br>*(Je, kuna changamoto zozote zinazo kumba mfumo huu wa kielektroniki wa kuripoti habari za ufuatiliaji wa magonjwa ya kitropiki yaliyo telekezwa?)*	
**Q29 (b).** If YES, what are some of these challenges?<br>*(Kama NDIO, tafadhali taja changamoto hizi)*	
**Q30.** In your own opinion, how can NTDs surveillance data reporting through the electronic system be improved in this county?<br>*(Kwa maoni yako binafsi, kuripoti kwa habari za ufuatiliaji kupitia mfumo wa kielektroniki wa magonjwa ya kitropiki yaliyo telekezwa zinaweza kuimarishwa kwa njia zipi katika kaunti hii?)*	
**DATA ANALYSIS *(UCHANGANUZI WA HABARI ZA UFUATILIAJI WA MAGONJWA)***	
**Q31 (a).** Do you analyse surveillance data for PC-NTDs at this county level?<br>*(Je, kwa kawaida huwa munachanganua habari za ufuatiliaji wa magonjwa ya kitropiki yaliyo telekezwa katika kaunti hii ndogo?*	Yes *(Ndio)* ☐ No *(La)* ☐ DK *(Sijui)* ☐ N/A *(Si Husika)* ☐
**Q31 (b).** If YES, how is the data analysed?<br>*(Kama NDIO, munachanganua habari hizi mulizo kusanya kwa njia zipi?)*	☐ By age *(Kwa umri)*<br>☐ By sex *(Kwa jinsia)*<br>☐ By place *(Kwa makao)*<br>☐ By time *(Kwa saa)*<br>☐ Others *(Kwa njia zinginezo):……………………………*<br>☐ N/A
**Q32 (a).** Does this county perform trend analysis for PC-NTD cases reported from the health facilities?	Yes *(Ndio)* ☐ No *(La)* ☐ DK *(Sijui)* ☐ N/A *(Si Husika)* ☐

(Je, munachanganua mienendo ya magonjwa haya ya kitropiki yaliyo telekezwa katika kaunti hii ndogo?)	
**Q32 (b).** If NO, why is this not done?<br>*(Kama LA, sababu ni zipi?)*	
**Q33 (a).** Do you have an action threshold for the PC-NTDs reported in this county?<br>*(Je, munavizingiti vya matendo katika kaunti hii ndogo inayofuata kuripotiwa kwa magonjwa haya ya kitropiki yaliyo telekezwa?)*	Yes *(Ndio)* ☐ No *(La)* ☐ DK *(Sijui)* ☐ N/A *(Si Husika)* ☐
**Q33 (b).** If YES, what was the action threshold for PC-NTDs reported in the past year?<br>*(Kama NDIO, je vizingiti hivi vya matendo ambavyo vilifuata kuripotiwa kwa magonjwa ya kitropiki yaliyo telekezwa kwa mwaka uliyopita ni yapi?)*	☐ Number of cases *(Nambari ya kesi za magonjwa)*<br>☐ Percentage increase in number of cases *(Ongezeko wa asilimia fulani ya kesi za magonjwa)*<br>☐ Rates *(Kulingana na viwango maalum)*<br>☐ N/A
**Q33 (c).** What action followed the last PC-NTD/s reported from the health facilities, which met the action threshold in this county in the past year?<br>*(Je, kwa mwaka uliyopita ni matendo yapi yalifuatilia kuripotiwa kwa magonjwa ya kitropiki yaliyo telekezwa ambazo zilitimia vizingiti vya matendo yaliyowekwa?)*	……………………………………………………………………..<br>☐ DK *(Sijui)* ☐ N/A *(Si husika)*
**Q34 (a).** Are you satisfied with the analysis done for PC-NTDs surveillance data at this county level?<br>*(Je, umeridhishwa na uchanganuzi wa habari za ufuatiliaji wa magonjwa haya ya kitropiki yaliyo telekezwa katika kaunti hii ndogo?)*	Yes *(Ndio)* ☐ No *(La)* ☐ DK *(Sijui)* ☐ N/A *(Si Husika)* ☐
**Q34 (b).** If NO, why do you so?<br>*(Kama LA, sababu ni zipi?)*	
**Q35 (a).** Are there challenges facing analysis of PC-NTDs surveillance data at this county level?<br>*(Je, kuna changamoto zozote zinazokumba uchanganuzi wa habari za ufuatiliaji wa magonjwa haya ya kitropiki yaliyo telekezwa katika kaunti hii ndogo?)*	Yes *(Ndio)* ☐ No *(La)* ☐ DK *(Sijui)* ☐ N/A *(Si Husika)* ☐
**Q35 (b).** If YES, what are some of these challenges?<br>*(Kama NDIO, tafadhali taja changamoto hizi)*	
**Q36.** In your own opinion, what can be done to improve PC-NTDs surveillance data analysis in this county?<br>*(Kwa maoni yako binafsi, uchanganuzi wa habari za ufuatiliaji wa magonjwa haya ya kitropiki yaliyo telekezwa zinaweza kuimarishwa kwa njia zipi katika kaunti hii ndogo?)*	
**CASE INVESTIGATIONS & EPIDEMIC RESPONSE *(UCHUNGUZI WA KESI ZA MAGONJWA NA MWITIKIO WA MKURUPUKO WA MAGONJWA)***	
**Q37 (a).** Are PC-NTDs reported cases investigated at this county level?	Yes *(Ndio)* ☐ No *(La)* ☐ DK *(Sijui)* ☐ N/A *(Si Husika)* ☐

(Je, kesi za magonjwa ya kitropiki yaliyo telekezwa yanachunguzwa katika kaunti hii ndogo?)	
**Q37 (b).** If NO, why is this so?<br>*(Kama LA, sababu ni zipi?)*	
**Q37 (c).** If YES, describe case investigation for an PC-NTD reported to this county level in the past one year.<br>*(Kama NDIO, eleza kwa kikamilifu uchunguzi uliyofanywa ya moja wapo ya kesi ya ugonjwa wa kitropiki iliyo telekezwa katika kaunti hii ndogo kwa mwaka uliopita?)*	
**Q37 (d).** What action/s followed the case investigation?<br>*(Je, ni hatua zipi zilifuatilia uchunguzi wa kesi hizi?)*	
**Q37 (e).** Was the PC-NTD case investigation guided by IDSR standard guidelines?<br>*(Je, uchunguzi huu ulielekezwa kutokana na mfumo maalum wa IDSR?)*	Yes *(Ndio)* ☐ No *(La)* ☐ DK *(Sijui)* ☐ N/A *(Si Husika)* ☐
**Q37 (f).** If NO, why is this so?<br>*(Kama LA, sababu ni zipi?)*	
**Q38 (a).** Is there a rapid response team that responds to PC-NTDs outbreaks in this county?<br>*(Je, kuna timu maalum ya kuzingatia mwitikio wa mkurupuko wa magonjwa ya kitropiki yaliyo telekezwa katika kaunti hii ndogo?)*	Yes *(Ndio)* ☐ No *(La)* ☐ DK *(Sijui)* ☐ N/A *(Si Husika)* ☐
**Q38 (b).** Are there adequate supplies in this county to respond to PC-NTDs outbreaks?<br>*(Je, kuna vifaa vya kutosha vya kuwezesha mwitikio wa mkurupuko wa magonjwa ya kitropiki yaliyo telekezwa katika kaunti hii ndogo?)*	Yes *(Ndio)* ☐ No *(La)* ☐ DK *(Sijui)* ☐ N/A *(Si Husika)* ☐
**FEEDBACK *(MAONI)***	
**Q39 (a).** Does this county receive feedback on PC-NTDs reports sent to the higher level?<br>*(Je, kaunti hii ndogo inapokea maoni kufuatilia ripoti za ufuatiliaji wa magonjwa ya kitropiki yaliyo telekezwa yaliyotumwa kuelekea kiwango cha juu?)*	Yes *(Ndio)* ☐ No *(La)* ☐ DK *(Sijui)* ☐ N/A *(Si Husika)* ☐
**Q39 (b).** If YES, how many PC-NTDs feedback reports has this county received from the higher level in the past one year?<br>*(Kama NDIO, je kwa mwaka uliopita mulipokea ripoti ngapi za maoni katika kaunti hii ndogo kufuatilia kutuma ripoti za ufuatiliaji wa magonjwa ya kitropiki yaliyo telekezwa kwa kiwango cha juu?)*	☐ 1-2 *(Moja au Miwili)*<br>☐ 3 or more *(Tatu au Zaidi)*<br>☐ Don’t Know *(Sijui)*<br>☐ N/A *(Si Husika)*
**Q40 (a).** Do you feel feedback from the higher level regarding PC-NTDs is important to this county?<br>*(Je, unahisi ya kwamba maoni manazopokea kutoka kiwango cha juu kuhusiana na ufuatiliaji wa magonjwa ya kitropiki yaliyo telekezwa yana manufaa kwenyu katika kaunti hii ndogo?)*	Yes *(Ndio)* ☐ No *(La)* ☐ DK *(Sijui)* ☐ N/A *(Si Husika)* ☐
**Q40 (b).** If NO, why do you say so?<br>*(Kama LA, sababu ni zipi?)*	

**Q40 (c).** If YES, how can feedback from the higher level to this county be improved?<br>*(Kama NDIO, je ripoti za maoni kutoka viwango vya juu vinaweza kuimarishwa na wasimamizi kwa njia zipi?)*	
**Q41 (a).** Do you conduct feedback meetings regarding PC-NTDs with health facilities reporting PC-NTDs surveillance data to this county level?<br>*(Je, mufanya mikutano ya maoni na baadhi ya vituo vya afya vinavyo ripoti magonjwa haya ya kitropiki yaliyo telekezwa?)*	Yes *(Ndio)* ☐ No *(La)* ☐ DK *(Sijui)* ☐ N/A *(Si Husika)* ☐
**Q41 (b).** If YES, how many feedback meetings have been held in the past one year?<br>*(Kama NDIO, je mumeandaa mikutano ngapi ya maoni na baadhi ya vituo hivi vya afya kwa mwaka uliopita?)*	☐ None *(Hamna)*<br>☐ 1-2 *(Mara moja au miwili)*<br>☐ 3 or more (*Mara tatu au zaidi)*<br>☐ Don't Know *(Sijui)*<br>☐ N/A *(Si Husika)*
**Q41 (c).** If NO, why is this so?<br>*(Kama LA, sababu ni zipi?)*	
**Q42 (a).** Does this county send written PC-NTDs feedback reports to the health facilities reporting to this level?<br>*(Je, kiwango hiki cha kaunti kinatuma ripoti za maoni kuhusiana na magonjwa ya kitropiki yaliyo telekezwa kwa vituo vya afya vinavyo ripoti kwa kiwango hiki?*	Yes *(Ndio)* ☐ No *(La)* ☐ DK *(Sijui)* ☐ N/A *(Si Husika)* ☐
**Q42 (b).** If NO, why is this so?<br>*(Kama LA, sababu ni zipi?)*	
**Q42 (c).** If YES, are the written feedback reports to the health facilities produced according to a standard guideline?<br>*(Kama NDIO, je ripoti hizi za maoni zinatengenezwa kwa mtindo maalum?)*	Yes *(Ndio)* ☐ No *(La)* ☐ DK *(Sijui)* ☐ N/A *(Si Husika)* ☐
**Q42 (d).** How many feedback reports regarding PC-NTDs has this county disseminated to the health facilities in the past one year?<br>*(Je, kwa mwaka uliopita kiwango hiki kilisambaza ripoti ngapi za maoni kuhusu magonjwa ya kitropiki yaliyo telekezwa kuelekea kwa viwango vya vituo vya afya katika kaunti hii ndogo?)*	☐ None *(Hamna)*<br>☐ 1-2 *(Moja au Miwili)*<br>☐ 3 or more *(Tatu au Zaidi)*<br>☐ Don't Know *(Sijui)*<br>☐ N/A *(Si Husika)*
**Q42 (e).** Are there challenges in disseminating PC-NTDs feedback reports to the health facilities reporting to this county level?<br>(*Je, kuna changamoto zozote zinazo kumba kusambazwa kwa ripoti za maoni kuhusu magonjwa ya kitropiki yaliyo telekezwa kuelekea kwa viwango vya vituo vya afya katika kaunti hii ndogo?)*	Yes *(Ndio)* ☐ No *(La)* ☐ DK *(Sijui)* ☐ N/A *(Si Husika)* ☐
**Q42 (f).** If YES, what are some of these challenges?<br>*(Kama NDIO, changamoto hizi ni zipi?)*	

**Q43.** In your own opinion, how can feedback coming from the higher level regarding reported PC-NTDs surveillance data be improved?<br>*(Je, kwa maoni yako binafsi viwango vya juu zaidi vinaweza kuboresha ki vipi kusambaza maoni kuhusu ufuatiliaji wa magonjwa ya kitropiki yaliyo telekezwa zilizo ripotiwa na kiwango hiki?)*	
**Q44.** In your own opinion, how can feedback to health facilities reporting PC-NTDs surveillance data to this level be improved?<br>*(Je, kwa maoni yako binafsi kiwango hiki cha kaunti kinaweza kuboresha ki vipi kusambaza maoni kuhusu ufuatiliaji wa magonjwa ya kitropiki yaliyo telekezwa kueleka kwa viwango vya vituo vya afya vinavoripoti kesi hizi?)*	
**STANDARDS AND GUIDELINES (UBORESHAJI NA VIELEKEZI)**	
**Q45 (a).** Are IDSR guidelines and manuals available in this county?<br>*(Je vielekezi vya ufuatiliaji wa magonjwa kupitia kwa mfumo wa IDSR vinapatikana katika kaunti hii ndogo?)*	
**Q45 (b).** If NO, why is this so?<br>*(Kama LA, sababu ni zipi?)*	
**Q46 (a).** Do the IDSR manuals guide PC-NTDs surveillance activities in this county?<br>*(Je vielekezi hivi vya IDSR vinaelekeza shughuli za ufuatiliaji wa magonjwa ya kitropiki yaliyo telekezwa katika kaunti hii ndogo?)*	Yes *(Ndio)* ☐ No *(La)* ☐ DK *(Sijui)* ☐ N/A *(Si Husika)* ☐
**Q46 (b).** If NO, why is this so?<br>*(Kama LA, sababu ni zipi?)*	
**Q47 (a).** Do you have standard guidelines for PC-NTDs case detection, confirmation, reporting and data analysis in this county?<br>*(Je, muna vielekezo maalum za ugunduzi, kuthibiti, kuripoti na uchanganuzi wa kesi za magonjwa ya kitropiki yaliyo telekezwa katika kaunti hii ndogo?)*	Yes *(Ndio)* ☐ No *(La)* ☐ DK *(Sijui)* ☐ N/A *(Si Husika)* ☐
**Q47 (b).** If NO, why is this so?<br>*(Kama LA, sababu ni zipi?)*	
**SUPERVISION *(USIMAMIZI)***	
**Q48 (a).** Do you receive regular supervisory visits from the higher level in this county?<br>*(Je munapokea usimamizi wa mara kwa mara katika kaunti hii ndogo kutoka kwa maafisa wa viwango vya juu?)*	Yes *(Ndio)* ☐ No *(La)* ☐ DK *(Sijui)* ☐ N/A *(Si Husika)* ☐
**Q48 (b).** If NO, why do think this is so?<br>*(Kama LA, sababu ni zipi?)*	
**Q48 (c).** If YES, how often have you received supervisory visits from the higher level in the past one year?	☐ At least once *(Mara moja)*<br>☐ At least twice *(Mara mbili)*

(Kama NDIO, je kwa mwaka uliopita mulipokea ziara za usimamizi mara ngapi kutoka kwa viwango vya juu?)	☐ More than twice *(Zaidi ya mara mbili)*<br>☐ Quarterly *(Mwisho wa robo wa kila mwaka)*<br>☐ N/A
**Q49 (a).** In the last supervisory visits, were surveillance activities in this county reviewed?<br>(*Je, masuala ya shughuli za ufuatiliaji wa magonjwa yalikaguliwa katika ziara za mwisho za usimamizi katika kiwango hiki cha kaunti?)*	Yes *(Ndio)* ☐ No *(La)* ☐ DK *(Sijui)* ☐ N/A *(Si Husika)* ☐
**Q49 (b).** If NO, why was this so?<br>*(Kama LA, sababu ni zipi?)*	
**Q50 (a).** In the last supervisory visits, were PC-NTDs surveillance activities reviewed or discussed?<br>*(Je, suala za ufuatiliaji wa magonjwa ya kitropiki yaliyo telekezwa zilikaguliwa katika ziara za mwisho za usimamizi?)*	Yes *(Ndio)* ☐ No *(La)* ☐ DK *(Sijui)* ☐ N/A *(Si Husika)* ☐
**Q50 (b).** If NO, why was this so?<br>*(Kama LA, sababu ni zipi?)*	
**Q51 (a).** In the last supervisory visits, did you receive a feedback report on PC-NTDs surveillance activities performance in this county?<br>*(Je, mulipokea ripoti maalum ya maoni kutokana na ufuatiliaji wa magonjwa haya ya kitropiki yaliyo telekezwa baada ya ziara za mwisho za usimamizi katika kiwango hiki cha kaunti?)*	Yes *(Ndio)* ☐ No *(La)* ☐ DK *(Sijui)* ☐ N/A *(Si Husika)* ☐
**Q51 (b).** If NO, why was this so?<br>*(Kama LA, sababu ni zipi?)*	
**Q52 (a).** In the last supervisory visits, were there any recommendations made concerning PC-NTDs surveillance in this county in the last supervisory visits?<br>*(Je, kulikuwepo na mapendekezo zozote kuhusu ufuatiliaji wa magonjwa ya kitropiki yaliyo telekezwa wakati wa ziara za mwisho za usimamizi katika kiwango hiki cha kaunti?)*	Yes *(Ndio)* ☐ No *(La)* ☐ DK *(Sijui)* ☐ N/A *(Si Husika)* ☐
**Q52 (b).** If YES, what were some of the recommendations made?<br>*(Kama NDIO, je mapendekezo hizo zilikuwa zipi?)*	
**Q52 (c).** In the last supervisory visits, were there any follow-ups on recommendations made from a previous visit?<br>*(Je, kulikuwepo na ufuatiliaji wowote wa mapendekezo yaliyotolewa kuhusu ufuatiliaji wa magonjwa ya kitropiki yaliyo telekezwa wakati wa ziara za mwisho za usimamizi katika kiwango hiki cha kaunti?)*	Yes *(Ndio)* ☐ No *(La)* ☐ DK *(Sijui)* ☐ N/A *(Si Husika)* ☐
**Q52 (d).** If YES, what did the follow-up entail?<br>*(Kama NDIO, je ufuatiliaji huu uligusia nini haswa?)*	

**Q53 (a).** Are supervisory visits regarding disease surveillance activities conducted in the health facilities reporting to this county level?<br>*(Je, kiwango hiki cha kaunti kina tekeleza ziara za usimamizi kuhusu shughuli za ufuatiliaji wa magonjwa katika viwango vya vituo vya afya vinavyoripoti katika kiwango hiki?)*	Yes *(Ndio)* ☐ No *(La)* ☐ DK *(Sijui)* ☐ N/A *(Si Husika)* ☐
**Q53 (b).** If NO, why is this so?<br>*(Kama LA, sababu ni zipi?)*	
**Q53 (c).** If YES, how often were supervisory visits regarding disease surveillance conducted in the health facilities reporting to this level in the past one year?<br>*(Kama NDIO, je kiwango hiki kilitekeleza kwa mara ngapi ziara hizi za usimamizi wa shughuli za ufuatiliaji wa magonjwa katika viwango vya vituo vya afya?)*	☐ At least once *(Mara moja)*<br>☐ At least twice *(Mara mbili)*<br>☐ More than twice *(Zaidi ya mara mbili)*<br>☐ Quarterly *(Mwisho wa robo wa kila mwaka)*<br>☐ N/A
**Q53 (d).** What is the recommended number of supervisory visits to the health facilities in a period of one year?<br>*(Je, inapendekezwa kiwango hiki cha kaunti kitimize shughuli za ziara za usimamizi katika viwango vya vituo vya afya kwa mara ngapi kwa mwaka?)*	☐ At least once *(Mara moja)*<br>☐ At least twice *(Mara mbili)*<br>☐ More than twice *(Zaidi ya mara mbili)*<br>☐ Quarterly *(Mwisho wa robo wa kila mwaka)*<br>☐ N/A
**Q54 (a).** In the last supervisory visits to the health facilities reporting to this level, were PC-NTDs surveillance data reviewed?<br>*(Katika ziara za mwisho za usimamizi katika viwango vya vituo vya afya, je shughuli za ufuatiliaji wa magonjwa ya kitropiki yaliyo telekezwa zilikaguliwa?)*	Yes *(Ndio)* ☐ No *(La)* ☐ DK *(Sijui)* ☐ N/A *(Si Husika)* ☐
**Q54 (b).** If NO, why was this so?<br>*(Kama LA, sababu ni zipi?)*	
**Q54 (c).** If YES, was a supervision feedback report on PC-NTDs surveillance activities performance sent to the health facilities?<br>*(Je mulipokeza viwango vya vituo vya afya na ripoti maalum kufuatia ziara za mwisho za usimamizi wa shughuli za ufuatiliaji wa magonjwa ya kitropiki yaliyo telekezwa?)*	Yes *(Ndio)* ☐ No *(La)* ☐ DK *(Sijui)* ☐ N/A *(Si Husika)* ☐
**Q54 (d).** If NO, why was this so?<br>*(Kama LA, sababu ni zipi?)*	
**Q55 (a).** Is there a schedule/plan for conducing future supervisory visits in the health facilities reporting to this county level?<br>*(Je, katika kiwago hiki cha kaunti kuna ratiba maalum ya kutekeleza ziara za usimamizi katika viwango vya vituo vya afya kwa siku zijazo?)*	Yes *(Ndio)* ☐ No *(La)* ☐ DK *(Sijui)* ☐ N/A *(Si Husika)* ☐
**Q55 (b).** If NO, why is this so?<br>*(Kama LA, sababu ni zipi?)*	

**Q56 (a).** Do you experience challenges conducting supervisory activities in the health facilities level? *(Je, munapata changamoto zozote munapo tekeleza ziara za usimamizi katika viwango vya vituo vya afya?)*	Yes *(Ndio)* ☐ No *(La)* ☐ DK *(Sijui)* ☐ N/A *(Si Husika)* ☐
**Q56 (b).** If YES, what are some of these challenges? *(Kama NDIO, je changamoto hizi ni zipi?)*	
**Q57.** In your own opinion, how can supervision of PC-NTDs surveillance activities from the higher level to this county be improved? *(Je, kwa maoni yako binafsi, viwango vya juu zaidi vinaweza kuboresha ki vipi ziara za usimamizi wa shughuli za ufuatiliaji wa magonjwa ya kitropiki yaliyo telekezwa katika kiwango hiki cha kaunti?)*	
**Q58.** In your own opinion, how can supervision of PC-NTDs surveillance activities in the health facilities by this county be improved? *(Je, kwa maoni yako binafsi, kiwango hiki kinaweza kuboresha ki vipi ziara za usimamizi wa shughuli za ufuatiliaji wa magonjwa ya kitropiki yaliyo telekezwa katika viwango vya vituo vya afya?)*	
**TRAINING *(MAFUNZO)***	
**Q59 (a).** In your basic training were you trained on disease surveillance? *(Je, katika elimu yako ya msingi ulipata mafunzo ya ufuatiliaji wa magonjwa?)*	Yes *(Ndio)* ☐ No *(La)* ☐ DK *(Sijui)* ☐ N/A *(Si Husika)* ☐
**Q59 (b).** If NO, why was this so? *(Kama LA, sababu ni zipi?)*	
**Q60 (a).** Do you feel your basic training is sufficient for you to adequately supervise disease surveillance activities in this county?	Yes *(Ndio)* ☐ No *(La)* ☐ DK *(Sijui)* ☐ N/A *(Si Husika)* ☐
**Q60 (b).** If NO, why do you say so? *(Kama LA, sababu ni zipi?)*	
**Q61 (a).** Is your basic training applicable to PC-NTDs surveillance in this county? *(Je, unahisi mafunzo yako ya msingi uliyopata yalikuwa ya kutosha kutimiza ufuatiliaji wa magonjwa katika kiwango hiki cha kaunti?)*	Yes *(Ndio)* ☐ No *(La)* ☐ DK *(Sijui)* ☐ N/A *(Si Husika)* ☐
**Q61 (b).** If NO, why do you say so? *(Kama LA, sababu ni zipi?)*	
**Q62 (a).** While working in your current designation have you received any post basic training on disease surveillance in the past one year? *(Je, kwa uajibu wako kikazi katika kiwango hiki umepata mafunzo zaidi maalum kuhusiana na ufuatiliaji wa magonjwa kando na elimu yako ya msingi kwa mwaka uliopita?)*	Yes *(Ndio)* ☐ No *(La)* ☐ DK *(Sijui)* ☐ N/A *(Si Husika)* ☐
**Q62 (b).** If YES *(Kama NDIO)*,	…………………………………………………..☐ N/A

When were you last trained *(Ulipata mafunzo hayo ya mwisho lini?)*<br><br>Where was the training *(Ulipata mafunzo hayo wapi?)*<br><br>Who facilitated the training *(Nani aliwezesha mafunzo hayo?)*<br><br>What was the duration of the training *(Mafunzo hayo yalikua kwa muda gani?)*	…………………………………………………..☐ N/A<br><br>…………………………………………………..☐ N/A<br><br>…………………………………………………..☐ N/A
**Q62 (c).** Which specific elements of disease surveillance and response elements were covered in your last post-basic training?<br>*(Je mafunzo hayo yaliangazia mambo yapi haswa kuhusu ufuatiliaji wa magonjwa?)*	☐ Case Detection *(Ugunduzi wa kesi za magonjwa)*<br>☐ Case Registration *(Usajili wa kesi za magonjwa)*<br>☐ Case Confirmation *(Uthibitisho wa kesi za magonjwa)*<br>☐ Reporting *(Kuripoti kesi za magonjwa)*<br>☐ Data Analysis *(Uchanganuzi wa kesi za magonjwa)*<br>☐ Outbreak Investigation *(Uchunguzi wa kuzuka kwa magonjwa)*<br>☐ Response and Control *(Uajibu na udhibiti wa mgonjwa)*<br>☐ Others *(Mada zinginezo)*:…………………………….<br>☐ DK *(Sijui)*<br>☐ N/A *(Si Husika)*
**Q62 (d).** In your last post-basic training, was disease surveillance and response specific to PC-NTDs covered?<br>*(Je, mafunzo hayo ya mwisho yaliangazia ufuatiliaji wa magonjwa ya kitropiki yaliyo telekezwa?)*	Yes *(Ndio)* ☐ No *(La)* ☐ DK *(Sijui)* ☐ N/A *(Si Husika)* ☐
**Q62 (e).** If YES, which specific elements regarding PC-NTDs surveillance and response were covered in the training?<br>*(Kama NDIO, je ni mambo yapi haswa yaliangaziwa kuhusiana na ufuatiliaji wa magonjwa ya kitropiki yaliyo telekezwa?)*	☐ Case Detection *(Ugunduzi wa kesi za magonjwa)*<br>☐ Case Registration *(Usajili wa kesi za magonjwa)*<br>☐ Case Confirmation *(Uthibitisho wa kesi za magonjwa)*<br>☐ Reporting *(Kuripoti kesi za magonjwa)*<br>☐ Data Analysis *(Uchanganuzi wa kesi za magonjwa)*<br>☐ Outbreak Investigation *(Uchunguzi wa kuzuka kwa magonjwa)*<br>☐ Response and Control *(Uajibu na udhibiti wa mgonjwa)*<br>☐ Others *(Mada zinginezo)*:…………………………….<br>☐ DK *(Sijui)*<br>☐ N/A *(Si Husika)*
**Q62 (f).** If NO, would you be interested in being trained specifically on PC-NTDs surveillance and response?<br>*(Kama LA, je unania ya mafunzo inayoangazia ufuatiliaji wa magonjwa ya kitropiki yaliyo telekezwa?)*	Yes *(Ndio)* ☐ No *(La)* ☐ DK *(Sijui)* ☐ N/A *(Si Husika)* ☐
**Q62 (g).** If YES, which specific aspect/s regarding PC-NTDs surveillance and response would you like	☐ Case Detection *(Ugunduzi wa kesi za magonjwa)*

the training to focus on amongst those mentioned in this interview?<br>*(Kama NDIO, je ni mambo yapi haswa ungependa mafunzo hayo ya angazie kuhusiana na ufuatiliaji wa magonjwa ya kitropiki yaliyo telekezwa?)*	☐ Case Registration *(Usajili wa kesi za magonjwa)*<br>☐ Case Confirmation *(Uthibitisho wa kesi za magonjwa)*<br>☐ Reporting *(Kuripoti kesi za magonjwa)*<br>☐ Data Analysis *(Uchanganuzi wa kesi za magonjwa)*<br>☐ Outbreak Investigation *(Uchunguzi wa kuzuka kwa magonjwa)*<br>☐ Response and Control *(Uajibu na udhibiti wa mgonjwa)*<br>☐ Others *(Mada zinginezo)*:…………………………….<br>☐ DK (Sijui)<br>☐ N/A
**Q63 (a).** Are there any challenges facing post-basic training of health personnel in this county?<br>*(Je, kuna chamgamoto zozote zinazokumba maandalizi ya mafunzo ya wafanyakazi wa afya katika kiwango hiki cha kaunti?)*	Yes *(Ndio)* ☐ No *(La)* ☐ DK *(Sijui)* ☐ N/A *(Si Husika)* ☐
**Q63 (b).** If YES, what are some of these challenges?<br>*(Kama NDIO, je changamoto hizi ni zipi?)*	
**Q64.** In your own opinion, how can post-basic training on PC-NTDs surveillance and response be improved in this county?<br>*(Kwa maoni yako binafsi, je mafunzo yanayoangazia ufuatiliaji wa magonjwa ya kitropiki yaliyo telekezwa zinaeza kuboreshwa ki vipi katika kiwango hiki cha kaunti?)*	
**DISEASE SURVEILLANCE COORDINATION *(URATIBU WA UFUATILIAJI WA MAGONJWA)***	
**Q65 (a).** Is there an existing disease surveillance coordination committee in this county?<br>*(Je, kuna kamati ya kuratibu shughuli za ufuatiliaji wa magonjwa katika kaunti hii?)*	
**Q65 (b).** If NO, why is this so?<br>*(Kama LA, sababu ni zipi?)*	
**Q65 (c).** If YES, does the committee coordinate surveillance activities relating to NTDs?<br>*(Kama NDIO, je kamati hii inaratibu shughuli za ufuatiliaji kuhusiana na magonjwa za kitropiki zilizo telekezwa?)*	
**Q65 (d).** If NO, why is this so?<br>*(Kama LA, sababu ni zipi?)*	
**Q65 (e).** If YES, which NTDs surveillance activities did this committee focus on in the past one year?<br>*(Kama NDIO, je ni shughuli zipi za ufuatiliaji kuhusiana na magonjwa za kitropiki zilizo telekezwa zilitekelezwa na kamati hii kwa mwaka uliopita?)*	
**Q65 (f).** Do you have a role in the county disease surveillance coordination committee?<br>*(Je, una jukumu maalum katika kamati ya kuratibu shughuli za ufuatiliaji wa magonjwa katika kaunti hii?)*	Yes *(Ndio)* ☐ No *(La)* ☐ N/A *(Si Husika)* ☐

**Q65 (g).** If YES, what is your role in this disease surveillance coordination committee?<br>*(Kama NDIO, je jukumu lako ni gani katika kamati hii ya kuratibu shughuli za ufuatiliaji wa magonjwa katika kaunti hii?)*	
**Q65 (h).** Are there challenges facing coordination of NTDs surveillance activities in this county?<br>*(Je, kuna changamoto zozote zinazokumba shughuli za kuratibu ufuatiliaji wa magonjwa ya kitropiki yaliyo telekezwa katika kaunti hii?)*	Yes *(Ndio)* ☐ No *(La)* ☐ DK *(Sijui)* ☐ N/A *(Si Husika)* ☐
**Q65 (i).** If YES, what are some of these challenges?<br>*(Kama NDIO, changamoto hizi ni zipi?)*	
**Q65 (j).** What can be done to improve NTDs surveillance and response activities coordination in this county?<br>*(Je, uratibu wa shughuli za ufuatiliaji wa magonjwa ya kitropiki yaliyo telekezwa katika kaunti hii zinaeza kuboreshwa kwa njia zipi?)*	
**OPPORTUNITIES FOR IMPROVEMENT *(NAFASI ZA UBORESHAJI WA UFUATILIAJI WA MAGONJWA)***	
**Q66 (a).** Are you satisfied with PC-NTDs surveillance and response activities within the IDSR system in this county as it is?<br>*(Je, umeridhishwa na shughuli za ufuatiliaji wa magonjwa ya kitropiki yaliyo telekezwa katika kiwango hiki cha kaunti vile zilivyo?)*	Yes *(Ndio)* ☐ No *(La)* ☐ DK *(Sijui)* ☐ N/A *(Si Husika)* ☐
**Q66 (b).** If NO, what are the main challenges facing PC-NTDs surveillance and response activities within the IDSR system in this county?<br>*(Kama LA, ni changamoto zipi kuu zinazo kumba shughuli za ufuatiliaji wa magonjwa ya kitropiki yaliyo telekezwa katika kiwango hiki cha kaunti?)*	
**Q67.** In your own opinion, what can be done to improve PC-NTDs surveillance and response in this county?<br>*(Kwa maoni yako binafsi, je shughuli hizi za ufuatiliaji wa magonjwa ya kitropiki yaliyo telekezwa zinaweza kuboreshwa kwa njia zipi katika kiwango hiki cha kaunti?)*	
**RESOURCES *(RASILIMALI)***	
**Q68.** Availability of electricity<br>*(Uwepo wa umeme)*	Yes *(Ndio)* ☐ No *(La)* ☐
**Q69.** Availability of computers<br>*(Uwepo wa kompyuta)*	Yes *(Ndio)* ☐ No *(La)* ☐
**Q70.** Availability of telephones/mobile services<br>*(Uwepo wa simu)*	Yes *(Ndio)* ☐ No *(La)* ☐
**Q71.** Availability of internet services<br>*(Uwepo wa mtandao)*	Yes *(Ndio)* ☐ No *(La)* ☐
**Q72.** Availability of PC-NTD posters<br>*(Uwepo wa tangazo za ukutani za magonjwa ya kitropiki yaliyo telekezwa zinaweza)*	Yes *(Ndio)* ☐ No *(La)* ☐

KEY INFORMANT INTERVIEW SCHEDULE

<table>
<tr><td>GENERAL INFORMATION
(Habari kwa ujumla)</td><td colspan="2">Please mark (X) in the appropriate box
(Tafadhali weka alama ya tiki (√) katika sehemu inayofaa zaidi)</td></tr>
<tr><td>Region
(Mkoa)</td><td colspan="2">…………………………………………………………….. County (Kaunti/Mkoa)</td></tr>
<tr><td rowspan="2">Demographic information
(Demografia ya mhojiwa)</td><td>Age (Umri)</td><td>☐ 18-30 ☐ 31-40
☐ 41-50 ☐ >50</td></tr>
<tr><td>Sex (Jinsia)</td><td>☐ Male (Kiume) ☐ Female (Kike)</td></tr>
<tr><td>Q1. What is your current designation at the county level?
(Wajibu wako katika kiwango cha kata hii ni upi?)</td><td colspan="2">☐ County Director of Health (Mkurugenzi wa afya katika kaunti)
☐ County Epidemiologist (Msimamizi wa magonjwa ya mulipuko katika kaunti)
☐ County NTDs Coordinator (Msimamizi wa ufuatilaji wa magonjwa zilizotelekezwa katika kaunti)
☐ County Health Information Officer (Msimamizi wa habari za afya katika kaunti)
☐ County Public Health Officer (Msimamizi wa afya ya umma katika kaunti)
☐ Other (Wajibu zinginezo): ……………………………………………………</td></tr>
<tr><td>Number of years worked in your current designation
(Miaka yako kikazi katika uajibu huu?)</td><td colspan="2">☐ Less than a year (Chini ya mwaka 1)
☐ 1-2 years (Kati ya mwaka 1-2)
☐ 2-3 years (Kati ya miaka 2-3)
☐ 3-5 years (Kati ya miaka 3-5)
☐ More than 5years (Zaidi ya miaka 5)</td></tr>
<tr><td>Q2. What is your highest level of education?
(Kiwango chako cha juu zaidi katika masomo?)</td><td colspan="2">☐ PhD (Shahada ya uzamifu)
☐ Masters (Shahada ya uzamili)
☐ Degree (Shahada)
☐ Diploma (Stashahada)
☐ Certificate (Astashahada)</td></tr>
<tr><td colspan="3">DISEASE SURVEILLANCE & RESPONSE (UFUATILIAJI WA MAGONJWA)</td></tr>
<tr><td colspan="3">Q3. What are your thoughts on disease surveillance and response activities in this region?
(Je, wazo zako ni zipi kuhusu shughuli za ufuatiliaji wa magonjwa katika kaunti hii?)</td></tr>
<tr><td colspan="3"></td></tr>
<tr><td colspan="3">Q4. Please comment on health facility based disease surveillance in this region.
(Tafadhali nipe maoni yako kuhusu ufuatialiaji wa magonjwa kwa vituo vya afya katika kaunti hii?)</td></tr>
<tr><td colspan="3"></td></tr>
</table>

**Q5.** Please comment on community based disease surveillance in this region.<br>(*Tafadhali nipe maoni yako kuhusu ufuatiliaji wa magonjwa kwa kiwango cha jamii katika kaunti hii?)*	
**Q6.** Please comment on the importance of the existing integrated disease surveillance and response (IDSR) system to this county. *(Tafadhali nipe maoni yako kuhusu umuhimu wa mfumo wa IDSR katika kaunti hii)*	
**NEGLECTED TROPICAL DISEASES (NTDs)** ***(MAGOJWA YA KITROPIKI YALIYO TELEKEZWA)***	
**Q7.** Please comment on surveillance and response activities regarding neglected tropical diseases (NTDs) in this region.<br>*(Tafadhali nipe maoni yako kuhusu ufuatiliaji wa magonjwa ya kitropiki yaliyo telekezwa katika mkoa huu)*	
**Q8.** How effective is NTDs surveillance and response undertaken within the IDSR system in this region? Please explain.<br>*(Tafadhali eleza ufanisi wa ufuatiliaji wa magonjwa ya kitropiki yaliyo telekezwa kupitia mfumo wa IDSR)*	
**Q9.** Which are the preventive chemotherapy targeted neglected tropical diseases (PC-NTDs) common in this region?<br>*(Je ni magonjwa yapi ya kitropiki yaliyo telekezwa ambayo ni ya kawaida katika mkoa huu?)*	☐ Lymphatic Filariasis *(Matende)*<br>☐ Soil Transmitted Helminths *(Minyoo)*<br>☐ Trachoma *(Trakoma)*<br>☐ Schistosomiasis *(Kichocho)*<br>☐ Others *(Magonjwa zinginezo)*: ………………………….....<br>☐ Don't Know *(Sijui)*
**Q10.** Which of the above mentioned PC-NTDs are under surveillance within the IDSR system in this region?<br>*(Je ni magonjwa yapi ya kitropiki yaliyo telekezwa baadhi ya yale uliyotaja ambayo yanaripotiwa kupitia mfumo wa IDSR?)*	☐ Lymphatic Filariasis *(Matende)*<br>☐ Soil Transmitted Helminths *(Minyoo)*<br>☐ Trachoma *(Trakoma)*<br>☐ Schistosomiasis *(Kichocho)*<br>☐ Others *(Magonjwa zinginezo)*: ………………………….....<br>☐ Don't Know *(Sijui)*

**CORE FUNCTIONS**
**CASE DETECTION *(UGUNDUZI WA KESI ZA MAGONJWA)***
**Q11.** Is there an existing surveillance system to detect preventive chemotherapy targeted neglected tropical disease (PC-NTD) cases at the health facilities in this region? Please Explain. *(Je kuna mfumo maalum wa kuchunguza magonjwa ya kitropiki yaliyo telekezwa katika vituo vya afya wa mkoa huu? Tafadhali eleza)*
**Q12.** Is there an existing mechanism to capture PC-NTD cases at the community level in this region? Please explain. *(Je kuna utaratibu maalum wa kunasa kesi za magonjwa ya kitropiki yaliyo telekeza katika kiwango cha jamii wa mkoa huu? Tafadhali eleza)*
**Q13.** Do the health personnel involved in disease surveillance activities have the capacity to notify and detect PC-NTD cases in this region? Please explain based on these levels (i.e. sub-county, health facility and community). *(Je wafanyakazi wa afya wanaohusika na shughuli za ufuatiliaji wa magonjwa wanauwezo wa kuchunguza magonjwa ya kitropiki yaliyo telekezwa katika mkoa huu? Tafadhali eleza kulingana na viwango vya kaunti ndogo, vituo vya afya ama wa jamii?)*
**Q14.** In your own opinion, what can be done to improve detection of PC-NTD cases in this region? Please explain based on these levels (i.e. sub-county, health facility and community) *(Je kwa maoni yako ni yapi yanaweza kuboresha uchunguzi wa magonjwa ya kitropiki yaliyo telekezwa katika mkoa huu? Tafadhali eleza kulingana na viwango vya kaunti ndogo, vituo vya afya ama wa jamii?)*
**CASE CONFIRMATION *(UTHIBITISHAJI WA KESI ZA MAGONJWA)***
**Q15.** Do health facilities in this region have the capacity to confirm suspected PC-NTD cases? Please explain. *(Je, vituo vya afya katika mkoa huu vinaweza kuthibitisha tuhumu za kesi za magonjwa ya kitropiki yaliyo telekezwa? Tafadhali eleza)*

**Q16.** Are heath facilities in this region able to refer samples of suspected PC-NTD cases to the next level in a timely manner? Please explain. *(Je, vituo vya afya katika mkoa huu vinaweza kupeleka sampuli za kesi za magonjwa ya kitropiki yaliyo telekezwa katika maabara ya juu kwa wakati unaofaa? Tafadhali eleza)*
**Q17.** Does this region have the capacity to undertake follow-ups on confirmed PC-NTD cases? Please explain. *(Je, mkoa huu una uwezo wa kufuatialia kesi zilizothibitishwa za magonjwa ya kitropiki yaliyo telekezwa? Tafadhali eleza)*
**Q18.** Would you say the capacity to confirm suspected PC-NTD cases at a point in time led to early outbreak detection in this region? Please explain. *(Je unadhani uwezo wa kuthibiti tuhumu za kesi za magonjwa ya kitropiki yaliyo telekezwa katika mkoa huu vimewezesha ugunduzi wa mapema kabla kuzuka kwa magonjwa haya? Tafadhali eleza)*
**SURVEILLANCE DATA REPORTING *(KURIPOTI KWA HABARI ZA UFUATILIAJI WA MAGONJWA)***
**Q19.** Was the proportion of health facilities reporting suspected or confirmed PC-NTD cases to the next level in the past one year adequate? Please explain. *(Je, idadi ya vituo vya afya zilizoripoti kesi za tuhumu za magonjwa ya kitropiki yaliyo telekezwa kwa mwaka uliopita ni za kutosha? Tafadhali eleza)*
**Q20.** In your opinion, was the rate of PC-NTD cases reporting timeliness and completeness in the past one year adequate across the reporting levels in this region? Please comment. *(Kwa maoni yako binafsi, je kiwango cha kuripoti kwa wakati mwafaka na ukamilifu kesi za magonjwa ya kitropiki yaliyo telekezwa katika mwaka uliopita ulikuwa wa kutosha? Tafadhali eleza)*
**Q21.** What could have improved PC-NTD case reporting from the lower to the higher levels in this region in the past one year? Please explain. *(Je, shughuli za kuripoti kesi za magonjwa ya kitropiki yaliyo telekezwa kutoka kwa kiwango cha jamii hadi kiwango cha juu zaidi zinaweza kuimarishwa ki vipi? Tafadhali eleza)*

**DATA ANALYSIS** ***(UCHANGANUZI WA DATA)***
**Q22.** Is surveillance data analysis undertaken to determine pre-defined action thresholds for PC-NTDs reported in this region? Please explain.<br>*(Je munafanya uchanganuzi wa habari zilizoripotiwa za ufuatilaiji wa magonjwa ya kitropiki yaliyo telekezwa ili kuamua vizingiti vya matendo katika mkoa huu? Tafadhali eleza)*
**Q23.** In your own opinion, what could have been done to improve PC-NTDs surveillance data analysis in the past one year? Please explain.<br>*(Je kwa maoni yako ni yapi yangezingatiwa kuimarisha uchanganuzi wa habari za ufuatiliaji wa magonjwa ya kitropiki yaliyo telekezwa kwa mwaka uliopita katika mkoa huu? Tafadhali eleza)*
**EPIDEMIC PREPAREDNESS AND RESPONSE** ***(UTAYARISHAJI NA MWITIKIO WA MKURUPUKO WA MAGONJWA)***
**Q24.** Would you say the health personnel in this region have the capacity to respond to PC-NTDs outbreaks? Please explain based on their competencies.<br>*(Je unaweza sema wafanyakazi wa afya katika mkoa huu wanauwezo wa kutatua mizuko ya magonjwa ya kitropiki yaliyo telekezwa, Tafadhali eleza)*
**Q25.** In your own opinion, how could PC-NTDs outbreak preparedness and response be improved in this region in the past one year?<br>*(Kwa maoni yako, je shughuli za kujiandaa kukabiliana na mizuko ya magonjwa ya kitropiki yaliyo telekezwa kwa mwaka uliopita zingeboreshwa ki vipi?)*
**FEEDBACK** ***(MAONI)***
**Q26.** How effective was the PC-NTDs surveillance feedback mechanism across the surveillance levels in this region in the past one year? Please comment.<br>*(Je, maoni kufuatilia ripoti za ufuatiliaji wa magonjwa ya kitropiki yaliyo telekezwa zilizotumwa kuelekea kiwango moja hadi kingine zilifanywa kwa ufanisi kwa mwaka uliopita? Tafadhali eleza)*

**Q27.** In your own opinion, how could PC-NTDs surveillance feedback be improved in this region in the past one year? *(Kwa maoni yako binafsi, je ripoti za maoni kufuatilia kutumwa kwa habari za ufuatiliaji wa magonjwa ya kitropiki yaliyo telekezwa kutoka kiwango moja hadi kingine zingeboreshwa ki vipi kwa mwaka uliopita? Tafadhali eleza)*
**SUPPORT FUNCTIONS**
**STANDARDS & GUIDELINES *(MIONGOZO)***
**Q28.** In the past one year, how adequate were the disease surveillance standards and guidelines as relates to PC-NTDs in region? Please explain based on case definition guidelines. *(Je kwa mwaka uliopita viwangogezi na miongozo za ufuatiliaji wa magonjwa ya kitropiki yaliyo telekezwa katika mkoa huu zilikuwa za kutosha? Tafadhali eleza kulingana na miongozo za kutambua magonjwa hayo)*
**Q29.** How adequate was the proportion of health facilities utilizing PC-NTDs standard case management guidelines in the past one year? Please comment. *(Je kwa mwaka uliopita idadi ya vituo vya afya zilizotumia miongozo za kutambua kesi za magonjwa ya kitropiki yaliyo telekezwa katika mkoa huu zilikuwa za kutosha? Tafadhali eleza.)*
**TRAINING *(MAFUNZO)***
**Q30.** In the past one year, were PC-NTDs surveillance training manuals available in this region and how adequately were they put to use? Please comment. *(Kwa mwaka uliopita, je miongozo za mafunzo kuhusu magonjwa ya kitropiki yaliyo telekezwa zilipatikana na kutumika katika mkoa huu? Tafadhali eleza)*
**Q31.** Did health personnel in this region receive post-basic training on disease surveillance activities in the past one year? Please comment. *(Je kwa mwaka uliopita wahudumu wa afya katika mkoa huu walipata mafunzo zaidi maalum za shughuli za ufuatiliaji wa magonjwa ya kitropiki yaliyo telekezwa kando na elimu ya msingi? Tafadhali eleza)*

**Q32.** In your own opinion, how could health personnel training on PC-NTDs surveillance be improved in the past one year? Please explain. *(Kwa maoni yako binafsi, je mafunzo kuhusu magonjwa ya kitropiki yaliyo telekezwa zingeweza kuimarishwa ki vipi kwa mwaka uliopita?)*
**SUPERVISION *(USIMAMIZI)***
**Q33.** Were there supervision plans available for conducting supervisory visits regarding disease surveillance activities in this region in the past one year? Please comment.<br>*(Je kwa mwaka uliopita kuliwa na mipango maalum ya kuelekeza usimamizi wa shughuli za ufuatiliaji wa magonjwa katika mkoa huu? Tafadhali eleza)*
**Q34.** In your own opinion, what can be done to improve supervision of PC-NTDs surveillance and response activities across all surveillance levels in this region? Please comment.<br>*(Kwa maoni yako binafsi, je ni mikakati ipi inaweza kuboresha usimamizi wa shughuli za ufuatiliaji wa magonjwa ya kitropiki yaliyo telekezwa katika mkoa huu? Tafadhal eleza)*
**RESOURCES *(RASILIMALI)***
**Q35.** Was there a budget specific for conducting PC-NTDs surveillance and response activities in the past one year in this region? Please comment.<br>*(Je kwa mwaka uliopita, kulikuwepo na bajeti maalum ya kuwezesha shughuli za ufuatiliaji wa magonjwa ya kitropiki yaliyo telekezwa katika mkoa huu? Tafadhali eleza)*
**Q36.** In your own opinion, how could the resources for facilitating PC-NTDs surveillance and response activities have been improved in the past one year? Please comment.<br>*(Je kwa mwaka uliopita rasilimali ya kuwezesha shughuli za ufuatiliaji wa magonjwa ya kitropiki yaliyo telekezwa katika mkoa huu zingeweza kuimarishwa kwa njia zipi? Tafadhali eleza)*

**COORDINATION**
**Q37.** How adequate is the coordination of PC-NTDs surveillance and response activities across the surveillance levels in this region? Please comment.<br>*(Je uratibu wa shughuli za ufuatiliaji wa magonjwa ya kitropiki yaliyo telekezwa katika mkoa huu ni wa kutosha? Tafadhali eleza)*
**Q38.** What could have been done in the past year to improve PC-NTDs surveillance and response activities coordination in this region? Please explain.<br>*(Je kwa mwaka uliopita ni yapi yangeboresha uratibu wa shughuli za ufuatiliaji wa magonjwa ya kitropiki yaliyo telekezwa katika mkoa huu? Tafadhali eleza)*
